# Supplementary material for: Insect-habitat-plant interaction networks provide guidelines to mitigate the risk of transmission of Xylella fastidiosa to grapevine in Southern France
Source: PLoS One. 2025 Sep 15;20(9):e0332344. doi: 10.1371/journal.pone.0332344 (PMC12435670; doi:10.1371/journal.pone.0332344)
Supplement: S1 Appendix — (ZIP) [file pone.0332344.s001.zip › S1_Appendix.pdf]

Appendix S1: Geographical distribution of the sample size

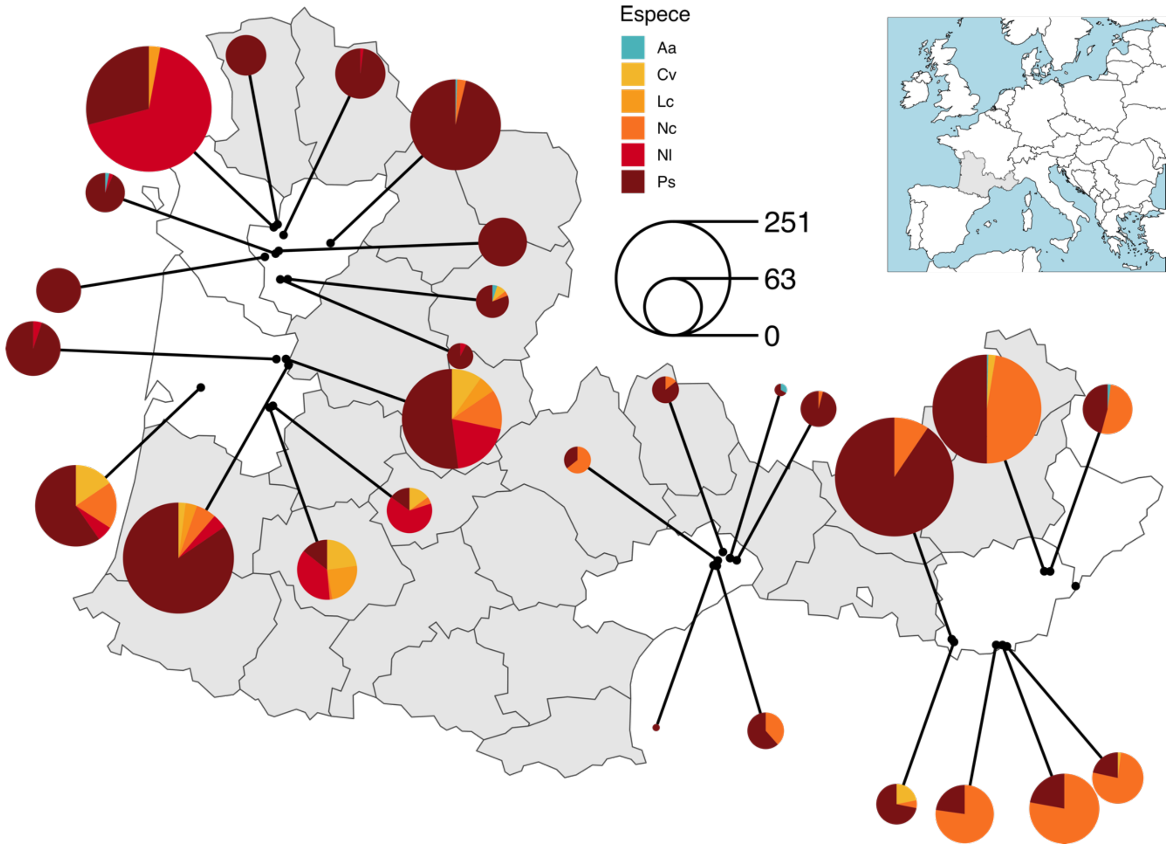

Figure S1.1. Distribution of insects sampled per buffer in the fall 2020. Insect species are abbreviated as follows Aa: *Aphrophora alni*, Agrs: *Aphrophora* grp. *salicina*, Cv: *Cicadella viridis*, Lc: *Lepyronia coleoptrata*, Nc: *Neophilaenus campestris*, Nl: *Neophilaenus lineatus*, Nsp.: *Neophilaenus* sp. and Ps: *Philaenus spumarius*. Country contours were displayed using Natural Earth (version 5.1.1) under a CC0 licence, French departments were displayed using ADMIN EXPRESS database from the National Institute of Geography (IGN), freely available under a CC BY licence.

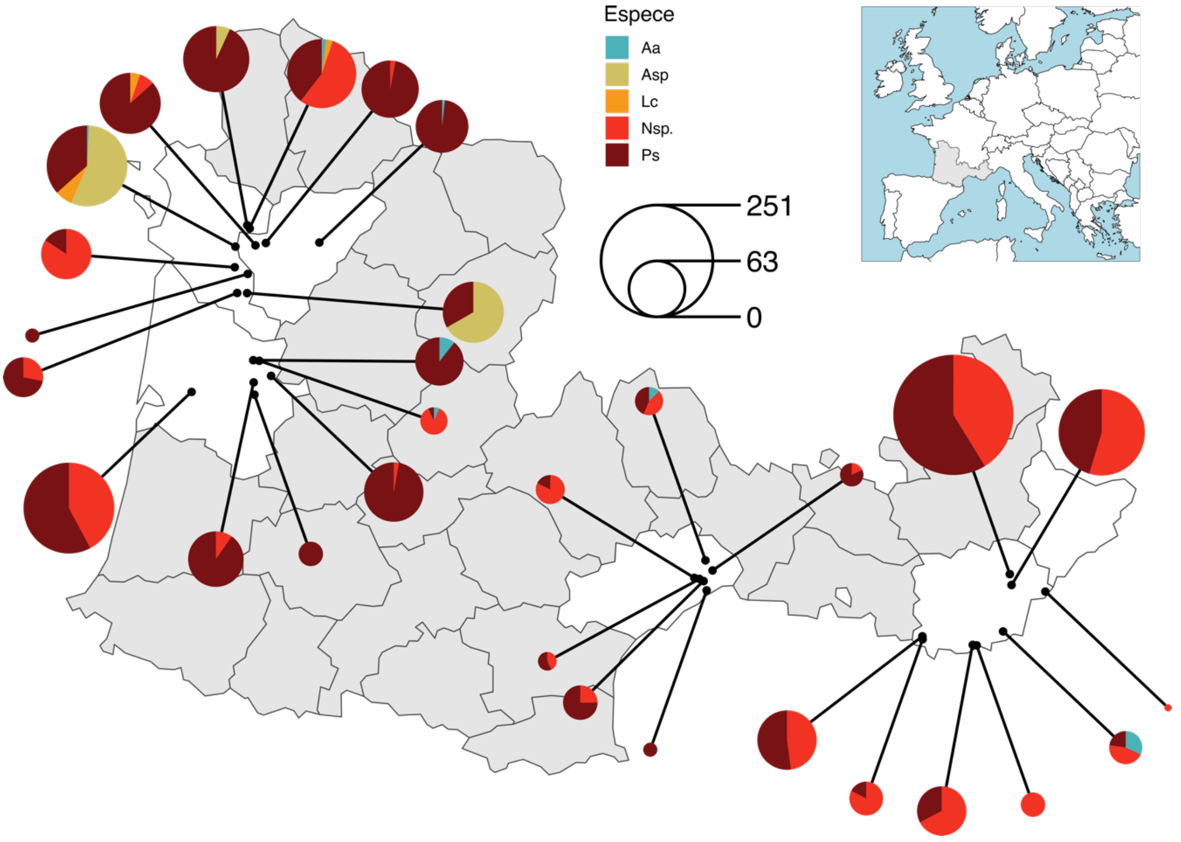

Figure S1.2. Distribution of insects sampled per buffer in the spring 2021. Insect species are abbreviated as follows Aa: *Aphrophora alni*, Agrs: *Aphrophora* grp. *salicina*, Cv: *Cicadella viridis*, Lc: *Lepyronia coleoptrata*, Nc: *Neophilaenus campestris*, Nl: *Neophilaenus lineatus*, Nsp.: *Neophilaenus* sp. and Ps: *Philaenus spumarius*. Country contours were displayed using Natural Earth (version 5.1.1) under a CC0 licence, French departments were displayed using ADMIN EXPRESS database from the National Institute of Geography (IGN), freely available under a CC BY licence.

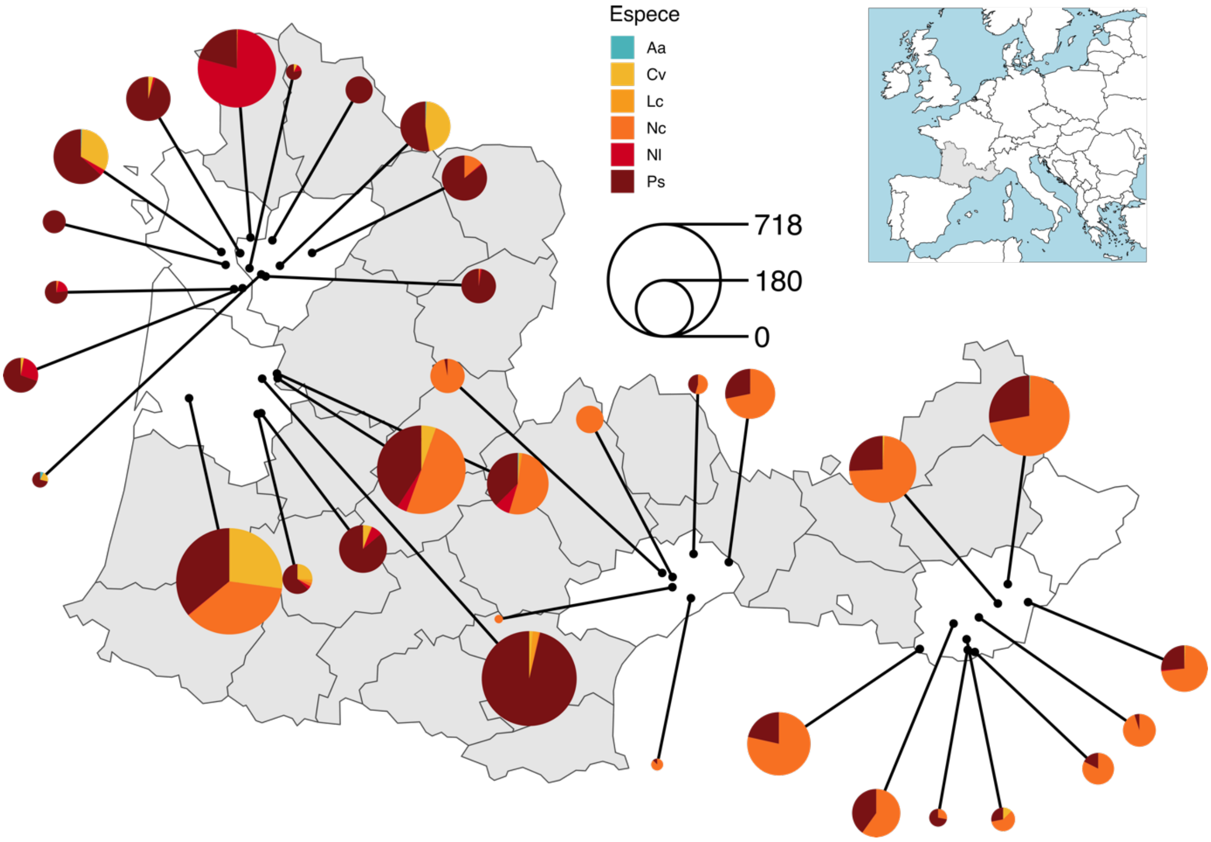

Figure S1.3. Distribution of insects sampled per buffer in the fall 2021. Insect species are abbreviated as follows Aa: *Aphrophora alni*, Agrs: *Aphrophora* grp. *salicina*, Cv: *Cicadella viridis*, Lc: *Lepyronia coleoptrata*, Nc: *Neophilaenus campestris*, Nl: *Neophilaenus lineatus*, Nsp.: *Neophilaenus* sp. and Ps: *Philaenus spumarius*. Country contours were displayed using Natural Earth (version 5.1.1) under a CC0 licence, French departments were displayed using ADMIN EXPRESS database from the National Institute of Geography (IGN), freely available under a CC BY licence.

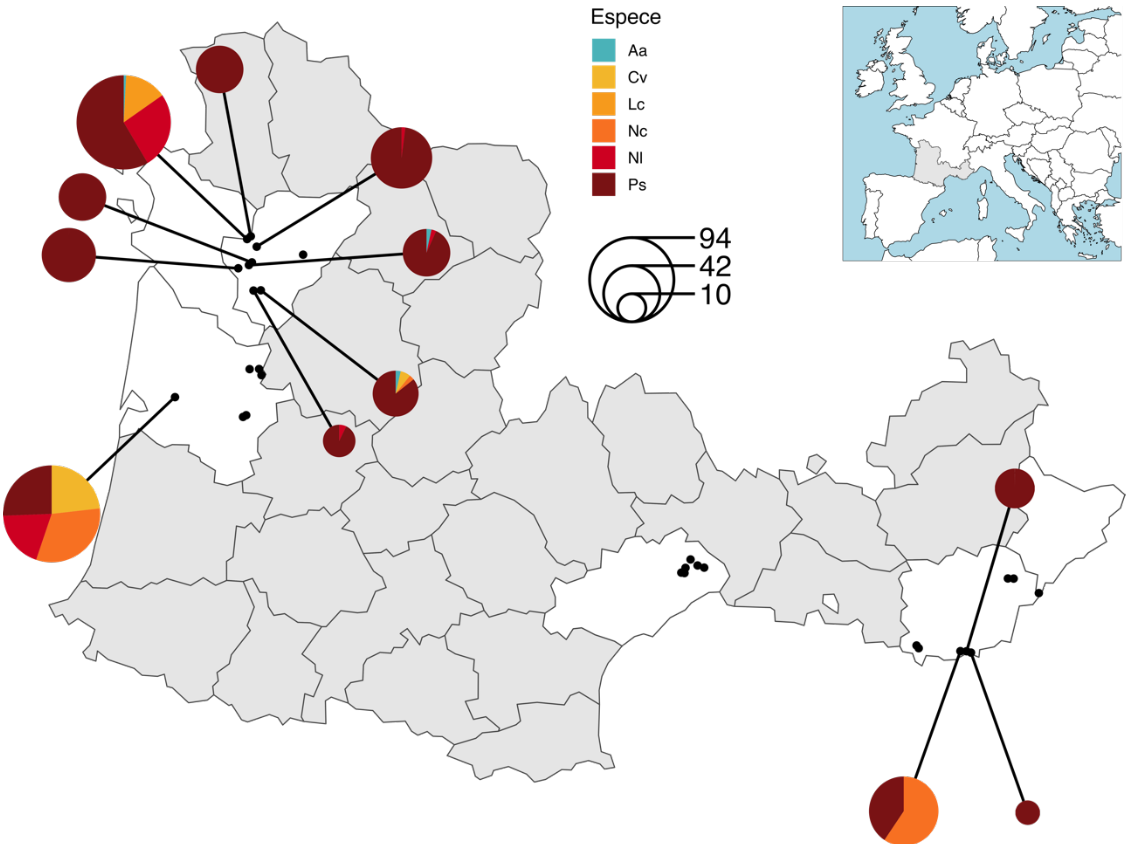

Figure S1.4. Distribution of insects sampled per buffer in the fall 2020 and screened in molecular biology for the presence of *Xf*. Insect species are abbreviated as follows Aa: *Aphrophora alni*, Agrs: *Aphrophora* grp. *salicina*, Cv: *Cicadella viridis*, Lc: *Lepyronia coleoptrata*, Nc: *Neophilaenus campestris*, Nl: *Neophilaenus lineatus*, Nsp.: *Neophilaenus* sp. and Ps: *Philaenus spumarius*. Country contours were displayed using Natural Earth (version 5.1.1) under a CC0 licence, French departments were displayed using ADMIN EXPRESS database from the National Institute of Geography (IGN), freely available under a CC BY licence.

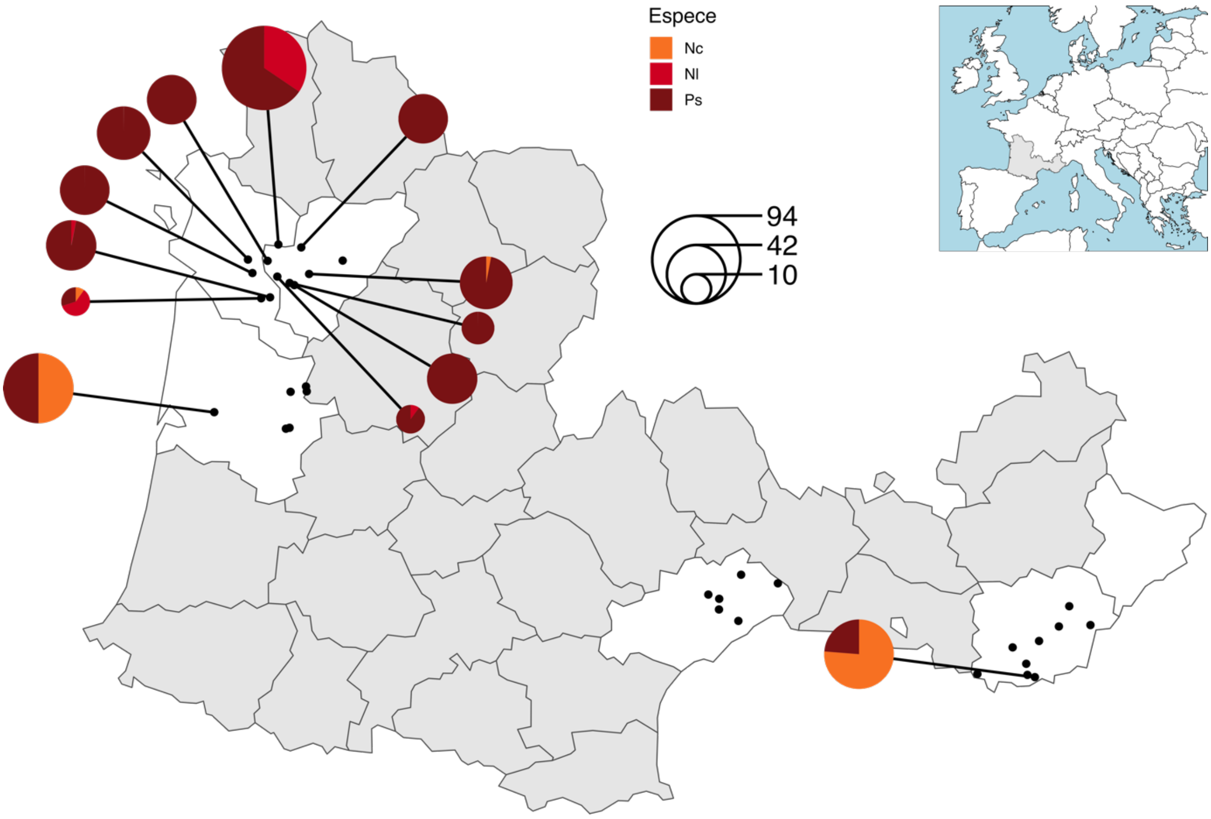

Figure S1.5. Distribution of insects sampled per buffer in the fall 2021 and screened in molecular biology for the presence of *Xf*. Insect species are abbreviated as follows Aa: *Aphrophora alni*, Agrs: *Aphrophora* grp. *salicina*, Cv: *Cicadella viridis*, Lc: *Lepyronia coleoptrata*, Nc: *Neophilaenus campestris*, Nl: *Neophilaenus lineatus*, Nsp.: *Neophilaenus* sp. and Ps: *Philaenus spumarius*. Country contours were displayed using Natural Earth (version 5.1.1) under a CC0 licence, French departments were displayed using ADMIN EXPRESS database from the National Institute of Geography (IGN), freely available under a CC BY licence.

Table S1.1. Distribution of the insects screened in molecular biology for the presence of *Xf* per habitat and insect species.

Session	Region	Land cover type	Stratum	Aa	Cv	Lc	Nc	Nl	Ps
Fall_2020	NAQ	Border	Lower	0	0	2	1	3	149
		Border	Upper	1	0	0	0	0	0
		Forest	Lower	0	13	0	8	6	0
		Forest	Upper	1	0	0	1	0	1
		Alfalfa	Lower	0	0	0	0	0	18
		Meadow	Lower	1	17	15	30	38	130
		Vine	Lower	0	0	0	0	0	15
		Vine	Upper	0	0	0	0	0	1
		Bonus habitat: shrubland		0	1	0	1	11	0
	PACA	Border	Lower	0	0	0	2	0	11
		Forest	Lower	0	0	0	0	0	1
		Forest	Upper	0	0	0	0	0	1
		Alfalfa	Lower	0	0	0	0	0	2
		Meadow	Lower	0	0	0	30	0	20
		Riparian	Lower	0	0	0	0	0	13
		Vine	Lower	0	0	0	5	0	7
		Vine	Upper	0	0	0	1	0	0
Fall_2021	NAQ	Border	Lower	0	0	0	2	38	79
		Alfalfa	Lower	0	0	0	0	0	90
		Meadow	Lower	0	0	0	30	0	150
		Vine	Lower	0	0	0	0	0	12
	PACA	Border	Lower	0	0	0	44	0	13
		Vine	Lower	0	0	0	1	0	1
Total				3	31	17	156	96	714

Insect species are abbreviated as follows Aa: *Aphrophora alni*, Agrs: *Aphrophora* grp. *salicina*, Cv: *Cicadella viridis*, Lc: *Lepyronia coleoptrata*, Nc: *Neophilaenus campestris*, Nl: *Neophilaenus lineatus*, Nsp.: *Neophilaenus* sp. and Ps: *Philaenus spumarius*.

Table S1.2. Number of xylem feeders sampled each session, all sites combined.

Species	Fall_2020	Spring_2021	Fall_2021	Total
Ps	1431	1034	2215	4680
Nc	420	*NA*	1759	3407
Nl	322	*NA*	359	
Nsp.	*NA*	547	*NA*	
Cv	81	0	364	445
Lc	45	16	20	81
Aa	6	19	5	30
Agrs	0	129	0	129
Total	2305	1745	4722	8772

Insect species are abbreviated as follows Aa: *Aphrophora alni*, Agrs: *Aphrophora* grp. *salicina*, Cv: *Cicadella viridis*, Lc: *Lepyronia coleoptrata*, Nc: *Neophilaenus campestris*, Nl: *Neophilaenus lineatus*, Nsp.: *Neophilaenus* sp. and Ps: *Philaenus spumarius*. At the nymph stage (Spring_2021 session) *N. campestris* and *N. lineatus* were found morphologically indistinguishable, so nymphs were analyzed at the genus level (*Neophilaenus* sp.). As *C. viridis* produces no spittle at the nymph stage, no *C. viridis* were collected in Spring.
