# Supplementary material for: Insect-habitat-plant interaction networks provide guidelines to mitigate the risk of transmission of Xylella fastidiosa to grapevine in Southern France
Source: PLoS One. 2025 Sep 15;20(9):e0332344. doi: 10.1371/journal.pone.0332344 (PMC12435670; doi:10.1371/journal.pone.0332344)
Supplement: S1 Appendix — (ZIP) [file pone.0332344.s001.zip › S2_Appendix.pdf]

Appendix S2: Phenological survey of Aphrophoridae conducted in Southern and Western France

This phenological study was conducted beginning in January 2021 to identify the peaks of nymph and adult densities. The same sampling protocol as described in the main text was used here, i.e. 4 minutes of sweep netting to sample adults, and 0.25 m^2 quadrats to sample nymphs. We visited 2 sites per sampling session in NAQ region and 4-5 sites per sampling session in OCC region. The aim of this survey was to finely monitor changes in the abundances of aphrophorids in time. We were not interested in the variations of that phenology within a given region. So we monitored repeatedly the same sites thoughout the study. This limited the extra-variability linked with site changes, but also limited the generalization potential of this phenological survey (i.e. observed dynamics are valid only locally). For adult sampling, individuals were caught and counted directly in the field, and then freed in the same site, in order not to deplete the local populations. This could not be done for nymphs, that were brought back to the lab for further identification. This might have lowered emerging adult densities, although the quadrat sizes were very small regarding the global extent of the plots sampled.

The sampling plots used for the phenological study were chosen in two contrasting climatic sites of the main sampling design: NAQ and OCC regions (see Fig 1 in main text). The sites were close to our main sampling sites (but several hundreds meters away to avoid population disturbance). For NAQ region the sites sampled here were near the westernmost site displayed on Fig 1, in the French department of Gironde. For OCC region the sites sampled here were in the middle of the sites displayed on Fig 1 for OCC region, in the French department of Hérault.

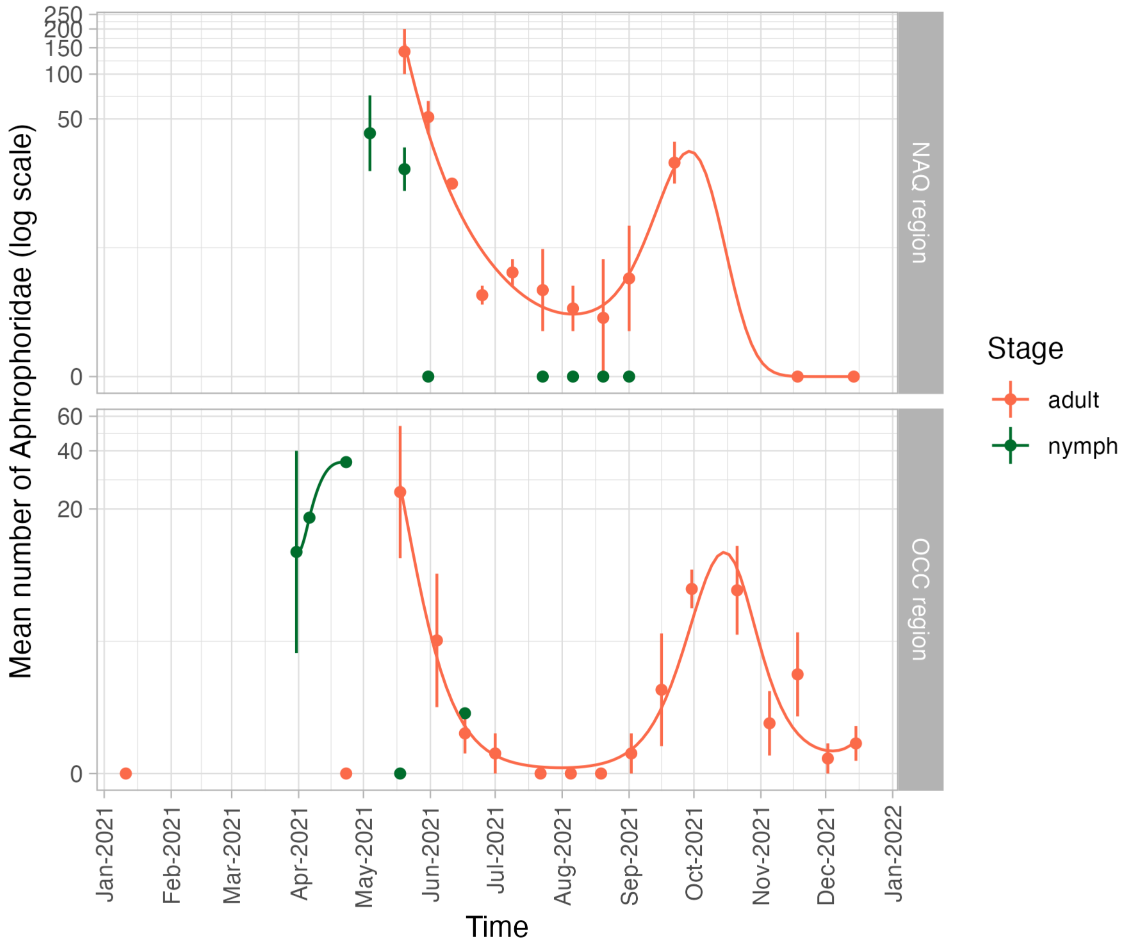

Figure S2.1 – Mean number (± standard error) of Aphrophoridae sampled by site in NAQ and OCC region in 2021 (sum of *Philaenus spumarius*, *Neophilaenus campestris, N. lineatus, N. sp., Aphrophora alni* and *Lepyronia coleoptrata*). Here, only samples made on herbaceous vegetation were considered to avoid biases due to the variable number of samples taken on upper (trees, vines) vegetation – almost always equal to 0. Regression lines were made using 'geom_smooth' function (package 'ggplot2'), using a 'glm – poisson' smooth and a polynomial fit on time (degree = 5, chosen based on the visual fit on data points).

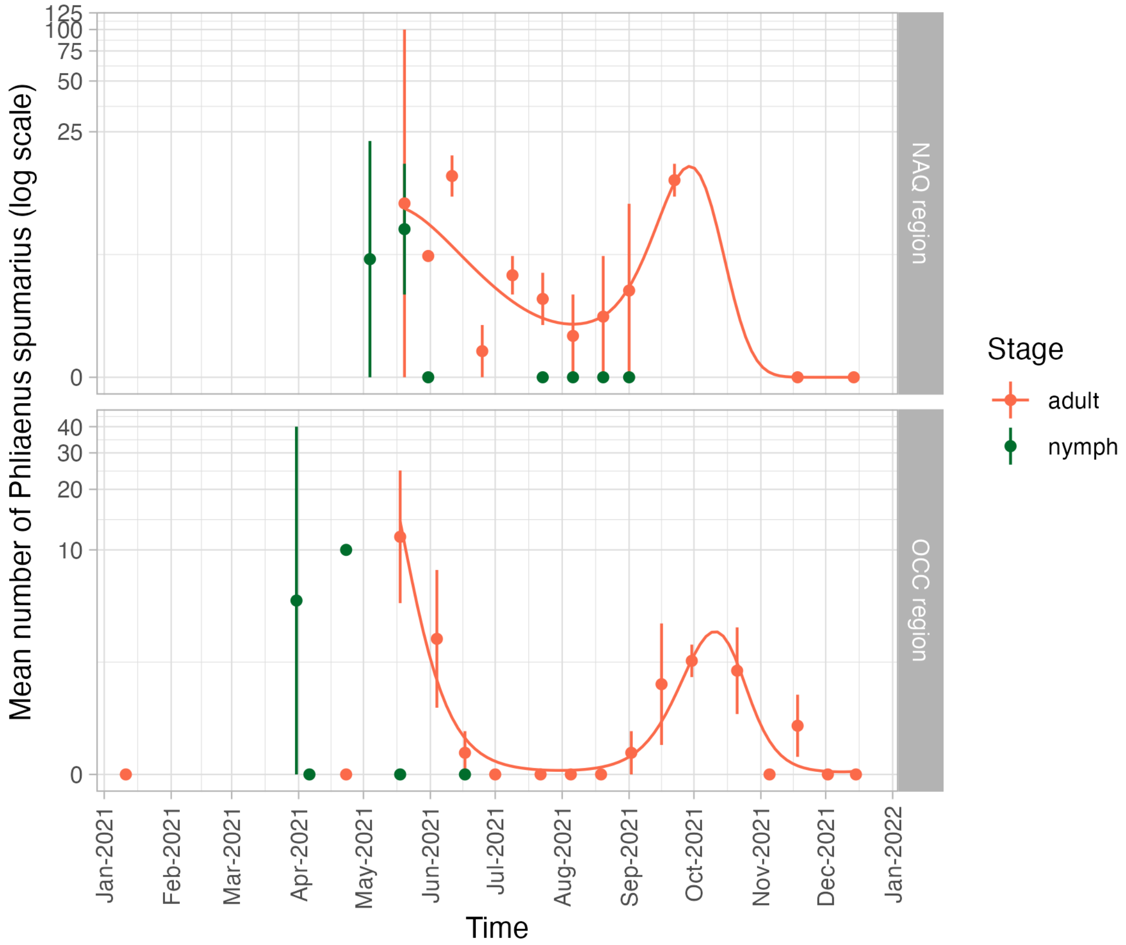

Figure S2.2 – Mean number (± standard error) of *Philaenus spumarius* sampled by site in NAQ and OCC region in 2021. Here, only samples made on herbaceous vegetation were considered to avoid biases due to the variable number of samples taken on upper (trees, vines) vegetation – almost always equal to 0. We visited 2 sites per sampling session in NAQ region and 4-5 sites per sampling session in OCC region. Regression lines were made using ‘geom_smooth’ function (package ‘ggplot2’), using a ‘glm – poisson’ smooth and a polynomial fit on time (degree = 5, chosen based on the visual fit on data points).

Table S2.1 – Sum of Aphrophoridae species (*Philaenus spumarius*, *Neophilaenus campestris, N. lineatus, N. sp., Aphrophora alni* and *Lepyronia coleoptrata*), and detailed numbers for *P. spumarius* counted during the phenological survey from 11/01/2021 to 24/05/2022, in 2 contrasted regions of the sampling design: NAQ and OCC regions. We used the same sampling protocol as explained in the Materials and Methods section of the manuscript. The of vegetation and land cover is provided in Ecosystem and Vegetation columns.

Day	Department	Lon	Lat	Ecosystem	Vegetation	Stage	Sum of Aphrophoridae	Including *Philaenus spumarius*
11/01/2021	Herault	43,72	3,86	vineyard	herbaceous vegetation	adult	0	0
11/01/2021	Herault	43,71	3,85	riparian vegetation	lower and upper vegetation	adult	0	0
11/01/2021	Herault	43,71	3,86	meadow	herbaceous vegetation	adult	0	0
11/01/2021	Herault	43,71	3,86	scrubland	herbaceous vegetation	adult	0	0
11/01/2021	Herault	43,71	3,86	meadow	herbaceous vegetation	adult	0	0
31/03/2021	Herault	43,64	3,85	scrubland	herbaceous vegetation	nymph	40	40
31/03/2021	Herault	43,67	3,87	meadow	herbaceous vegetation	nymph	3	0
06/04/2021	Herault	43,73	3,91	meadow	herbaceous vegetation	nymph	18	0
23/04/2021	Herault	43,71	3,86	meadow	herbaceous vegetation	adult	0	0
23/04/2021	Herault	43,71	3,86	meadow	herbaceous vegetation	nymph	35	10
04/05/2021	Gironde	44,74	-0,77	meadow	herbaceous vegetation	nymph	22	22
04/05/2021	Gironde	44,74	-0,78	meadow	herbaceous vegetation	nymph	72	0
18/05/2021	Herault	43,72	3,86	vineyard	herbaceous vegetation	adult	5	3
18/05/2021	Herault	43,72	3,86	vineyard	herbaceous vegetation	nymph	0	0
18/05/2021	Herault	43,71	3,86	meadow	herbaceous vegetation	adult	34	10
18/05/2021	Herault	43,71	3,86	meadow	herbaceous vegetation	nymph	0	0
18/05/2021	Herault	43,71	3,86	scrubland	herbaceous vegetation	adult	78	45
18/05/2021	Herault	43,71	3,86	scrubland	herbaceous vegetation	nymph	0	0
20/05/2021	Gironde	44,74	-0,77	meadow	herbaceous vegetation	adult	100	100
20/05/2021	Gironde	44,74	-0,77	meadow	herbaceous vegetation	nymph	16	16
20/05/2021	Gironde	44,74	-0,78	meadow	herbaceous vegetation	adult	200	0
20/05/2021	Gironde	44,74	-0,78	meadow	herbaceous vegetation	nymph	32	2
31/05/2021	Gironde	44,74	-0,77	meadow	herbaceous vegetation	adult	66	4
31/05/2021	Gironde	44,74	-0,77	meadow	herbaceous vegetation	nymph	0	0
31/05/2021	Gironde	44,74	-0,78	meadow	herbaceous vegetation	adult	40	4
31/05/2021	Gironde	44,74	-0,78	meadow	herbaceous vegetation	nymph	0	0
04/06/2021	Herault	43,72	3,86	vineyard	herbaceous vegetation	adult	0	0
04/06/2021	Herault	43,71	3,86	meadow	herbaceous vegetation	adult	8	6
04/06/2021	Herault	43,71	3,86	scrubland	herbaceous vegetation	adult	10	10
11/06/2021	Gironde	44,74	-0,77	meadow	herbaceous vegetation	adult	18	18
11/06/2021	Gironde	44,74	-0,78	meadow	herbaceous vegetation	adult	18	10
17/06/2021	Herault	43,72	3,86	vineyard	herbaceous vegetation	adult	0	0
17/06/2021	Herault	43,72	3,86	vineyard	vine foliage	adult	0	0
17/06/2021	Herault	43,71	3,85	riparian vegetation	herbaceous vegetation	nymph	1	0
17/06/2021	Herault	43,71	3,85	riparian vegetation	lower and upper vegetation	adult	1	1
17/06/2021	Herault	43,71	3,86	meadow	herbaceous vegetation	adult	1	0
17/06/2021	Herault	43,71	3,86	scrubland	herbaceous vegetation	adult	1	1
25/06/2021	Gironde	44,74	-0,77	meadow	herbaceous vegetation	adult	2	1
25/06/2021	Gironde	44,74	-0,78	meadow	herbaceous vegetation	adult	3	0
01/07/2021	Herault	43,72	3,86	vineyard	herbaceous vegetation	adult	0	0
01/07/2021	Herault	43,72	3,86	vineyard	vine foliage	adult	0	0
01/07/2021	Herault	43,71	3,85	riparian vegetation	lower and upper vegetation	adult	3	0
01/07/2021	Herault	43,71	3,86	meadow	herbaceous vegetation	adult	1	0
01/07/2021	Herault	43,71	3,86	scrubland	herbaceous vegetation	adult	0	0
09/07/2021	Gironde	44,74	-0,77	meadow	herbaceous vegetation	adult	3	2
09/07/2021	Gironde	44,74	-0,78	meadow	herbaceous vegetation	adult	5	4
22/07/2021	Herault	43,72	3,86	vineyard	herbaceous vegetation	adult	0	0
22/07/2021	Herault	43,72	3,86	vineyard	vine foliage	adult	0	0
22/07/2021	Herault	43,71	3,85	riparian vegetation	lower and upper vegetation	adult	0	0
22/07/2021	Herault	43,71	3,86	meadow	herbaceous vegetation	adult	0	0
22/07/2021	Herault	43,71	3,86	scrubland	herbaceous vegetation	adult	0	0
23/07/2021	Gironde	44,74	-0,77	meadow	herbaceous vegetation	adult	1	1
23/07/2021	Gironde	44,74	-0,77	meadow	herbaceous vegetation	nymph	0	0
23/07/2021	Gironde	44,74	-0,78	meadow	herbaceous vegetation	adult	6	3
23/07/2021	Gironde	44,74	-0,78	meadow	herbaceous vegetation	nymph	0	0
05/08/2021	Herault	43,72	3,86	vineyard	herbaceous vegetation	adult	0	0
05/08/2021	Herault	43,72	3,86	vineyard	vine foliage	adult	0	0
05/08/2021	Herault	43,71	3,85	riparian vegetation	lower and upper vegetation	adult	1	0
05/08/2021	Herault	43,71	3,86	meadow	herbaceous vegetation	adult	0	0
05/08/2021	Herault	43,71	3,86	scrubland	herbaceous vegetation	adult	0	0
06/08/2021	Gironde	44,74	-0,77	meadow	herbaceous vegetation	adult	3	2
06/08/2021	Gironde	44,74	-0,77	meadow	herbaceous vegetation	nymph	0	0
06/08/2021	Gironde	44,74	-0,78	meadow	herbaceous vegetation	adult	1	0
06/08/2021	Gironde	44,74	-0,78	meadow	herbaceous vegetation	nymph	0	0
19/08/2021	Herault	43,72	3,86	vineyard	herbaceous vegetation	adult	0	0
19/08/2021	Herault	43,72	3,86	vineyard	vine foliage	adult	0	0
19/08/2021	Herault	43,71	3,85	riparian vegetation	lower and upper vegetation	adult	4	3
19/08/2021	Herault	43,71	3,86	meadow	herbaceous vegetation	adult	0	0
19/08/2021	Herault	43,71	3,86	scrubland	herbaceous vegetation	adult	0	0
20/08/2021	Gironde	44,74	-0,77	meadow	herbaceous vegetation	adult	5	4
20/08/2021	Gironde	44,74	-0,77	meadow	herbaceous vegetation	nymph	0	0

20/08/2021	Gironde	44,74	-0,78	meadow	herbaceous vegetation	adult	0	0
20/08/2021	Gironde	44,74	-0,78	meadow	herbaceous vegetation	nymph	0	0
01/09/2021	Gironde	44,74	-0,77	meadow	herbaceous vegetation	adult	9	9
01/09/2021	Gironde	44,74	-0,77	meadow	herbaceous vegetation	nymph	0	0
01/09/2021	Gironde	44,74	-0,78	meadow	herbaceous vegetation	adult	1	0
01/09/2021	Gironde	44,74	-0,78	meadow	herbaceous vegetation	nymph	0	0
02/09/2021	Herault	43,72	3,86	vineyard	herbaceous vegetation	adult	0	0
02/09/2021	Herault	43,72	3,86	vineyard	vine foliage	adult	0	0
02/09/2021	Herault	43,71	3,85	riparian vegetation	lower and upper vegetation	adult	1	1
02/09/2021	Herault	43,71	3,86	meadow	herbaceous vegetation	adult	1	1
02/09/2021	Herault	43,71	3,86	scrubland	herbaceous vegetation	adult	0	0
16/09/2021	Herault	43,72	3,86	vineyard	herbaceous vegetation	adult	0	0
16/09/2021	Herault	43,72	3,86	vineyard	vine foliage	adult	1	1
16/09/2021	Herault	43,71	3,85	riparian vegetation	lower and upper vegetation	adult	0	0
16/09/2021	Herault	43,71	3,86	meadow	herbaceous vegetation	adult	1	1
16/09/2021	Herault	43,71	3,86	scrubland	herbaceous vegetation	adult	8	8
22/09/2021	Gironde	44,74	-0,77	meadow	herbaceous vegetation	adult	18	16
22/09/2021	Gironde	44,74	-0,78	meadow	herbaceous vegetation	adult	35	10
30/09/2021	Herault	43,72	3,86	vineyard	herbaceous vegetation	adult	9	1
30/09/2021	Herault	43,72	3,86	vineyard	vine foliage	adult	0	0
30/09/2021	Herault	43,71	3,85	riparian vegetation	lower and upper vegetation	adult	0	0
30/09/2021	Herault	43,71	3,86	meadow	herbaceous vegetation	adult	4	3
30/09/2021	Herault	43,71	3,86	scrubland	herbaceous vegetation	adult	6	3
30/09/2021	Herault	43,71	3,86	meadow	herbaceous vegetation	adult	13	3
21/10/2021	Herault	43,72	3,86	vineyard	herbaceous vegetation	adult	9	0
21/10/2021	Herault	43,72	3,86	vineyard	vine foliage	adult	0	0
21/10/2021	Herault	43,71	3,85	riparian vegetation	lower and upper vegetation	adult	0	0
21/10/2021	Herault	43,71	3,86	meadow	herbaceous vegetation	adult	1	1
21/10/2021	Herault	43,71	3,86	scrubland	herbaceous vegetation	adult	9	6
21/10/2021	Herault	43,71	3,86	meadow	herbaceous vegetation	adult	22	5
05/11/2021	Herault	43,72	3,86	vineyard	herbaceous vegetation	adult	5	0
05/11/2021	Herault	43,72	3,86	vineyard	vine foliage	adult	0	0
05/11/2021	Herault	43,71	3,85	riparian vegetation	herbaceous vegetation	adult	0	0
05/11/2021	Herault	43,71	3,85	riparian vegetation	upper vegetation	adult	0	0
05/11/2021	Herault	43,71	3,86	meadow	herbaceous vegetation	adult	0	0
05/11/2021	Herault	43,71	3,86	scrubland	herbaceous vegetation	adult	0	0
05/11/2021	Herault	43,71	3,86	meadow	herbaceous vegetation	adult	2	0
18/11/2021	Gironde	44,74	-0,77	meadow	herbaceous vegetation	adult	0	0
18/11/2021	Gironde	44,74	-0,78	meadow	herbaceous vegetation	adult	0	0
18/11/2021	Herault	43,72	3,86	vineyard	herbaceous vegetation	adult	0	0
18/11/2021	Herault	43,72	3,86	vineyard	vine foliage	adult	0	0
18/11/2021	Herault	43,71	3,85	riparian vegetation	lower and upper vegetation	adult	0	0
18/11/2021	Herault	43,71	3,86	meadow	herbaceous vegetation	adult	1	1
18/11/2021	Herault	43,71	3,86	scrubland	herbaceous vegetation	adult	7	3
18/11/2021	Herault	43,71	3,86	meadow	herbaceous vegetation	adult	5	0
02/12/2021	Herault	43,72	3,86	vineyard	herbaceous vegetation	adult	0	0
02/12/2021	Herault	43,72	3,86	vineyard	vine foliage	adult	0	0
02/12/2021	Herault	43,71	3,85	riparian vegetation	lower and upper vegetation	adult	0	0
02/12/2021	Herault	43,71	3,86	meadow	herbaceous vegetation	adult	0	0
02/12/2021	Herault	43,71	3,86	scrubland	herbaceous vegetation	adult	0	0
02/12/2021	Herault	43,71	3,86	meadow	herbaceous vegetation	adult	1	0
14/12/2021	Gironde	44,74	-0,77	meadow	herbaceous vegetation	adult	0	0
14/12/2021	Gironde	44,74	-0,78	meadow	herbaceous vegetation	adult	0	0
15/12/2021	Herault	43,72	3,86	vineyard	herbaceous vegetation	adult	0	0
15/12/2021	Herault	43,71	3,85	riparian vegetation	lower and upper vegetation	adult	0	0
15/12/2021	Herault	43,71	3,86	meadow	herbaceous vegetation	adult	1	0
15/12/2021	Herault	43,71	3,86	scrubland	herbaceous vegetation	adult	0	0
15/12/2021	Herault	43,71	3,86	meadow	herbaceous vegetation	adult	1	0
28/01/2022	Gironde	44,74	-0,77	meadow	herbaceous vegetation	adult	0	0
28/01/2022	Gironde	44,74	-0,78	meadow	herbaceous vegetation	adult	0	0
11/02/2022	Gironde	44,74	-0,77	meadow	herbaceous vegetation	adult	0	0
11/02/2022	Gironde	44,74	-0,78	meadow	herbaceous vegetation	adult	0	0
17/02/2022	Herault	43,72	3,86	vineyard	herbaceous vegetation	adult	0	0
17/02/2022	Herault	43,71	3,85	riparian vegetation	lower and upper vegetation	adult	0	0
17/02/2022	Herault	43,71	3,86	meadow	herbaceous vegetation	adult	0	0
17/02/2022	Herault	43,71	3,86	scrubland	herbaceous vegetation	adult	0	0
17/02/2022	Herault	43,71	3,86	meadow	herbaceous vegetation	adult	0	0
28/02/2022	Gironde	44,74	-0,77	meadow	herbaceous vegetation	adult	0	0
28/02/2022	Gironde	44,74	-0,78	meadow	herbaceous vegetation	adult	0	0
01/03/2022	Herault	43,72	3,86	vineyard	herbaceous vegetation	adult	1	0
01/03/2022	Herault	43,72	3,86	vineyard	herbaceous vegetation	nymph	0	0
01/03/2022	Herault	43,71	3,85	riparian vegetation	herbaceous vegetation	adult	0	0
01/03/2022	Herault	43,71	3,85	riparian vegetation	herbaceous vegetation	nymph	0	0
01/03/2022	Herault	43,71	3,86	meadow	herbaceous vegetation	adult	0	0
01/03/2022	Herault	43,71	3,86	meadow	herbaceous vegetation	nymph	0	0
01/03/2022	Herault	43,71	3,86	scrubland	herbaceous vegetation	adult	0	0
01/03/2022	Herault	43,71	3,86	scrubland	herbaceous vegetation	nymph	0	0
01/03/2022	Herault	43,71	3,86	meadow	herbaceous vegetation	adult	0	0
01/03/2022	Herault	43,71	3,86	meadow	herbaceous vegetation	nymph	0	0
18/03/2022	Herault	43,72	3,86	vineyard	herbaceous vegetation	adult	0	0
18/03/2022	Herault	43,72	3,86	vineyard	herbaceous vegetation	nymph	0	0
18/03/2022	Herault	43,71	3,85	riparian vegetation	herbaceous vegetation	adult	0	0
18/03/2022	Herault	43,71	3,85	riparian vegetation	herbaceous vegetation	nymph	0	0
18/03/2022	Herault	43,71	3,86	meadow	herbaceous vegetation	adult	0	0
18/03/2022	Herault	43,71	3,86	meadow	herbaceous vegetation	nymph	2	2
18/03/2022	Herault	43,71	3,86	scrubland	herbaceous vegetation	adult	0	0

18/03/2022	Herault	43,71	3,86	scrubland	herbaceous vegetation	nymph	1	1
18/03/2022	Herault	43,71	3,86	meadow	herbaceous vegetation	adult	0	0
18/03/2022	Herault	43,71	3,86	meadow	herbaceous vegetation	nymph	0	0
23/03/2022	Gironde	44,74	-0,77	meadow	herbaceous vegetation	adult	1	NA
23/03/2022	Gironde	44,74	-0,77	meadow	herbaceous vegetation	nymph	69	NA
23/03/2022	Gironde	44,74	-0,78	meadow	herbaceous vegetation	adult	0	0
23/03/2022	Gironde	44,74	-0,78	meadow	herbaceous vegetation	nymph	0	0
31/03/2022	Herault	43,72	3,86	vineyard	herbaceous vegetation	adult	0	0
31/03/2022	Herault	43,72	3,86	vineyard	herbaceous vegetation	nymph	2	2
31/03/2022	Herault	43,71	3,85	riparian vegetation	herbaceous vegetation	adult	0	0
31/03/2022	Herault	43,71	3,85	riparian vegetation	herbaceous vegetation	nymph	1	0
31/03/2022	Herault	43,71	3,86	meadow	herbaceous vegetation	adult	0	0
31/03/2022	Herault	43,71	3,86	meadow	herbaceous vegetation	nymph	16	15
31/03/2022	Herault	43,71	3,86	scrubland	herbaceous vegetation	adult	0	0
31/03/2022	Herault	43,71	3,86	scrubland	herbaceous vegetation	nymph	8	8
31/03/2022	Herault	43,71	3,86	meadow	herbaceous vegetation	adult	0	0
31/03/2022	Herault	43,71	3,86	meadow	herbaceous vegetation	nymph	17	17
11/04/2022	Gironde	44,74	-0,77	meadow	herbaceous vegetation	adult	1	0
11/04/2022	Gironde	44,74	-0,77	meadow	herbaceous vegetation	nymph	44	44
11/04/2022	Gironde	44,74	-0,78	meadow	herbaceous vegetation	adult	0	0
11/04/2022	Gironde	44,74	-0,78	meadow	herbaceous vegetation	nymph	2	2
13/04/2022	Herault	43,72	3,86	vineyard	herbaceous vegetation	adult	0	0
13/04/2022	Herault	43,72	3,86	vineyard	herbaceous vegetation	nymph	2	1
13/04/2022	Herault	43,71	3,85	riparian vegetation	herbaceous vegetation	adult	0	0
13/04/2022	Herault	43,71	3,85	riparian vegetation	herbaceous vegetation	nymph	0	0
13/04/2022	Herault	43,71	3,86	meadow	herbaceous vegetation	adult	0	0
13/04/2022	Herault	43,71	3,86	meadow	herbaceous vegetation	nymph	15	15
13/04/2022	Herault	43,71	3,86	scrubland	herbaceous vegetation	adult	0	0
13/04/2022	Herault	43,71	3,86	scrubland	herbaceous vegetation	nymph	19	13
13/04/2022	Herault	43,71	3,86	meadow	herbaceous vegetation	adult	0	0
13/04/2022	Herault	43,71	3,86	meadow	herbaceous vegetation	nymph	23	13
27/04/2022	Herault	43,72	3,86	vineyard	herbaceous vegetation	adult	0	0
27/04/2022	Herault	43,72	3,86	vineyard	herbaceous vegetation	nymph	0	0
27/04/2022	Herault	43,71	3,85	riparian vegetation	herbaceous vegetation	adult	0	0
27/04/2022	Herault	43,71	3,85	riparian vegetation	herbaceous vegetation	nymph	0	0
27/04/2022	Herault	43,71	3,86	meadow	herbaceous vegetation	adult	0	0
27/04/2022	Herault	43,71	3,86	meadow	herbaceous vegetation	nymph	61	61
27/04/2022	Herault	43,71	3,86	scrubland	herbaceous vegetation	adult	0	0
27/04/2022	Herault	43,71	3,86	scrubland	herbaceous vegetation	nymph	3	3
27/04/2022	Herault	43,71	3,86	meadow	herbaceous vegetation	adult	0	0
27/04/2022	Herault	43,71	3,86	meadow	herbaceous vegetation	nymph	16	13
11/05/2022	Herault	43,72	3,86	vineyard	herbaceous vegetation	adult	3	1
11/05/2022	Herault	43,72	3,86	vineyard	herbaceous vegetation	nymph	0	0
11/05/2022	Herault	43,71	3,85	riparian vegetation	herbaceous vegetation	adult	0	0
11/05/2022	Herault	43,71	3,85	riparian vegetation	herbaceous vegetation	nymph	0	0
11/05/2022	Herault	43,71	3,86	meadow	herbaceous vegetation	adult	6	5
11/05/2022	Herault	43,71	3,86	meadow	herbaceous vegetation	nymph	0	0
11/05/2022	Herault	43,71	3,86	scrubland	herbaceous vegetation	adult	9	5
11/05/2022	Herault	43,71	3,86	scrubland	herbaceous vegetation	nymph	0	0
11/05/2022	Herault	43,71	3,86	meadow	herbaceous vegetation	adult	26	11
11/05/2022	Herault	43,71	3,86	meadow	herbaceous vegetation	nymph	4	4
24/05/2022	Herault	43,72	3,86	vineyard	herbaceous vegetation	adult	0	0
24/05/2022	Herault	43,72	3,86	vineyard	herbaceous vegetation	nymph	0	0
24/05/2022	Herault	43,71	3,85	riparian vegetation	herbaceous vegetation	adult	2	0
24/05/2022	Herault	43,71	3,85	riparian vegetation	herbaceous vegetation	nymph	0	0
24/05/2022	Herault	43,71	3,86	meadow	herbaceous vegetation	adult	1	1
24/05/2022	Herault	43,71	3,86	meadow	herbaceous vegetation	nymph	0	0
24/05/2022	Herault	43,71	3,86	scrubland	herbaceous vegetation	adult	5	5
24/05/2022	Herault	43,71	3,86	scrubland	herbaceous vegetation	nymph	0	0
24/05/2022	Herault	43,71	3,86	meadow	herbaceous vegetation	adult	5	3
24/05/2022	Herault	43,71	3,86	meadow	herbaceous vegetation	nymph	0	0
