# Supplementary material for: Insect-habitat-plant interaction networks provide guidelines to mitigate the risk of transmission of Xylella fastidiosa to grapevine in Southern France
Source: PLoS One. 2025 Sep 15;20(9):e0332344. doi: 10.1371/journal.pone.0332344 (PMC12435670; doi:10.1371/journal.pone.0332344)
Supplement: S1 Appendix — (ZIP) [file pone.0332344.s001.zip › S3_Appendix.pdf]

Appendix S3: Assessment of vegetation cover on quadrats

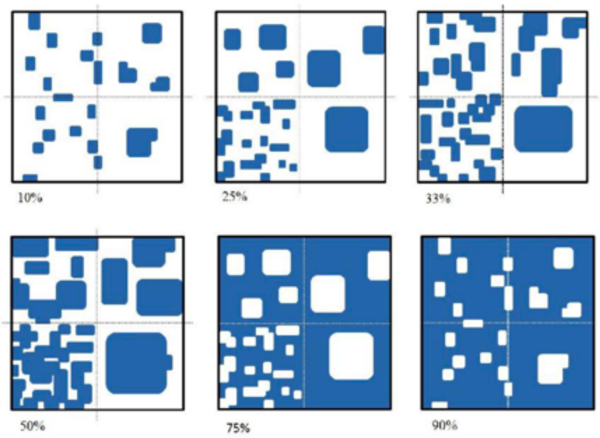

Figure S3.1. Abacus used to estimate quadrat vegetation cover

Table S3.1. Vegetation cover per land-use type (lines) and stratum (columns).

	Lower	Upper
Border	83 ± 18	74 ± 21
Forest	51 ± 30	47 ± 29
Alfalfa	90 ± 12	-
Olive grove	62 ± 27	73 ± 36
Meadow	83 ± 18	-
Riparian	68 ± 24	54 ± 25
Vine	70 ± 25	9 ± 12
All	73 ± 25	34 ± 33

Figures stand for Means ± Standard deviation. “-” stands for “stratum always unavailable”. “All” is computed on all quadrats and is not the average of table lines.
