# Supplementary material for: Insect-habitat-plant interaction networks provide guidelines to mitigate the risk of transmission of Xylella fastidiosa to grapevine in Southern France
Source: PLoS One. 2025 Sep 15;20(9):e0332344. doi: 10.1371/journal.pone.0332344 (PMC12435670; doi:10.1371/journal.pone.0332344)
Supplement: S1 Appendix — (ZIP) [file pone.0332344.s001.zip › S5_Appendix.pdf]

Appendix S5: Generalized linear mixed models details

Supplementary methods: modeling procedure

GLMM parameters were chosen to be as parsimonious as possible, while following basic modelling guidelines, checked using package 'DHARMa' [1]. Therefore, the model architecture was complexified only if the guidelines were not followed at the earlier step. All variables described in the full models were kept, since backward variable selection is considered bad statistical practice [2]. For all models we followed the steps:

- We fitted a model that followed the basic modelling guidelines with all fixed terms mentioned in the main text (see below for the parameters used depending on the type of data).
- We computed the analysis of deviance table (function 'Anova', package 'car').

Continuous data (Normalized degree, Resource Range and PDI) were analyzed using the following sequence of parameters:

- Gaussian distribution with an identity link
- If unsatisfying, Student distribution with an identity link
- If unsatisfying, Student distribution with a log link
- If unsatisfying, Tweedie distribution with a log link

Count data (raw insect abundance) were analyzed using the following sequence of parameters:

- Poisson distribution with a log link
- If unsatisfying, Negative binomial distribution linearly parameterized ("nbinom1") with a log link
- If unsatisfying, Negative binomial distribution linearly parameterized ("nbinom2") with a log link
- If unsatisfying, we compared the 12 following models and used the one that best followed DHARMa tests and that produced non-NA Anova.

Zero-inflation (using all fixed terms)	Observation-level random effect	Distribution	Link
No	No	Poisson	log
		Negative binomial linearly parameterized	
		Negative binomial quadratically parameterized	
	Yes	Poisson	
		Negative binomial linearly parameterized	
		Negative binomial quadratically parameterized	
Yes	No	Poisson	
		Negative binomial linearly parameterized	
		Negative binomial quadratically parameterized	
	Yes	Poisson	
		Negative binomial linearly parameterized	
		Negative binomial quadratically parameterized	

Table S5.1. Details of the results of the GLMM performed on habitat, plant family and plant species specialization of insects.

Question	Network used	Response variable	Network metric computed on…	Family (final model)	Link (final model)	Variable	Chi2	df	P-val	R^2_m
Insect specialization on habitat	Insect species - habitat network for each region and each session	All seven specialization metrics:<br>• ressource range<br>• paired difference index<br>• d'<br>• species specificity index<br>• z-score of ressource range<br>• z-score of paired difference index<br>• z-score of species specificity index	Present / absent and quantified interactions	Student	identity	Species	27.79	7	< 0.001	0.19
						Metric	191.40	6	< 0.001	
Insect specialization on plant family	Insect species - plant family network for each region and each session (nymph data only)			Student	log	Species	91.60	4	< 0.001	0.94
						Metric	51.01	6	< 0.001	
Insect specialization on plant species	Insect species - plant species network for each region and each session (nymph data only)			Gaussian	identity	Species	16.64	4	0.002	0.18
						Metric	27.80	6	< 0.001	

Table S5.2. Details of the results of the GLMM performed on *P. spumarius*, *Neophilaenus* sp. and *C. viridis* abundances.

Question	Network used	Response variable	Network metric computed on…	Family (final model)	Link (final model)	Variable	Chi2	df	P-val	R^2_m
Habitat preferences by the most abundant insects	Not using networks	*P. spumarius* abundance	Irrelevant	Negative binomial (nbinom1)	log	Habitat	447.24	11	< 0.001	0.72
						Region	40.01	2	< 0.001	
						Stage	6.40	1	0.011	
		Neophilaenus sp. abundance		Negative binomial (nbinom1)	log	Habitat	356.37	11	< 0.001	0.58
						Region	48.01	2	< 0.001	
						Stage	6.98	1	0.008	
		C. viridis abundance		Poisson	log	Habitat	301.39	5	< 0.001	0.39
						Region	4.87	1	0.027	

For *Cicadella viridis* abundance model, the random effect on the site ID was not included because it was never found in the upper stratum of any site, and the fixed term “Stage” was not included as it was not found at the nymph stage (no spittle produced by this species).

Table S5.3. Details of the results of the GLMM performed on xylem feeder abundance and diversity.

Question	Network used	Response variable	Network metric computed on...	Family (final model)	Link (final model)	Variable	Chi2	df	P-val	R^2_m
Habitat exploitation by insects	Not using networks	Xylem feeder abundance in each habitat		Negative binomial (nbinom1)	log	Habitat	477.20	11	< 0.001	0.63
						Region	25.56	2	< 0.001	
						Stage	23.90	1	< 0.001	
		Effective number of species (exponential of Shannon index)		Student	identity	Habitat	22.48	5	< 0.001	0.08
						Region	32.32	2	< 0.001	
						Stage	2.45	1	0.118	

For *Cicadella viridis* abundance model, the random effect on the site ID was not included because it was never found in the upper stratum of any site, and the fixed term “Stage” was not included as it was not found at the nymph stage (no spittle produced by this species).

Table S5.4. In addition to the analysis reported in Table S4.1, each specialization metric was also analyzed using GLMMs for insect-habitat networks and using Kruskal-Wallis tests [3] for insect-plant families and insect-plant species networks (insufficient number of data to use GLMMs).

Question	Network used	Response variable	Network metric computed on...	Family (final model)	Link (final model)	Variable	Chi2	df	P-val	R^2_m
Insect specialization on habitat	Insect species - habitat network for each region and each session	Ressource range	Present / absent interactions	Gaussian	identity	Species	38.72	7	< 0.001	0.03
		Paired difference index	Quantified interactions	Gaussian	identity	Species	23.33	7	0.001	0.002
		d’		Student	log	Species	265.80	7	< 0.001	0.17
		Species specificity index		Gaussian	identity	Species	51.95	7	< 0.001	0.02
		z-score of ressource range		Gaussian	identity	Species	9.10	7	0.246	0.23
		z-score of paired difference index		Gaussian	identity	Species	24.39	7	< 0.001	0.79
		z-score of species specificity index		Gaussian	identity	Species	30.50	7	< 0.001	0.98
Insect specialization on plant family	Insect species - plant family network for each region and each session (nymph data only)	Ressource range	Present / absent interactions	Kruskal-Wallis test		Species	7.24	4	0.123	-
		Paired difference index	Quantified interactions			Species	6.79	4	0.147	-
		d’				Species	4.32	4	0.364	-
		Species specificity index				Species	7.37	4	0.117	-
		z-score of ressource range				Species	7.40	4	0.116	-
		z-score of paired difference index				Species	6.91	4	0.141	-
		z-score of species specificity index				Species	7.05	4	0.133	-
Insect specialization on plant species	Insect species - plant species network for each region and each session (nymph data only)	Ressource range	Present / absent interactions	Kruskal-Wallis test		Species	8.34	4	0.080	-
		Paired difference index	Quantified interactions			Species	6.24	4	0.182	-
		d’				Species	2.92	4	0.571	-
		Species specificity index				Species	6.96	4	0.138	-
		z-score of ressource range				Species	4.73	4	0.316	-
		z-score of paired difference index				Species	3.04	4	0.552	-
		z-score of species specificity index				Species	4.87	4	0.301	-

The formula (1) in main text was simplified to keep only the “Species” fixed effect. “-“ is used for Kruskal-Wallis tests, as R^2 were not computed.

References cited in the Appendix

1. Hartig F. Package "DHARMa" Residual Diagnostics for Hierarchical (Multi-Level / Mixed) Regression Models. 2020.
2. Whittingham MJ, Stephens PA, Bradbury RB, Freckleton RP. Why do we still use stepwise modelling in ecology and behaviour? Journal of Animal Ecology. 2006;75: 1182–1189. doi:10.1111/j.1365-2656.2006.01141.x
3. Hollander M, Wolfe DA, Chicken E. Nonparametric Statistical Methods. Third Edition. Hoboken, New Jersey: John Wiley & Sons, Inc.; 2014.
