# Supplementary material for: Insect-habitat-plant interaction networks provide guidelines to mitigate the risk of transmission of Xylella fastidiosa to grapevine in Southern France
Source: PLoS One. 2025 Sep 15;20(9):e0332344. doi: 10.1371/journal.pone.0332344 (PMC12435670; doi:10.1371/journal.pone.0332344)
Supplement: S1 Appendix — (ZIP) [file pone.0332344.s001.zip › S6_Appendix.pdf]

Appendix S6: Network layout to capture the sample size of habitats using 'plotweb' function, package 'bipartite'

Consider the following random network:

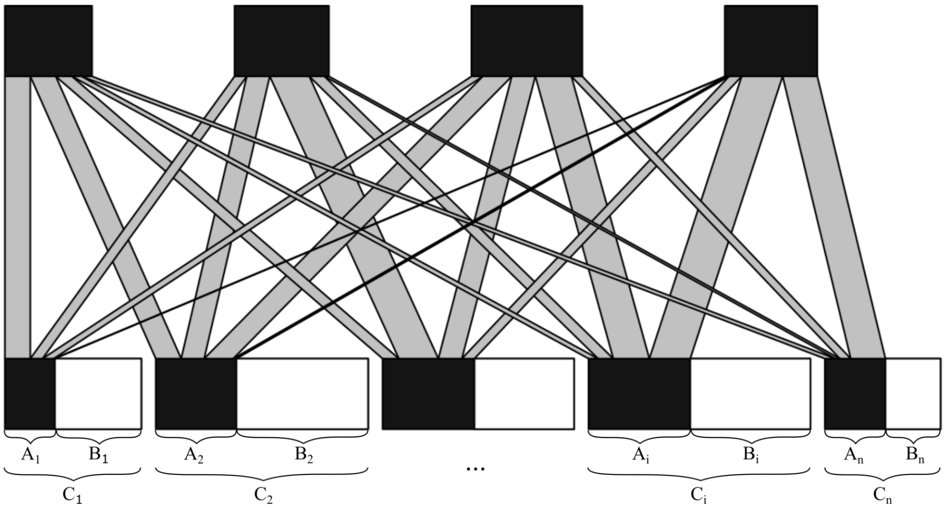

A_i is the raw number of insects sampled in habitat i. B_i is computed to include sample size information in the network based on constraints we fixed. First we define:

$$A = \sum_{i=1}^{n} A_i$$

$$B = \sum_{i=1}^{n} B_i$$

$$C = \sum_{i=1}^{n} C_i$$

For our insect-habitat networks, we wanted the "resource-level" boxes to be scaled such as:

$\frac{A_i}{C_i}$ represents the proportion of non-empty (with at least one insect) samples taken in habitat i, i.e. $\frac{A_i}{C_i} = Pfull_i = (1 - Pempty_i)$. The same holds at the scale of the whole resource level, i.e. $Pfull = \frac{A}{C}$ where $Pfull$ is the proportion, all habitats together, of non-empty (with at least one insect) samples.

$\frac{C_i}{\sum_{i=1}^{n} C_i}$ represents the proportion of subsites sampled in habitat i among the n subsites sampled, i.e. $\frac{C_i}{C} = P_i$

$B_i = C_i - A_i$

$\Leftrightarrow B_i = C_i - C_i \times Pfull_i$ [cf. constraint 1]

$\Leftrightarrow B_i = C \times P_i - C \times P_i \times Pfull_i$ [cf. constraint 2]

$\Leftrightarrow B_i = C \times P_i \times (1 - Pfull_i)$

$\Leftrightarrow B_i = C \times P_i \times Pempty_i$

$\Leftrightarrow B_i = \frac{A}{Pfull} \times P_i \times Pempty_i$ [cf. constraint 1]

A, $Pfull$, P_i, and $Pempty_i$ are all available from the data. So for each network we computed B_i and used it as the 'low.abun' argument in 'plotweb' function.
