# Supplementary material for: Insect-habitat-plant interaction networks provide guidelines to mitigate the risk of transmission of Xylella fastidiosa to grapevine in Southern France
Source: PLoS One. 2025 Sep 15;20(9):e0332344. doi: 10.1371/journal.pone.0332344 (PMC12435670; doi:10.1371/journal.pone.0332344)
Supplement: S1 Appendix — (ZIP) [file pone.0332344.s001.zip › S7_Appendix.pdf]

Appendix S7: Insect-plant species interaction networks with full details on plant species

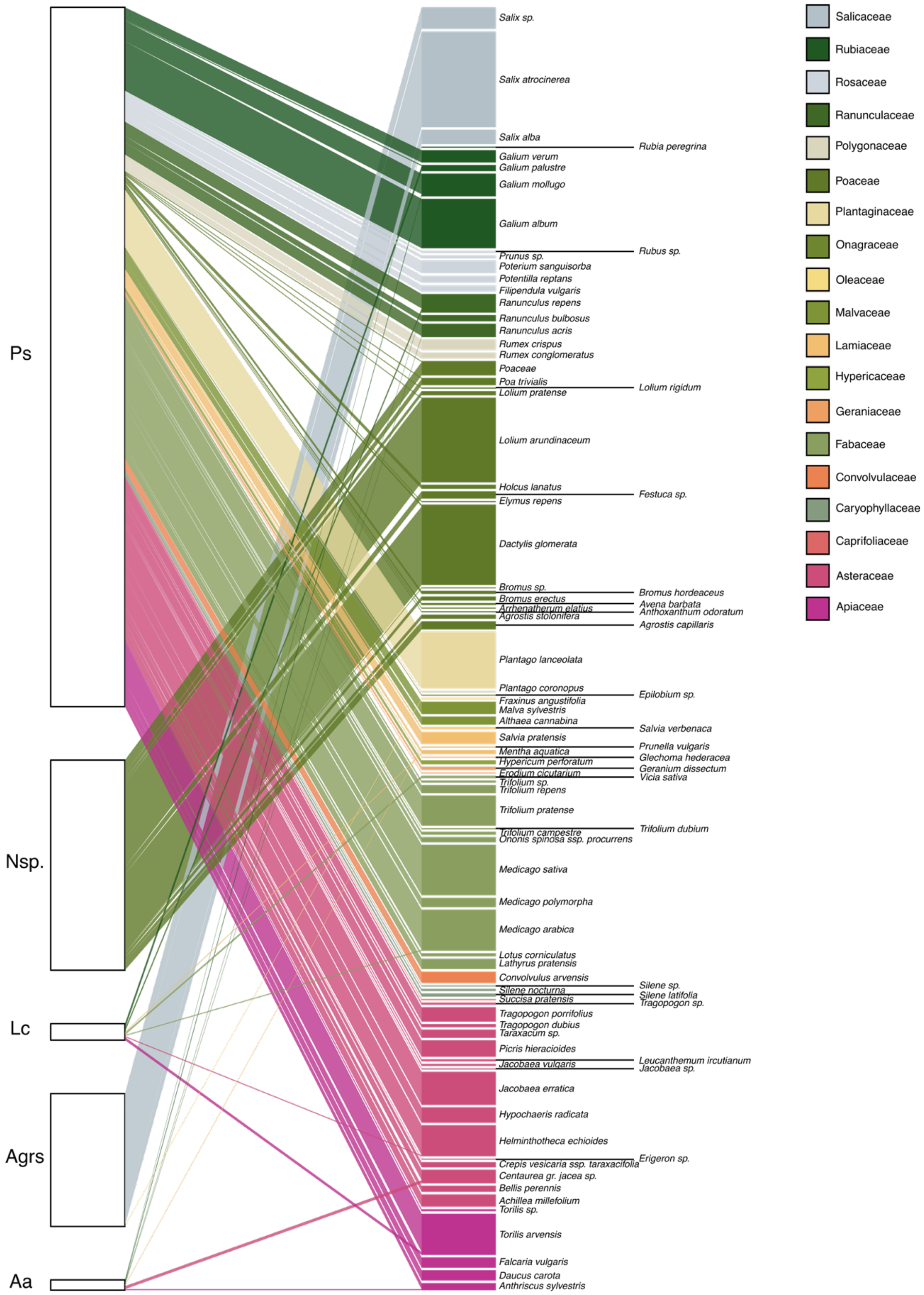

Figure S7.1. Insect plant network in NAQ region at the nymph stage (spring 2021). Insect species are abbreviated as follows Aa: *Aphrophora alni*, Agrs: *Aphrophora* grp. *salicina*, Cv: *Cicadella viridis*, Lc: *Lepyronia coleoptrata*, Nc: *Neophilaenus campestris*, Nl: *Neophilaenus lineatus*, Nsp.: *Neophilaenus* sp. and Ps: *Philaenus spumarius*.

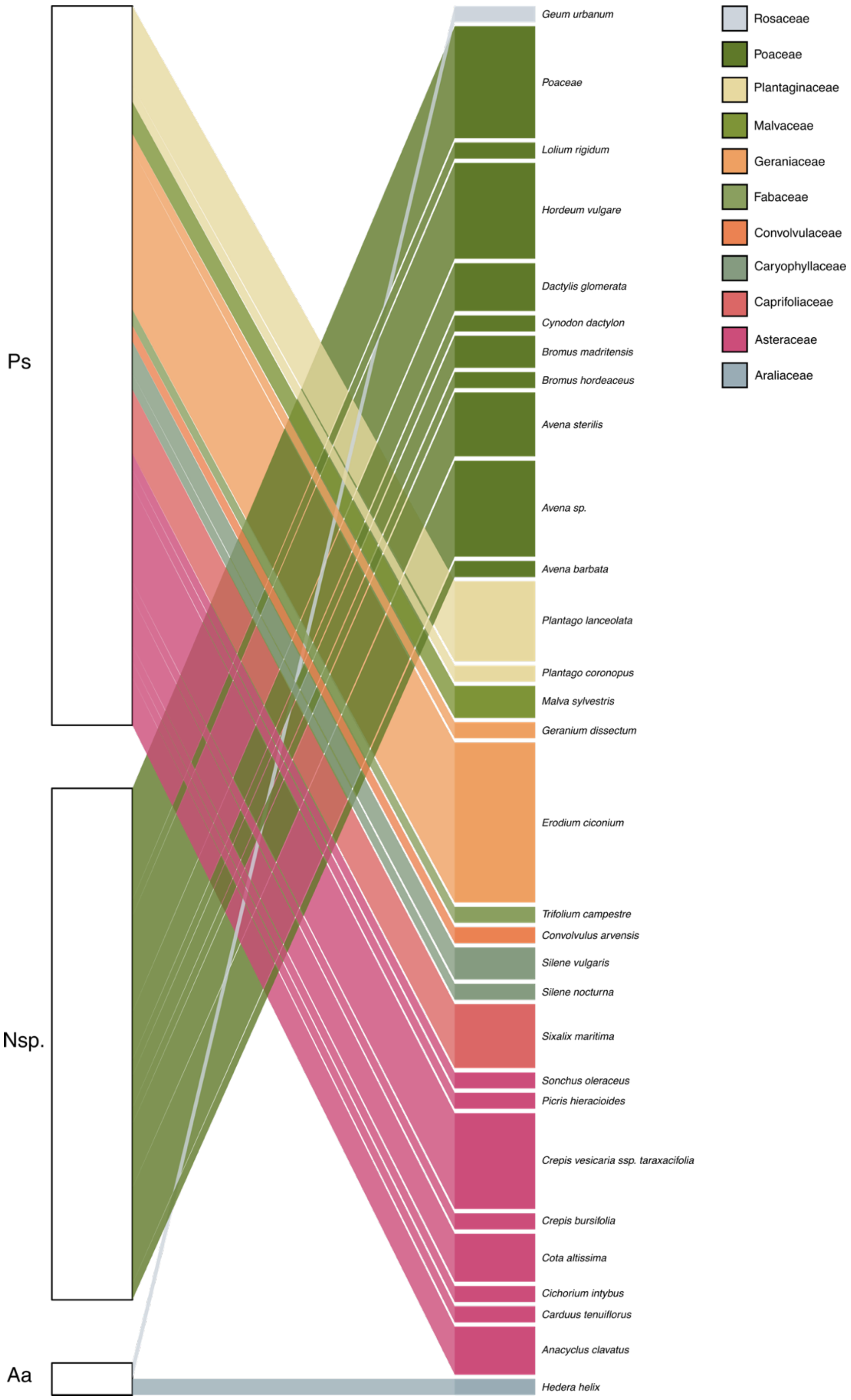

Figure S7.2. Insect plant network in OCC region at the nymph stage (spring 2021). Insect species are abbreviated as follows Aa: *Aphrophora alni*, Agrs: *Aphrophora* grp. *salicina*, Cv: *Cicadella viridis*, Lc: *Lepyronia coleoptrata*, Nc: *Neophilaenus campestris*, Nl: *Neophilaenus lineatus*, Nsp.: *Neophilaenus* sp. and Ps: *Philaenus spumarius*.

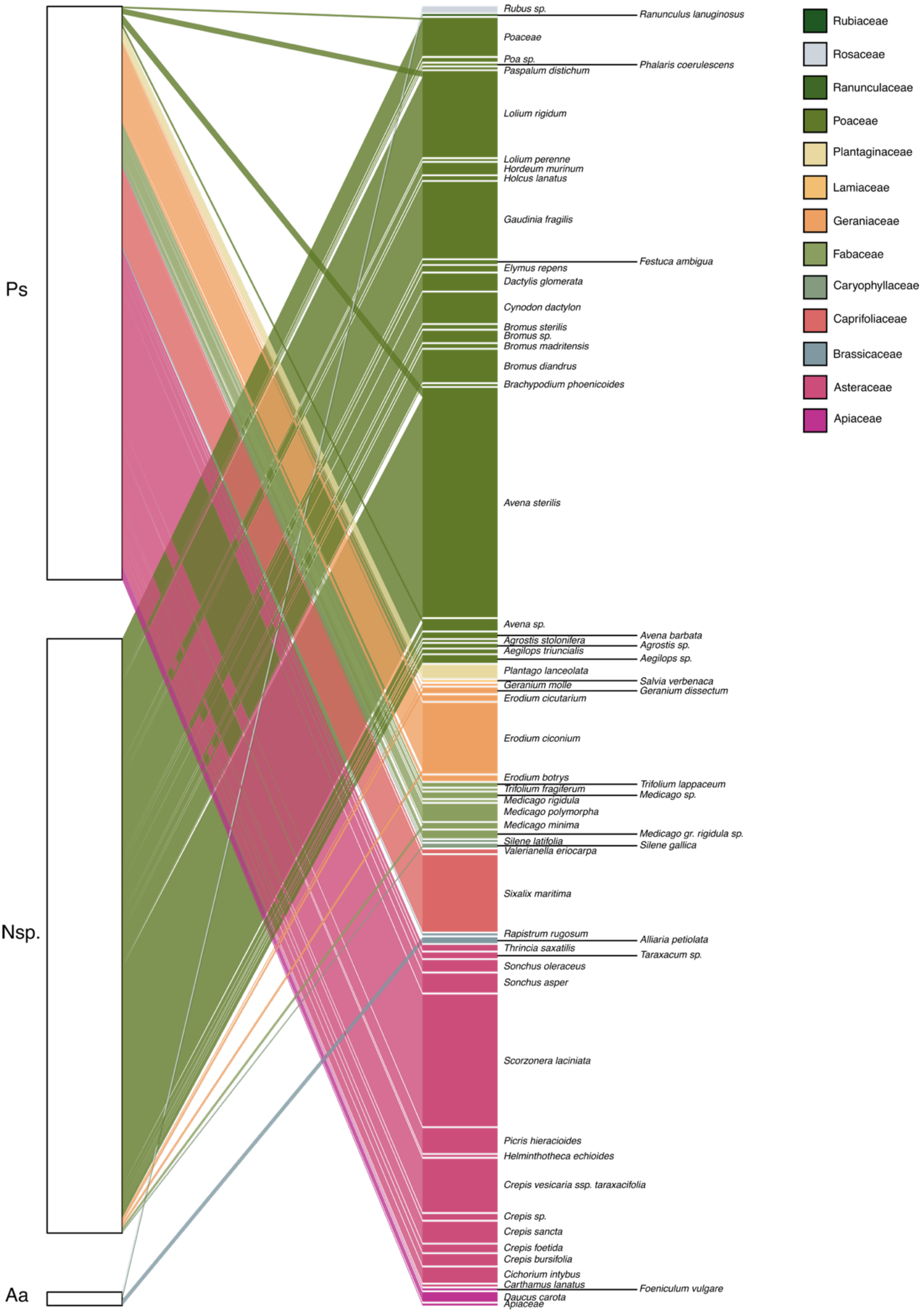

Figure S7.3. Insect plant network in PACA at the nymph stage (spring 2021). Insect species are abbreviated as follows Aa: *Aphrophora alni*, Agrs: *Aphrophora* grp. *salicina*, Cv: *Cicadella viridis*, Lc: *Lepyronia coleoptrata*, Nc: *Neophilaenus campestris*, Nl: *Neophilaenus lineatus*, Nsp.: *Neophilaenus* sp. and Ps: *Philaenus spumarius*.

Table S7.1. **Number of plant families and plant species consumed by *P. spumarius* (Ps), *Neophilaenus* sp. (Nsp.), *A. alni* (Aa), *L. coleoptrata* (Lc) and *A.* grp. *salicina* (Agrs).**

Species	#Plant family	#Plant species	Top 3 plant species
Ps	18	87	*Scorzonera laciniata; Plantago lanceolata; Medicago sativa*
Nsp.	4	31	*Avena sterilis; Dactylis glomerata; Lolium arundinaceum*
Aa	8	10	*Alliaria petiolata; Galium mollugo;* several at 1 individual
Lc	6	7	*Galium palustre; Ranunculus repens; Torilis arvensis*
Agrs	2	3	*Salix atrocinerea; Salix alba; Fraxinus angustifolia*

The first three plant species are those on which the highest number of individuals of the species was measured, all sites combined.
