# Supplementary material for: Insect-habitat-plant interaction networks provide guidelines to mitigate the risk of transmission of Xylella fastidiosa to grapevine in Southern France
Source: PLoS One. 2025 Sep 15;20(9):e0332344. doi: 10.1371/journal.pone.0332344 (PMC12435670; doi:10.1371/journal.pone.0332344)
Supplement: S1 Appendix — (ZIP) [file pone.0332344.s001.zip › S8_Appendix.pdf]

Appendix S8: Details on habitat preferences of *P. spumarius*, *Neophilaenus* sp. and *C. viridis*

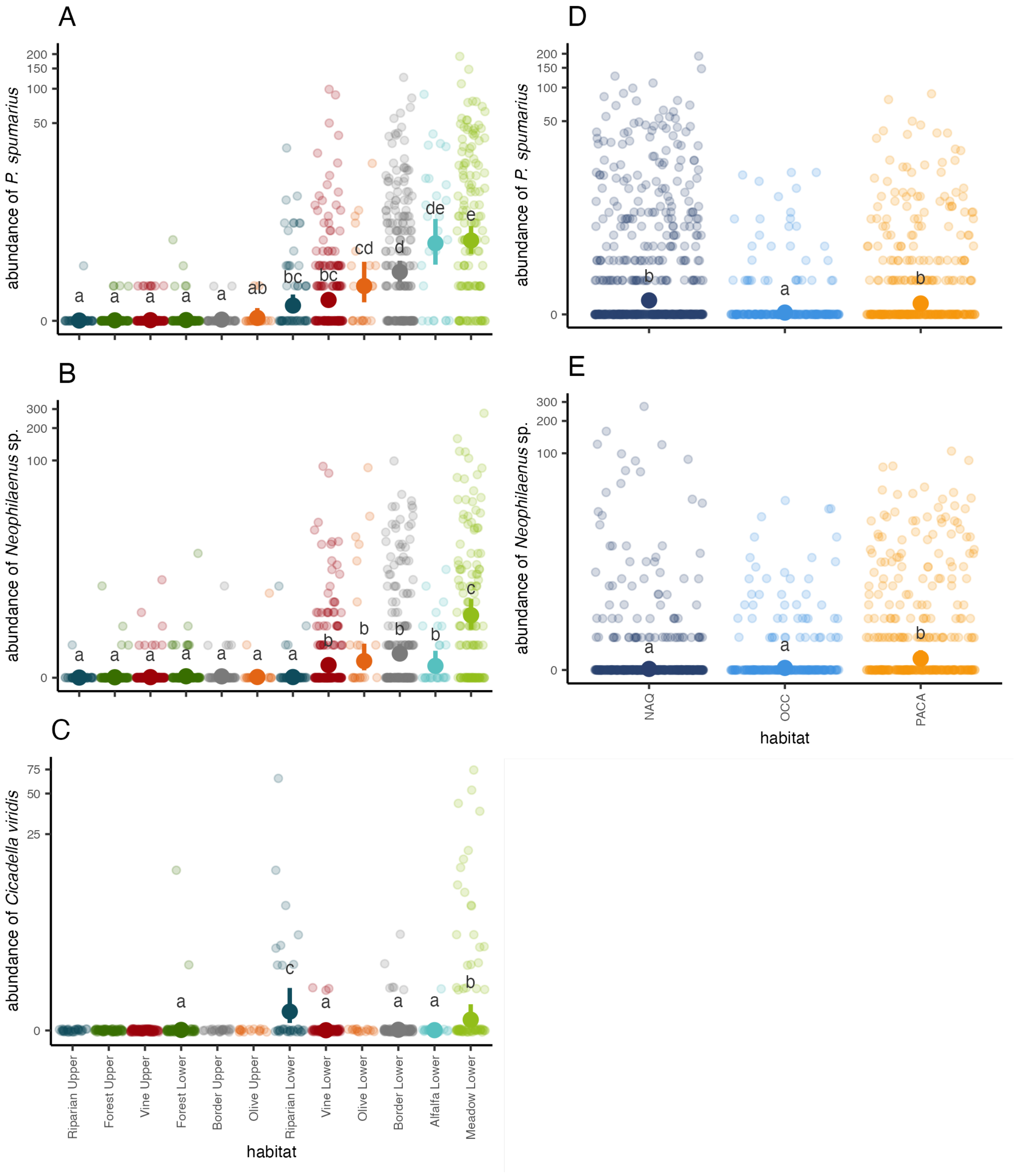

Figure S8.1. Scatterplots of raw data and estimated marginal means predicted by the model fitted on *P. spumarius* (A, D) *Neophilaenus* sp. (B, E) and *C. viridis* (C) abundance, pairwise association between habitats (A, B, C) and between regions (D, E). Abundances in habitats or regions sharing a letter do not differ significantly. Habitats that had invariably null values (for *C. viridis*) could not be compared to others in the statistical analysis (hence no letters).
