# Supplementary material for: Insect-habitat-plant interaction networks provide guidelines to mitigate the risk of transmission of Xylella fastidiosa to grapevine in Southern France
Source: PLoS One. 2025 Sep 15;20(9):e0332344. doi: 10.1371/journal.pone.0332344 (PMC12435670; doi:10.1371/journal.pone.0332344)
Supplement: S1 Appendix — (ZIP) [file pone.0332344.s001.zip › S9_Appendix.pdf]

Appendix S9: Xylem feeder abundance and diversity for each habitat and each region

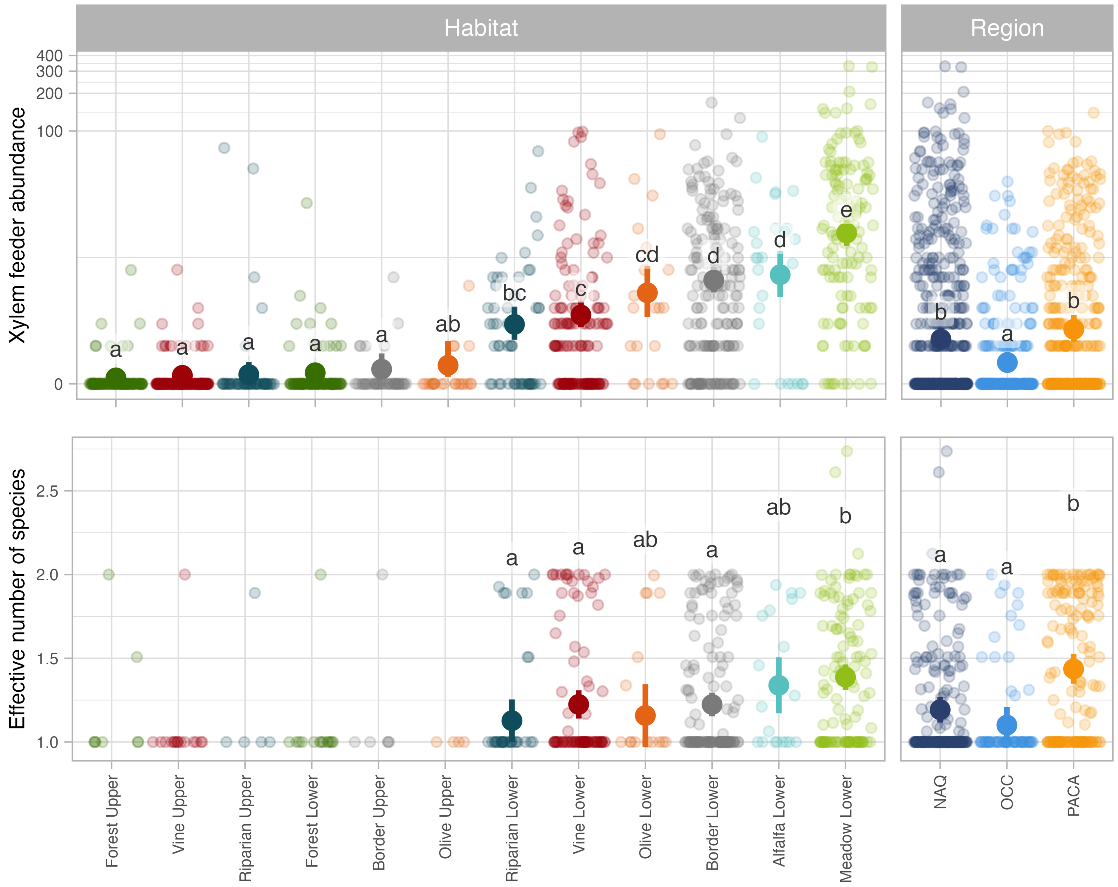

Figure S9.1. Xylem feeder abundance and diversity for each habitat and each region. (A) Abundance of xylem feeders for each habitat and each region. (B) Xylem feeder species diversity (effective number of species, exponential of Shannon index) for each habitat and each region. Letters depict the significance of the effect of habitats and regions. For each panel taken independently, modalities sharing a letter do not differ significantly.
