# Supplementary material for: Insect-habitat-plant interaction networks provide guidelines to mitigate the risk of transmission of Xylella fastidiosa to grapevine in Southern France
Source: PLoS One. 2025 Sep 15;20(9):e0332344. doi: 10.1371/journal.pone.0332344 (PMC12435670; doi:10.1371/journal.pone.0332344)
Supplement: S1 Appendix — (ZIP) [file pone.0332344.s001.zip › S10_Appendix.pdf]

Appendix S10: Details on specialization analyses

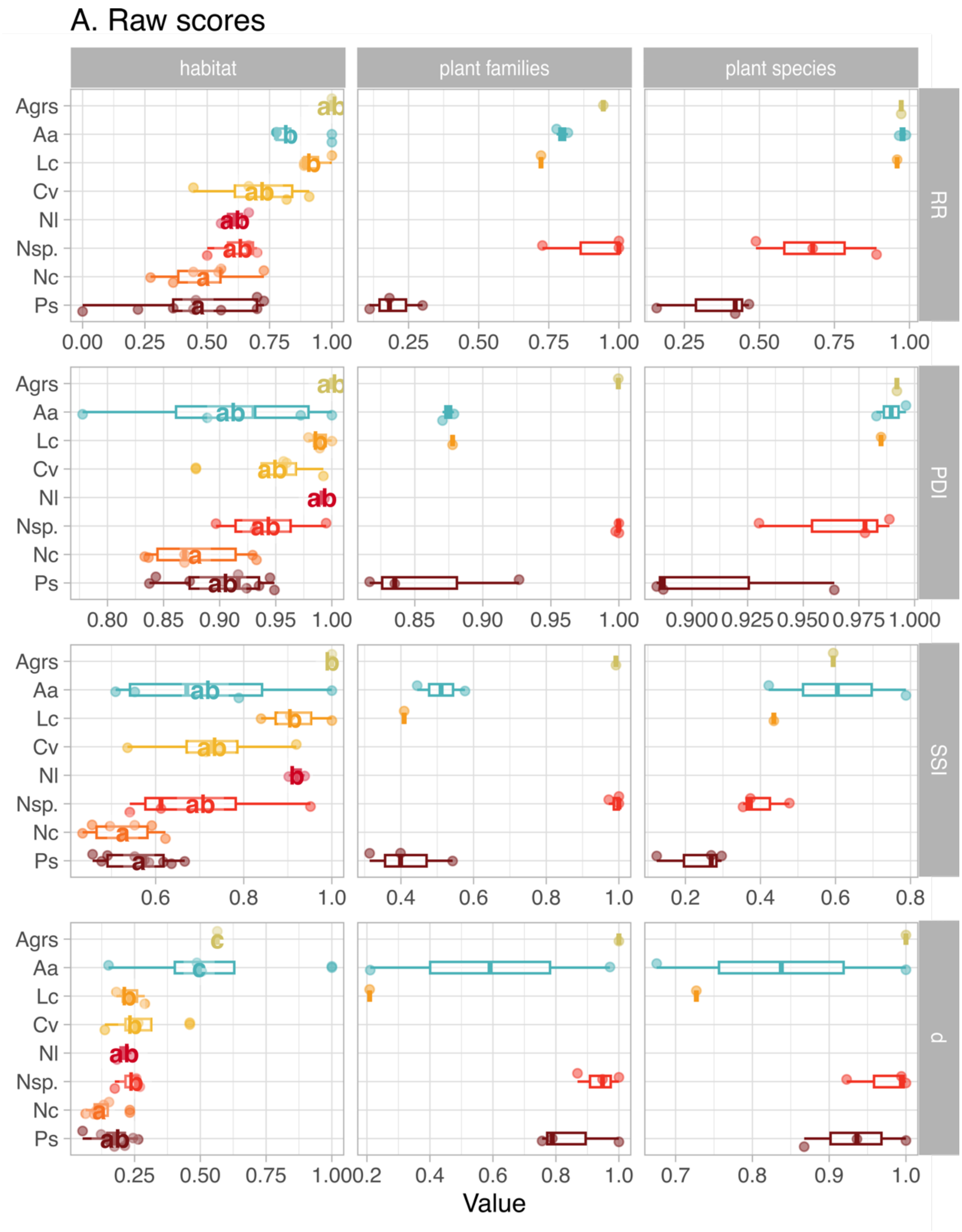

Legend below second part of the figure in next page.

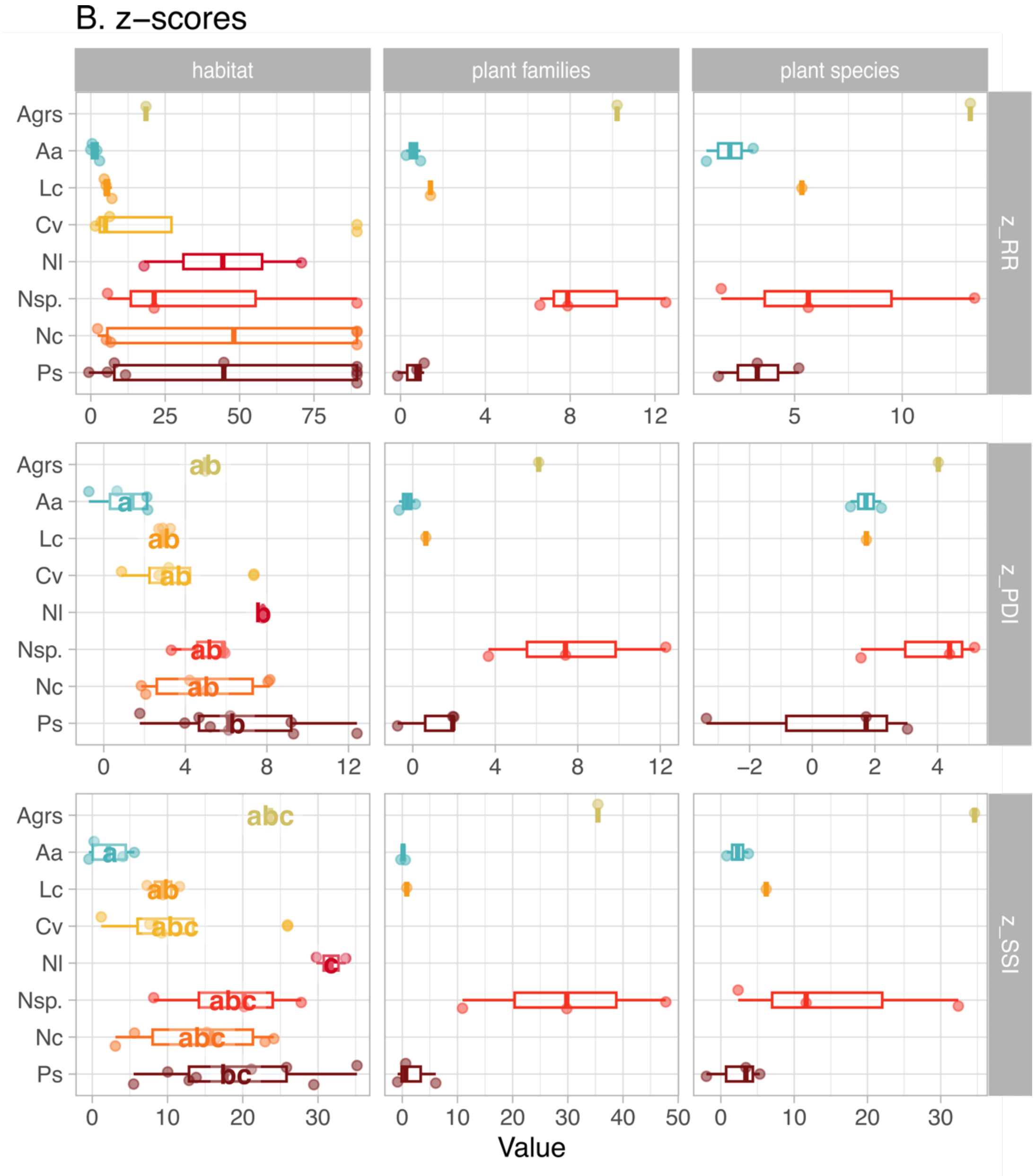

Figure S10.1. Distribution of specialization metrics for each ressource-level network, each metric, and each insect species. X scales are free, meaning that all specialization values are scaled relatively to the range of values in their panel. Fig 4 in the main text is an overlap of all panels for each column. Panels including letters indicate networks and metrics for which the fixed effect of insect species was significant. In each panel independently, species sharing a letter do not differ significantly. See Table S4.4 for details on the statistics.
