# Supplementary material for: Insect-habitat-plant interaction networks provide guidelines to mitigate the risk of transmission of Xylella fastidiosa to grapevine in Southern France
Source: PLoS One. 2025 Sep 15;20(9):e0332344. doi: 10.1371/journal.pone.0332344 (PMC12435670; doi:10.1371/journal.pone.0332344)
Supplement: S1 Appendix — (ZIP) [file pone.0332344.s001.zip › S11_Appendix.pdf]

Appendix S11: The host plants of *P. spumarius*

Table S11.1. Plant genera listed in Thompson et al. [1] and in the present study as hosts of *P. spumarius*. Taxonomy follows the currently accepted names in GBIF [2] after check with ‘get_gbif_taxonomy » function in R [3]. As several species initially described in the same genus may be distributed in different genera, we retrieved the corrected species names in Table S8.2 to perform the taxonomic revision of genera. “T23“ in the “Source“ column stands for “Already reported [1]“.

Order	Family	Genus	Author	Name in Thompson et al. (2023)	Source
Dipsacales	Caprifoliaceae	*Abelia*	R.Br.	*Linnaea*	T23
Pinales	Pinaceae	*Abies*	Mill.	*Abies*	T23
Fabales	Fabaceae	*Acacia*	Mill.	*Acacia*	T23
Asterales	Asteraceae	*Acanthospermum*	Schrank	*Acanthospermum*	T23
Lamiales	Acanthaceae	*Acanthus*	L.	*Acanthus*	T23
Sapindales	Sapindaceae	*Acer*	L.	*Acer*	T23
Asterales	Asteraceae	*Achillea*	L.	*Achillea*	T23 & this study
Apiales	Apiaceae	*Aciphylla*	J.R.Forst. & G.Forst.	*Aciphylla*	T23
Acorales	Acoraceae	*Acorus*	L.	*Acorus*	T23
Proteales	Proteaceae	*Adenanthos*	Labill.	*Adenanthos*	T23
Asterales	Asteraceae	*Adenostyles*	Cass.	*Adenostyles*	T23
Ranunculales	Ranunculaceae	*Adonis*	L.	*Adonis*	T23
Apiales	Apiaceae	*Aegopodium*	L.	*Aegopodium*	T23
Sapindales	Sapindaceae	*Aesculus*	L.	*Aesculus*	T23
Lamiales	Lamiaceae	*Agastache*	Clayton ex Gronov.	*Agastache*	T23
Rosales	Rosaceae	*Agrimonia*	L.	*Agrimonia*	T23
Caryophyllales	Caryophyllaceae	*Agrostemma*	L.	*Agrostemma*	T23
Poales	Poaceae	*Agrostis*	L.	*Agrostis*	T23
Lamiales	Lamiaceae	*Ajuga*	L.	*Ajuga*	T23
Malvales	Malvaceae	*Alcea*	L.	*Alcea*	T23
Rosales	Rosaceae	*Alchemilla*	L.	*Alchemilla*	T23
Alismatales	Alismataceae	*Alisma*	L.	*Alisma*	T23
Solanales	Solanaceae	*Alkekengi*	Mill.	*Physalis*	T23
Brassicales	Brassicaceae	*Alliaria*	Heist. ex Fabr.	*Alliaria*	T23
Asparagales	Amaryllidaceae	*Allium*	L.	*Allium*	T23
Fagales	Betulaceae	*Alnus*	Mill.	*Alnus*	T23
Asparagales	Asphodelaceae	*Aloe*	L.	*Aloe*	T23
Poales	Poaceae	*Alopecurus*	L.	*Alopecurus*	T23
Poales	Poaceae	*Alopecurus*	L.	*Alopercurus*	T23
Lamiales	Verbenaceae	*Aloysia*	Paláu	*Aloysia*	T23
Liliales	Alstroemeriaceae	*Alstroemeria*	L.	*Alstroemeria*	T23
Caryophyllales	Amaranthaceae	*Alternanthera*	Forssk.	*Alternanthera*	T23
Malvales	Malvaceae	*Althaea*	L.	*Althaea*	T23 & this study
Brassicales	Brassicaceae	*Alyssum*	L.	*Alyssum*	T23
Gentianales	Apocynaceae	*Alyxia*	Banks ex R.Br.	*Alyxia*	T23
Caryophyllales	Amaranthaceae	*Amaranthus*	L.	*Amaranthus*	T23
Asterales	Asteraceae	*Ambrosia*	L.	*Ambrosia*	T23
Rosales	Rosaceae	*Amelanchier*	Medik.	*Amelanchier*	T23
Apiales	Apiaceae	*Ammi*	L.	*Ammi*	T23
Boraginales	Boraginaceae	*Amsinckia*	Lehm.	*Amsinckia*	T23
Asterales	Asteraceae	*Anacyclus*	L.	*NA*	This study
Asterales	Asteraceae	*Anaphalis*	DC.	*Anaphalis*	T23
Boraginales	Boraginaceae	*Anchusa*	L.	*Anchusa*	T23
Ericales	Ericaceae	*Andromeda*	L.	*Andromeda*	T23
Asterales	Asteraceae	*Andryala*	L.	*Andryala*	T23
Poales	Poaceae	*Anemanthele*	Veldkamp	*Anemanthele*	T23
Ranunculales	Ranunculaceae	*Anemone*	L.	*Anemonoides*	T23
Apiales	Apiaceae	*Anethum*	L.	*Anethum*	T23
Apiales	Apiaceae	*Angelica*	L.	*Angelica*	T23
Asterales	Asteraceae	*Antennaria*	Gaertn.	*Antennaria*	T23
Asterales	Asteraceae	*Anthemis*	L.	*Anthemis*	T23
Poales	Poaceae	*Anthoxanthum*	L.	*Anthoxanthum*	T23 & this

Order	Family	Genus	Author	Name in Thompson et al. (2023)	Source
					study
Apiales	Apiaceae	*Anthriscus*	Pers.	*Anthriscus*	T23 & this study
Fabales	Fabaceae	*Anthyllis*	L.	*Anthyllis*	T23
Lamiales	Plantaginaceae	*Antirrhinum*	L.	*Antirrhinum*	T23
Poales	Poaceae	*Apera*	Adans.	*Apera*	T23
Apiales	Apiaceae	*Apium*	L.	*Apium*	T23
Ranunculales	Ranunculaceae	*Aquilegia*	L.	*Aquilegia*	T23
Brassicales	Brassicaceae	*Arabis*	L.	*Arabis*	T23
Fabales	Fabaceae	*Arachis*	L.	*Arachis*	T23
Ericales	Ericaceae	*Arbutus*	L.	*Arbutus*	T23
Asterales	Asteraceae	*Arctium*	L.	*Arctium*	T23
Asterales	Asteraceae	*Arctotheca*	J.C.Wendl.	*Arctotheca*	T23
Rosales	Rosaceae	*Argentina*	Hill	*Argentina*	T23
Rosales	Rosaceae	*Argentina*	Hill	*Potentilla*	T23
Asterales	Asteraceae	*Argyranthemum*	Webb ex Sch.Bip.	*Argyranthemum*	T23
Fabales	Fabaceae	*Argyrolobium*	Eckl. & Zeyh.	*Argyrolobium*	T23
Caryophyllales	Plumbaginaceae	*Armeria*	Willd.	*Armeria*	T23
Brassicales	Brassicaceae	*Armoracia*	G.Gaertn., B.Mey. & Scherb.	*Armoracia*	T23
Asterales	Asteraceae	*Arnica*	L.	*Arnica*	T23
Rosales	Rosaceae	*Aronia*	Medik.	*Aronia*	T23
Poales	Poaceae	*Arrhenatherum*	P.Beauv.	*Arrhenatherum*	T23 & this study
Asterales	Asteraceae	*Artemisia*	L.	*Artemisia*	T23
Alismatales	Araceae	*Arum*	L.	*Arum*	T23
Rosales	Rosaceae	*Aruncus*	Adans.	*Aruncus*	T23
Poales	Poaceae	*Arundo*	L.	*Arundo*	T23
Gentianales	Apocynaceae	*Asclepias*	L.	*Asclepias*	T23
Asparagales	Asparagaceae	*Asparagus*	Tourn. ex L.	*Asparagus*	T23
Boraginales	Boraginaceae	*Asperugo*	L.	*Asperugo*	T23
Asparagales	Asphodelaceae	*Asphodelus*	L.	*Asphodelus*	T23
Asterales	Asteraceae	*Aster*	L.	*Aster*	T23
Asterales	Asteraceae	*Asteriscus*	Mill.	*Asteriscus*	T23
Fabales	Fabaceae	*Astragalus*	L.	*Astragalus*	T23
Apiales	Apiaceae	*Astrantia*	L.	*Astrantia*	T23
Polypodiales	Athyriaceae	*Athyrium*	Roth	*Athyrium*	T23
Caryophyllales	Amaranthaceae	*Atriplex*	L.	*Atriplex*	T23
Brassicales	Brassicaceae	*Aubrieta*	Adans.	*Aubrieta*	T23
Poales	Poaceae	*Avena*	L.	*Avena*	T23 & this study
Asterales	Asteraceae	*Baccharis*	L.	*Baccharis*	T23
Asterales	Asteraceae	*Bahia*	Lag.	*Bahia*	T23
Fabales	Fabaceae	*Baptisia*	Vent.	*Baptisia*	T23
Brassicales	Brassicaceae	*Barbarea*	W.T.Aiton	*Barbarea*	T23
Lamiales	Orobanchaceae	*Bellardia*	All.	*Bellardia*	T23
Asterales	Asteraceae	*Bellis*	L.	*Bellis*	T23 & this study
Ranunculales	Berberidaceae	*Berberis*	L.	*Berberis*	T23
Saxifragales	Saxifragaceae	*Bergenia*	Moench	*Bergenia*	T23
Caryophyllales	Amaranthaceae	*Beta*	L.	*Beta*	T23
Fagales	Betulaceae	*Betula*	L.	*Betula*	T23
Asterales	Asteraceae	*Bidens*	L.	*Bidens*	T23
Apiales	Pittosporaceae	*Billardiera*	Sm.	*Billardiera*	T23
Caryophyllales	Polygonaceae	*Bistorta*	(L.) Scop.	*Bistorta*	T23
Fabales	Fabaceae	*Bituminaria*	Heist. ex Fabr.	*Psorolea*	T23
Fabales	Fabaceae	*Bituminaria*	Heist. ex Fabr.	*Bituminaria*	T23
Boraginales	Boraginaceae	*Borago*	L.	*Borago*	T23
Asterales	Asteraceae	*Brachyglottis*	J.R.Forst. & G.Forst.	*Brachyglottis*	T23
Poales	Poaceae	*Brachypodium*	P.Beauv.	*Brachypodium*	T23
Brassicales	Brassicaceae	*Brassica*	L.	*Brassica*	T23
Poales	Poaceae	*Bromus*	L.	*Bromus*	T23 & this study
Poales	Poaceae	*Bromus*	L.	*Anisantha*	T23 & this study
Lamiales	Scrophulariaceae	*Buddleja*	L.	*Buddleia*	T23
Lamiales	Scrophulariaceae	*Buddleja*	L.	*Buddleja*	T23
Asterales	Asteraceae	*Buphthalmum*	L.	*Buphthalmum*	T23

Order	Family	Genus	Author	Name in Thompson et al. (2023)	Source
Buxales	Buxaceae	*Buxus*	L.	*Buxus*	T23
Poales	Poaceae	*Calamagrostis*	Adans.	*Calamagrostis*	T23
Asterales	Asteraceae	*Calendula*	L.	*Calendula*	T23 & this study
Brassicales	Brassicaceae	*Calepina*	Adans.	*Calepina*	T23
Fabales	Fabaceae	*Calicotome*	Link	*Cytisus*	T23
Lamiales	Lamiaceae	*Callicarpa*	L.	*Callicarpa*	T23
Ericales	Ericaceae	*Calluna*	Salisb.	*Calluna*	T23
Asterales	Asteraceae	*Calocephalus*	R.Br.	*Leucophyta*	T23
Ranunculales	Ranunculaceae	*Caltha*	L.	*Caltha*	T23
Solanales	Convolvulaceae	*Calystegia*	R.Br.	*Calystegia*	T23
Asterales	Campanulaceae	*Campanula*	L.	*Campanula*	T23
Rosales	Cannabaceae	*Cannabis*	L.	*Cannabis*	T23
Brassicales	Brassicaceae	*Capsella*	Medik.	*Capsella*	T23
Solanales	Solanaceae	*Capsicum*	L.	*Capsicum*	T23
Brassicales	Brassicaceae	*Cardamine*	L.	*Cardamine*	T23
Asterales	Asteraceae	*Carduus*	L.	*Carduus*	T23 & this study
Poales	Cyperaceae	*Carex*	L.	*Carex*	T23 & this study
Asterales	Asteraceae	*Carlina*	L.	*Carlina*	T23
Fagales	Betulaceae	*Carpinus*	L.	*Carpinus*	T23
Caryophyllales	Aizoaceae	*Carpobrotus*	N.E.Br.	*Carpobrotus*	T23
Asterales	Asteraceae	*Carthamus*	L.	*NA*	This study
Apiales	Apiaceae	*Carum*	L.	*Carum*	T23
Fagales	Juglandaceae	*Carya*	Nutt.	*Carya*	T23
Fagales	Fagaceae	*Castanea*	Mill.	*Castanea*	T23
Asterales	Asteraceae	*Catananche*	L.	*Catananche*	T23
Apiales	Apiaceae	*Caucalis*	L.	*Caucalis*	T23
Rosales	Rhamnaceae	*Ceanothus*	L.	*Ceanothus*	T23
Asterales	Asteraceae	*Celmisia*	Cass.	*Celmisia*	T23
Rosales	Cannabaceae	*Celtis*	L.	*Celtis*	T23
Poales	Poaceae	*Cenchrus*	L.	*Pennisetum*	T23
Asterales	Asteraceae	*Centaurea*	L.	*Centaurea*	T23 & this study
Gentianales	Gentianaceae	*Centaurium*	Hill	*Centaurium*	T23
Dipsacales	Caprifoliaceae	*Centranthus*	Neck. ex Lam. & DC.	*Centranthus*	T23
Dipsacales	Caprifoliaceae	*Cephalaria*	Schrad.	*Cephalaria*	T23
Caryophyllales	Caryophyllaceae	*Cerastium*	L.	*Cerastium*	T23
Saxifragales	Cercidiphyllaceae	*Cercidiphyllum*	Siebold & Zucc.	*Cercidiphyllum*	T23
Apiales	Apiaceae	*Cervaria*	Wolf	*Cervaria*	T23
Solanales	Solanaceae	*Cestrum*	L.	*Cestrum*	T23
Rosales	Rosaceae	*Chaenomeles*	Lindl.	*Chaenomeles*	T23
Apiales	Apiaceae	*Chaerophyllum*	L.	*Chaerophyllum*	T23
Myrtales	Onagraceae	*Chamaenerion*	Seguier	*Chamaenerion*	T23
Ranunculales	Papaveraceae	*Chelidonium*	L.	*Chelidonium*	T23
Caryophyllales	Amaranthaceae	*Chenopodium*	L.	*Chenopodium*	T23
Asparagales	Asparagaceae	*Chlorogalum*	(Lindl.) Kunth	*Chlorogalum*	T23
Asparagales	Asparagaceae	*Chlorophytum*	Ker Gawl.	*Chlorophytum*	T23
Asterales	Asteraceae	*Chondrilla*	L.	*Chondrilla*	T23
Asterales	Asteraceae	*Chrysanthemum*	L.	*Chrysanthemum*	T23
Lamiales	Oleaceae	*Chrysojasminum*	Banfi	*Jasminum*	T23
Saxifragales	Saxifragaceae	*Chrysosplenium*	L.	*Chrysosplenium*	T23
Asterales	Asteraceae	*Cicerbita*	Wallr.	*Cicerbita*	T23
Asterales	Asteraceae	*Cichorium*	L.	*Cichorium*	T23 & this study
Myrtales	Onagraceae	*Circaea*	L.	*Circaea*	T23
Asterales	Asteraceae	*Cirsium*	Mill.	*Cirsium*	T23 & this study
Malvales	Cistaceae	*Cistus*	L.	*Cistus*	T23
Sapindales	Rutaceae	*Citrus*	L.	*Citrus*	T23
Caryophyllales	Montiaceae	*Claytonia*	L.	*Claytonia*	T23
Ranunculales	Ranunculaceae	*Clematis*	L.	*Clematis*	T23
Caryophyllales	Aizoaceae	*Cleretum*	N.E.Br.	*Cleretum*	T23
Poales	Poaceae	*Coix*	L.	*Coix*	T23
Asterales	Asteraceae	*Coleostephus*	Cass.	*Coleostephus*	T23

Order	Family	Genus	Author	Name in Thompson et al. (2023)	Source
Fabales	Fabaceae	*Colutea*	L.	*Colutea*	T23
Rosales	Rosaceae	*Comarum*	L.	*Comarum*	T23
Rosales	Rosaceae	*Comarum*	L.	*Potentilla*	T23
Commelinales	Commelinaceae	*Commelina*	Plum. ex L.	*Commelina*	T23
Fagales	Myricaceae	*Comptonia*	L'Hér. ex Aiton	*Comptonia*	T23
Apiales	Apiaceae	*Conium*	L.	*Conium*	T23
Apiales	Apiaceae	*Conopodium*	W.D.J.Koch	*Conopodium*	T23
Asparagales	Asparagaceae	*Convallaria*	L.	*Convallaria*	T23
Solanales	Convolvulaceae	*Convolvulus*	L.	*Convolvulus*	T23 & this study
Solanales	Convolvulaceae	*Convolvulus*	L.	*Convovulus*	T23 & this study
Asterales	Asteraceae	*Conyza*	Hill, 1756	*Conyza*	T23
Gentianales	Rubiaceae	*Coprosma*	J.R.Forst. & G.Forst.	*Coprosma*	T23
Asparagales	Asparagaceae	*Cordyline*	Comm. ex R.Br.	*Cordyline*	T23
Asterales	Asteraceae	*Coreopsis*	L.	*Coreopsis*	T23
Apiales	Apiaceae	*Coriandrum*	L.	*Coriandrum*	T23
Cucurbitales	Coriariaceae	*Coriaria*	L.	*Coriaria*	T23
Cornales	Cornaceae	*Cornus*	L.	*Cornus*	T23
Fabales	Fabaceae	*Coronilla*	L.	*Coronilla*	T23
Fabales	Fabaceae	*Coronilla*	L.	*Securigara*	T23
Fagales	Betulaceae	*Corylus*	L.	*Corylus*	T23
Poales	Poaceae	*Corynephorus*	P.Beauv.	*Corynephorus*	T23
Asterales	Asteraceae	*Cosmos*	Cav.	*Cosmos*	T23
Asterales	Asteraceae	*Cota*	J.Gay	*NA*	This study
Sapindales	Anacardiaceae	*Cotinus*	Mill.	*Cotinus*	T23
Rosales	Rosaceae	*Cotoneaster*	Medik.	*Cotoneaster*	T23
Saxifragales	Crassulaceae	*Crassula*	L.	*Crassula*	T23
Rosales	Rosaceae	*Crataegus*	L.	*Crataegus*	T23
Asterales	Asteraceae	*Crepis*	L.	*Crepis*	T23 & this study
Oxalidales	Elaeocarpaceae	*Crinodendron*	Molina	*Crinodendron*	T23
Apiales	Apiaceae	*Crithmum*	L.	*Crithmum*	T23
Asparagales	Iridaceae	*Crocosmia*	Planch.	*Crocosmia*	T23
Gentianales	Rubiaceae	*Crucianella*	L.	*Crucianella*	T23
Gentianales	Rubiaceae	*Cruciata*	Mill.	*Cruciata*	T23
Cucurbitales	Cucurbitaceae	*Cucumis*	L.	*Cucumis*	T23
Cucurbitales	Cucurbitaceae	*Cucurbita*	L.	*Cucurbita*	T23
Pinales	Cupressaceae	*Cupressus*	L.	*Hesperocyparis*	T23
Pinales	Cupressaceae	*Cupressus*	L.	*Cupressus*	T23
Rosales	Rosaceae	*Cydonia*	Tourn. ex Mill	*Cydonia*	T23
Asterales	Asteraceae	*Cynara*	L.	*Cynara*	T23
Poales	Poaceae	*Cynodon*	Rich.	*Cynodon*	T23
Poales	Poaceae	*Cynosurus*	L.	*Cynosurus*	T23
Poales	Poaceae	*Cynosurus*	L.	*Cyinosurus*	T23
Poales	Cyperaceae	*Cyperus*	L.	*Kyllinga*	T23
Fabales	Fabaceae	*Cytisus*	Desf.	*Cytisus*	T23
Poales	Poaceae	*Dactylis*	L.	*Dactylis*	T23 & this study
Asparagales	Orchidaceae	*Dactylorhiza*	Neck. ex Nevski	*Dactylorhiza*	T23
Asterales	Asteraceae	*Dahlia*	Cav.	*Dahlia*	T23
Malvales	Thymelaeaceae	*Daphne*	Tourn. ex L.	*Daphne*	T23
Rosales	Rosaceae	*Dasiphora*	Raf.	*Dasiphora*	T23
Poales	Poaceae	*Dasypyrum*	(Coss. & Durieu) T.Durand	*Dasypyrum*	T23
Apiales	Apiaceae	*Daucus*	L.	*Daucus*	T23 & this study
Poales	Poaceae	*Deschampsia*	P.Beauv.	*Deschampsia*	T23
Brassicales	Brassicaceae	*Descurainia*	Webb & Berthel.	*Descurainia*	T23
Caryophyllales	Caryophyllaceae	*Dianthus*	L.	*Dianthus*	T23
Poales	Poaceae	*Dichanthelium*	(Hitchc. & Chase) Gould	*Dichanthelium*	T23
Dipsacales	Caprifoliaceae	*Diervilla*	Tourn. ex Mill.	*Diervilla*	T23
Poales	Poaceae	*Digitaria*	Haller	*Digitaria*	T23
Asterales	Asteraceae	*Dimorphotheca*	Vaill.	*Dimorphotheca*	T23
Lamiales	Phrymaceae	*Diplacus*	Nutt.	*Diplacus*	T23
Brassicales	Brassicaceae	*Diplotaxis*	DC.	*Diplotaxis*	T23
Dipsacales	Caprifoliaceae	*Dipsacus*	L.	*Dipsacus*	T23
Malvales	Thymelaeaceae	*Dirca*	L.	*Dirca*	T23
Asterales	Asteraceae	*Dittrichia*	Greuter	*Dittrichia*	T23
Lamiales	Lamiaceae	*Dracocephalum*	L.	*Dracocephalum*	T23
Polypodiales	Dryopteridaceae	*Dryopteris*	Adans.	*Dryopteris*	T23
Asterales	Asteraceae	*Dubautia*	Gaudich.	*Dubautia*	T23

Order	Family	Genus	Author	Name in Thompson et al. (2023)	Source
Cucurbitales	Cucurbitaceae	*Ecballium*	A.Rich.	*Ecballium*	T23
Asterales	Asteraceae	*Echinacea*	Moench	*Echinacea*	T23
Asterales	Asteraceae	*Echinops*	L.	*Echinops*	T23
Boraginales	Boraginaceae	*Echium*	L.	*Echium*	T23
Poales	Poaceae	*Elymus*	L.	*Elymus*	T23 & this study
Ericales	Ericaceae	*Enkianthus*	Lour.	*Enkianthus*	T23
Myrtales	Onagraceae	*Epilobium*	L.	*Epilobium*	T23 & this study
Equisetales	Equisetaceae	*Equisetum*	L.	*Equisetum*	T23
Ericales	Ericaceae	*Erica*	L.	*Erica*	T23
Asterales	Asteraceae	*Erigeron*	L.	*Erigeron*	T23 & this study
Ranunculales	Ranunculaceae	*Eriocapitella*	Nakai	*Eriocapitella*	T23
Geraniales	Geraniaceae	*Erodium*	L'Hér.	*Erodium*	T23 & this study
Brassicales	Brassicaceae	*Eruca*	Mill.	*Eruca*	T23
Apiales	Apiaceae	*Eryngium*	L.	*Eryngium*	T23 & this study
Brassicales	Brassicaceae	*Erysimum*	Tourn. ex L.	*Erysimum*	T23
Lamiales	Phrymaceae	*Erythranthe*	Spach	*Erythranthe*	T23
Escalloniales	Escalloniaceae	*Escallonia*	Mutis ex L.f.	*Escallonia*	T23
Ranunculales	Papaveraceae	*Eschscholzia*	Cham.	*Eschscholzia*	T23
Myrtales	Myrtaceae	*Eucalyptus*	L'Hér.	*Eucalyptus*	T23
Asparagales	Asparagaceae	*Eucomis*	L'Hér.	*Eucomis*	T23
Celastrales	Celastraceae	*Euonymus*	L.	*Euonymus*	T23
Asterales	Asteraceae	*Eupatorium*	L.	*Eupatorium*	T23
Malphigiales	Euphorbiaceae	*Euphorbia*	L.	*Euphorbia*	T23 & this study
Lamiales	Orobanchaceae	*Euphrasia*	L.	*Euphrasia*	T23
Asterales	Asteraceae	*Eurybia*	(Cass.) Cass.	*Eurybia*	T23
Asterales	Asteraceae	*Euthamia*	Elliott	*Euthenia*	T23
Asterales	Asteraceae	*Eutrochium*	Raf.	*Eutrochium*	T23
Caryophyllales	Polygonaceae	*Fagopyrum*	Mill.	*Fagopyrum*	T23
Fagales	Fagaceae	*Fagus*	L.	*Fagus*	T23
Apiales	Apiaceae	*Falcaria*	Fabr.	*Falcaria*	T23 & this study
Caryophyllales	Polygonaceae	*Fallopia*	Adans.	*Fallopia*	T23
Poales	Bromeliaceae	*Fascicularia*	Mez	*Fascicularia*	T23
Asterales	Asteraceae	*Felicia*	Cass.	*Felicia*	T23
Poales	Poaceae	*Festuca*	Tourn. ex L.	*Festuca*	T23
Rosales	Moraceae	*Ficus*	L.	*Ficus*	T23
Rosales	Rosaceae	*Filipendula*	Mill.	*Filipendula*	T23 & this study
Apiales	Apiaceae	*Foeniculum*	Hill	*Foeniculum*	T23 & this study
Lamiales	Oleaceae	*Forsythia*	Vahl	*Forsythia*	T23
Rosales	Rosaceae	*Fragaria*	L.	*Fragaria*	T23
Lamiales	Oleaceae	*Fraxinus*	Tourn. ex L.	*Fraxinus*	T23
Myrtales	Onagraceae	*Fuchsia*	L.	*Fuchsia*	T23
Ranunculales	Papaveraceae	*Fumaria*	L.	*Fumaria*	T23
Asterales	Asteraceae	*Galactites*	Moench	*Galactites*	T23 & this study
Fabales	Fabaceae	*Galega*	Tourn. ex L.	*Galega*	T23
Lamiales	Lamiaceae	*Galeopsis*	L.	*Galeopsis*	T23
Asterales	Asteraceae	*Galinsoga*	Ruiz & Pav.	*Galinsoga*	T23
Gentianales	Rubiaceae	*Galium*	L.	*Galium*	T23 & this study
Asterales	Asteraceae	*Gamochaeta*	Wedd.	*Gamochaeta*	T23
Asterales	Asteraceae	*Gazania*	Gaertn.	*Gazania*	T23
Fabales	Fabaceae	*Genista*	L.	*Genista*	T23
Gentianales	Gentianaceae	*Gentiana*	Tourn. ex L.	*Gentiana*	T23
Geraniales	Geraniaceae	*Geranium*	Tourn. ex L.	*Geranium*	T23 & this study
Rosales	Rosaceae	*Geum*	L.	*Geum*	T23
Asparagales	Iridaceae	*Gladiolus*	Tourn. ex L.	*Gladiolus*	T23
Boraginales	Boraginaceae	*Glandora*	D.C.Thomas, Weigend & Hilger	*Glandora*	T23
Asterales	Asteraceae	*Glebionis*	Cass.	*Chrysanthemum*	T23
Asterales	Asteraceae	*Glebionis*	Cass.	*Glebionis*	T23
Lamiales	Lamiaceae	*Glechoma*	L.	*Glechoma*	T23 & this study

Order	Family	Genus	Author	Name in Thompson et al. (2023)	Source
Fabales	Fabaceae	*Glycine*	Willd.	*Glycine*	T23
Asterales	Asteraceae	*Gnaphalium*	L.	*Gnaphalium*	T23
Malvales	Malvaceae	*Gossypium*	L.	*Gossypium*	T23
Proteales	Proteaceae	*Grevillea*	R.Br.	*Grevillea*	T23
Ranunculales	Ranunculaceae	*Halerpestes*	Greene	*Ranunculus*	T23
Apiales	Araliaceae	*Hedera*	L.	*Hedera*	T23
Zingiberales	Zingiberaceae	*Hedychium*	J.Koenig	*Hedychium*	T23
Asterales	Asteraceae	*Hedypnois*	Hill	*Hedypnois*	T23
Malvales	Cistaceae	*Helianthemum*	Mill.	*Helianthemum*	T23
Asterales	Asteraceae	*Helianthus*	L.	*Helianthus*	T23
Asterales	Asteraceae	*Helianthus*	L.	*Helicanthus*	T23
Asterales	Asteraceae	*Helichrysum*	Mill.	*Helichrysum*	T23
Poales	Poaceae	*Helictotrichon*	Besser	*Helictotrichon*	T23
Asterales	Asteraceae	*Heliopsis*	Pers.	*Heliopsis*	T23
Boraginales	Heliotropiaceae	*Heliotropium*	L.	*Heliotropium*	T23
Asterales	Asteraceae	*Helminthotheca*	Zinn	*Helminthoteca*	T23 & this study
Apiales	Apiaceae	*Helosciadium*	W.D.J.Koch	*Apium*	T23
Asparagales	Asphodelaceae	*Hemerocallis*	L.	*Hemerocallis*	T23
Asterales	Asteraceae	*Hemizonia*	DC.	*Hemizonia*	T23
Apiales	Apiaceae	*Heracleum*	L.	*Heracleum*	T23
Asparagales	Iridaceae	*Hesperantha*	Ker Gawl.	*Hesperantha*	T23
Brassicales	Brassicaceae	*Hesperis*	L.	*Hesperis*	T23
Saxifragales	Saxifragaceae	*Heuchera*	L.	*Heuchera*	T23
Malvales	Malvaceae	*Hibiscus*	L.	*Hibiscus*	T23
Asterales	Asteraceae	*Hieracium*	L.	*Hieracium*	T23
Fabales	Fabaceae	*Hippocrepis*	L.	*Hippocrepis*	T23
Rosales	Elaeagnaceae	*Hippophae*	L.	*Hippophae*	T23
Lamiales	Plantaginaceae	*Hippuris*	L.	*Hippuris*	T23
Poales	Poaceae	*Holcus*	L.	*Holcus*	T23 & this study
Poales	Poaceae	*Hordeum*	L.	*Hordeum*	T23
Piperales	Saururaceae	*Houttuynia*	Thunb.	*Houttuynia*	T23
Rosales	Cannabaceae	*Humulus*	L.	*Humulus*	T23
Asparagales	Asparagaceae	*Hyacinthoides*	Heist. ex Fabr.	*Hyacinthoides*	T23
Cornales	Hydrangeaceae	*Hydrangea*	Gronov. ex L.	*Hydrangea*	T23
Saxifragales	Crassulaceae	*Hylotelephium*	H.Ohba	*Hylotelephium*	T23
Asterales	Asteraceae	*Hyoseris*	L.	*Hyoseris*	T23
Malpighiales	Hypericaceae	*Hypericum*	L.	*Hypericum*	T23 & this study
Asterales	Asteraceae	*Hypochaeris*	L.	*Hypochaeris*	T23 & this study
Lamiales	Lamiaceae	*Hyssopus*	L.	*Hyssopus*	T23
Brassicales	Brassicaceae	*Iberis*	Dill. ex L.	*Iberis*	T23
Aquifoliales	Aquifoliaceae	*Ilex*	L.L.	*Ilex*	T23
Ericales	Balsaminaceae	*Impatiens*	L.	*Impatiens*	T23
Asterales	Asteraceae	*Inula*	L.	*Inula*	T23
Solanales	Convolvulaceae	*Ipomoea*	L.	*Ipomea*	T23
Solanales	Convolvulaceae	*Ipomoea*	L.	*Ipomoea*	T23
Asparagales	Iridaceae	*Iris*	Tourn. ex L.	*Iris*	T23
Asterales	Asteraceae	*Jacobaea*	Burm.	*Jacobaea*	T23 & this study
Asterales	Campanulaceae	*Jasione*	L.	*Jasione*	T23
Lamiales	Oleaceae	*Jasminum*	L.	*Jasminum*	T23
Fagales	Juglandaceae	*Juglans*	L.	*Juglans*	T23
Poales	Juncaceae	*Juncus*	L.	*Juncus*	T23
Pinales	Cupressaceae	*Juniperus*	L.	*Juniperus*	T23
Rosales	Rosaceae	*Kerria*	DC.	*Kerria*	T23
Dipsacales	Caprifoliaceae	*Knautia*	L.	*Knautia*	T23
Asparagales	Asphodelaceae	*Kniphofia*	Moench	*Kniphofia*	T23
Malvales	Malvaceae	*Kokia*	Lewton	*Kokia*	T23
Dipsacales	Caprifoliaceae	*Kolkwitzia*	Graebn.	*Linnaea*	T23
Asterales	Asteraceae	*Lactuca*	L.	*Lactuca*	T23
Poales	Poaceae	*Lagurus*	L.	*Lagurus*	T23
Proteales	Proteaceae	*Lambertia*	Sm.	*Lambertia*	T23
Lamiales	Lamiaceae	*Lamium*	L.	*Lamium*	T23
Asterales	Asteraceae	*Lapsana*	L.	*Lapsana*	T23
Apiales	Apiaceae	*Laserpitium*	L.	*Laserpitium*	T23
Fabales	Fabaceae	*Lathyrus*	L.	*Lathyrus*	T23 & this study
Fabales	Fabaceae	*Lathyrus*	L.	*Pisum*	T23 & this study

Order	Family	Genus	Author	Name in Thompson et al. (2023)	Source
Laurales	Lauraceae	*Laurus*	L.	*Laurus*	T23
Lamiales	Lamiaceae	*Lavandula*	L.	*Lavandula*	T23
Asterales	Campanulaceae	*Legousia*	Durande	*Legousia*	T23
Asterales	Asteraceae	*Leontodon*	L.	*Leontodon*	T23
Brassicales	Brassicaceae	*Lepidium*	L.	*Lepidium*	T23
Ericales	Ericaceae	*Leptecophylla*	C.M.Weiller	*Leptecophylla*	T23
Myrtales	Myrtaceae	*Leptospermum*	J.R.Forst. & G.Forst.	*Leptospermum*	T23
Asterales	Asteraceae	*Leucanthemum*	Hill	*Leucanthemum*	T23 & this study
Asterales	Asteraceae	*Leuzea*	DC.	*Rhaponticum*	T23
Apiales	Apiaceae	*Levisticum*	Hill	*Levisticum*	T23
Poales	Poaceae	*Leymus*	Hochst.	*Leymus*	T23
Asparagales	Iridaceae	*Libertia*	Spreng.	*Libertia*	T23
Apiales	Apiaceae	*Ligusticum*	L.	*Ligusticum*	T23
Lamiales	Oleaceae	*Ligustrum*	L.	*Ligustrum*	T23
Liliales	Liliaceae	*Lilium*	Tourn. ex L.	*Lilium*	T23
Lamiales	Plantaginaceae	*Linaria*	Mill.	*Linaria*	T23
Malpighiales	Linaceae	*Linum*	L.	*Linum*	T23
Saxifragales	Altingiaceae	*Liquidambar*	L.	*Liquidambar*	T23
Boraginales	Boraginaceae	*Lithospermum*	L.	*Lithospermum*	T23
Asterales	Campanulaceae	*Lobelia*	Plum. ex L.	*Lobelia*	T23
Asterales	Asteraceae	*Logfia*	Cass.	*Logfia*	T23
Poales	Poaceae	*Lolium*	L.	*Festuca*	T23 & this study
Poales	Poaceae	*Lolium*	L.	*Lolium*	T23 & this study
Dipsacales	Caprifoliaceae	*Lomelosia*	Raf.	*Lomelosia*	T23
Dipsacales	Caprifoliaceae	*Lonicera*	L.	*Lonicera*	T23
Asterales	Asteraceae	*Lophiolepis*	Cass.	*Cirsium*	T23
Fabales	Fabaceae	*Lotus*	L.	*Lotus*	T23 & this study
Brassicales	Brassicaceae	*Lunaria*	Tourn. ex L.	*Lunaria*	T23
Fabales	Fabaceae	*Lupinus*	L.	*Lupinus*	T23
Lamiales	Lamiaceae	*Lycopus*	L.	*Lycopus*	T23
Ericales	Primulaceae	*Lysimachia*	Tourn. ex L.	*Anagallis*	T23
Ericales	Primulaceae	*Lysimachia*	Tourn. ex L.	*Glaux*	T23
Ericales	Primulaceae	*Lysimachia*	Tourn. ex L.	*Lysimachia*	T23
Myrtales	Lythraceae	*Lythrum*	L.	*Lythrum*	T23
Asterales	Asteraceae	*Madia*	Molina	*Madia*	T23
Magnoliales	Magnoliaceae	*Magnolia*	Plum. ex L.	*Magnolia*	T23
Ranunculales	Berberidaceae	*Mahonia*	Nutt.	*Mahonia*	T23
Asparagales	Asparagaceae	*Maianthemum*	F.H.Wigg.	*Maianthemum*	T23
Rosales	Rosaceae	*Malus*	Mill.	*Malus*	T23
Malvales	Malvaceae	*Malva*	L.	*Malva*	T23 & this study
Cucurbitales	Cucurbitaceae	*Marah*	Kellogg	*Echinocystis*	T23
Rosales	Rosaceae	*Margyricarpus*	Ruiz & Pav.	*Margyricarpus*	T23
Asterales	Asteraceae	*Matricaria*	L.	*Matricaria*	T23
Brassicales	Brassicaceae	*Matthiola*	W.T.Aiton	*Matthiola*	T23
Fabales	Fabaceae	*Medicago*	L.	*Medicago*	T23 & this study
Myrtales	Myrtaceae	*Melaleuca*	L.	*Calothamnus*	T23
Lamiales	Orobanchaceae	*Melampyrum*	L.	*Melampyrum*	T23
Poales	Poaceae	*Melica*	L.	*Melica*	T23
Fabales	Fabaceae	*Melilotus*	Mill.	*Melilotus*	T23
Lamiales	Lamiaceae	*Melissa*	L.	*Melissa*	T23
Lamiales	Lamiaceae	*Mentha*	L.	*Mentha*	T23 & this study
Asterales	Menyanthaceae	*Menyanthes*	L.	*Menyanthes*	T23
Malpighiales	Euphorbiaceae	*Mercurialis*	L.	*Mercurialis*	T23
Myrtales	Myrtaceae	*Metrosideros*	Banks ex Gaertn.	*Metrosideros*	T23
Poales	Poaceae	*Milium*	L.	*Milium*	T23
Malvales	Malvaceae	*Modiola*	Moench	*Modiola*	T23
Caryophyllales	Caryophyllaceae	*Moehringia*	L.	*Moehringia*	T23
Poales	Poaceae	*Molinia*	Schrank	*Molinia*	T23
Lamiales	Lamiaceae	*Monarda*	L.	*Monarda*	T23
Fagales	Myricaceae	*Morella*	Lour.	*Myrica*	T23
Rosales	Moraceae	*Morus*	L.	*Morus*	T23
Poales	Poaceae	*Muhlenbergia*	Schreb.	*Muhlenbergia*	T23
Zingiberales	Musaceae	*Musa*	L.	*Musa*	T23
Asparagales	Asparagaceae	*Muscari*	Mill.	*Muscari*	T23
Brassicales	Brassicaceae	*Myagrum*	L.	*Myagrum*	T23
Asterales	Asteraceae	*Mycelis*	Cass.	*Lactuca*	T23

Order	Family	Genus	Author	Name in Thompson et al. (2023)	Source
Boraginales	Boraginaceae	*Myosotis*	L.	*Myosotis*	T23
Fagales	Myricaceae	*Myrica*	L.	*Myrica*	T23
Apiales	Apiaceae	*Myrrhis*	Mill.	*Myrrhis*	T23
Myrtales	Myrtaceae	*Myrtus*	L.	*Myrtus*	T23
Ranunculales	Berberidaceae	*Nandina*	Thunb.	*Nandina*	T23
Asparagales	Amaryllidaceae	*Narcissus*	L.	*Narcissus*	T23
Dioscoreales	Nartheciaceae	*Narthecium*	Huds.	*Narthecium*	T23
Poales	Poaceae	*Nassella*	(Trin.) É.Desv.	*Nassella*	T23
Brassicales	Brassicaceae	*Nasturtium*	W.T.Aiton	*Nasturtium*	T23
Asparagales	Orchidaceae	*Neottia*	Guett.	*Neottia*	T23
Lamiales	Lamiaceae	*Nepeta*	L.	*Nepeta*	T23
Solanales	Solanaceae	*Nicotiana*	L.	*Nicotiana*	T23
Ranunculales	Ranunculaceae	*Nigella*	L.	*Nigella*	T23
Nymphaeales	Nymphaeaceae	*Nymphaea*	L.	*Nymphaea*	T23
Lamiales	Lamiaceae	*Ocimum*	L.	*Ocimum*	T23
Asterales	Asteraceae	*Oclemena*	Greene	*Oclemena*	T23
Lamiales	Orobanchaceae	*Odontites*	Ludw.	*Odontites*	T23
Myrtales	Onagraceae	*Oenothera*	L.	*Oenothera*	T23
Lamiales	Oleaceae	*Olea*	L.	*Olea*	T23
Fabales	Fabaceae	*Onobrychis*	Mill.	*Onobrychis*	T23
Fabales	Fabaceae	*Ononis*	L.	*Ononis*	T23 & this study
Asterales	Asteraceae	*Onopordum*	L.	*Onopordum*	T23
Lamiales	Lamiaceae	*Origanum*	L.	*Origanum*	T23
Asparagales	Asparagaceae	*Ornithogalum*	L.	*Ornithogalum*	T23
Fabales	Fabaceae	*Ornithopus*	L.	*Ornithopus*	T23
Lamiales	Oleaceae	*Osmanthus*	Lour.	*Osmanthus*	T23
Osmundales	Osmundaceae	*Osmunda*	L.	*Osmunda*	T23
Fagales	Betulaceae	*Ostrya*	Scop.	*Ostrya*	T23
Oxalidales	Oxalidaceae	*Oxalis*	L.	*Oxalis*	T23
Ericales	Ericaceae	*Oxydendrum*	DC.	*Oxydendrum*	T23
Asterales	Asteraceae	*Ozothamnus*	R.Br.	*Ozothamnus*	T23
Gentianales	Rubiaceae	*Palicourea*	Aubl.	*Palicourea*	T23
Rosales	Rhamnaceae	*Paliurus*	Mill.	*Arbusto*	T23
Asterales	Asteraceae	*Pallenis*	(Cass.) Cass.	*Pallenis*	T23
Poales	Poaceae	*Panicum*	L.	*Panicum*	T23
Ranunculales	Papaveraceae	*Papaver*	L.	*Papaver*	T23
Rosales	Urticaceae	*Parietaria*	L.	*Parietaria*	T23
Vitales	Vitaceae	*Parthenocissus*	Planch.	*Parthenocissus*	T23
Apiales	Apiaceae	*Pastinaca*	L.	*Pastinaca*	T23
Lamiales	Orobanchaceae	*Pedicularis*	L.	*Pedicularis*	T23
Geraniales	Geraniaceae	*Pelargonium*	L'Hér.	*Pelargonium*	T23
Boraginales	Boraginaceae	*Pentaglottis*	Tausch	*Pentaglottis*	T23
Caryophyllales	Polygonaceae	*Persicaria*	(L.) Mill.	*Persecaria*	T23
Caryophyllales	Polygonaceae	*Persicaria*	(L.) Mill.	*Persicaria*	T23
Caryophyllales	Polygonaceae	*Persicaria*	(L.) Mill.	*Polygonum*	T23
Asterales	Asteraceae	*Petasites*	L.	*Petasites*	T23
Caryophyllales	Caryophyllaceae	*Petrorhagia*	(Ser. ex DC.) Link	*NA*	This study
Saxifragales	Crassulaceae	*Petrosedum*	Grulich	*Petrosedum*	T23
Apiales	Apiaceae	*Petroselinum*	Hill	*Petroselinum*	T23
Apiales	Apiaceae	*Peucedanum*	L.	*Peucedanum*	T23
Boraginales	Hydrophyllaceae	*Phacelia*	Juss.	*Phacelia*	T23
Poales	Poaceae	*Phalaris*	L.	*Phalaris*	T23
Fabales	Fabaceae	*Phaseolus*	L.	*Phaseolus*	T23
Saxifragales	Crassulaceae	*Phedimus*	Raf.	*Phedimus*	T23
Sapindales	Rutaceae	*Phellodendron*	Rupr.	*Phellodendron*	T23
Cornales	Hydrangeaceae	*Philadelphus*	L.	*Philadelphus*	T23
Poales	Poaceae	*Phleum*	L.	*Phleum*	T23
Ericales	Polemoniaceae	*Phlox*	L.	*Phlox*	T23
Rosales	Rosaceae	*Photinia*	Lindl.	*Photinia*	T23
Poales	Poaceae	*Phragmites*	Adans.	*Phragmites*	T23
Lamiales	Scrophulariaceae	*Phygelius*	E.Mey. ex Benth.	*Phygelius*	T23
Malpighiales	Phyllanthaceae	*Phyllanthus*	L.	*Phyllanthus*	T23
Solanales	Solanaceae	*Physalis*	L.	*Physalis*	T23
Rosales	Rosaceae	*Physocarpus*	(Cambess.) Maxim.	*Physocarpus*	T23
Lamiales	Lamiaceae	*Physostegia*	Benth.	*Physostegia*	T23
Asterales	Campanulaceae	*Phyteuma*	L.	*Phyteuma*	T23
Pinales	Pinaceae	*Picea*	A.Dietr.	*Picea*	T23

Order	Family	Genus	Author	Name in Thompson et al. (2023)	Source
Asterales	Asteraceae	*Picris*	L.	*Picris*	T23 & this study
Asterales	Asteraceae	*Pilosella*	Hill	*Pilosella*	T23
Apiales	Apiaceae	*Pimpinella*	L.	*Pimpinella*	T23
Pinales	Pinaceae	*Pinus*	L.	*Pinus*	T23
Rosales	Urticaceae	*Pipturus*	Wedd.	*Pipturus*	T23
Sapindales	Anacardiaceae	*Pistacia*	L.	*Pistacia*	T23
Apiales	Pittosporaceae	*Pittosporum*	Gaertn.	*Pittosporum*	T23
Lamiales	Plantaginaceae	*Plantago*	L.	*Plantago*	T23 & this study
Proteales	Platanaceae	*Platanus*	L.	*Platanus*	T23
Myrtales	Melastomataceae	*Pleroma*	D.Don	*Tibouchina*	T23
Asterales	Asteraceae	*Pluchea*	Cass.	*Pluchea*	T23
Poales	Poaceae	*Poa*	L.	*Poa*	T23 & this study
Ranunculales	Berberidaceae	*Podophyllum*	L.	*Podophyllum*	T23
Ericales	Polemoniaceae	*Polemonium*	L.	*Polemonium*	T23
Caryophyllales	Polygonaceae	*Polygonum*	L.	*Polygonum*	T23
Malpighiales	Salicaceae	*Populus*	L.	*Populus*	T23
Rosales	Rosaceae	*Potentilla*	L.	*Potentilla*	T23 & this study
Rosales	Rosaceae	*Poterium*	L.	*Sanguisorba*	T23 & this study
Ericales	Primulaceae	*Primula*	L.	*Primula*	T23
Lamiales	Lamiaceae	*Prunella*	L.	*Prunella*	T23
Rosales	Rosaceae	*Prunus*	L.	*Prunus*	T23 & this study
Asterales	Asteraceae	*Psephellus*	Cass.	*Psephellus*	T23
Polypodiales	Dennstaedtiaceae	*Pteridium*	Gled. ex Scop.	*Pteridium*	T23
Asterales	Asteraceae	*Pulicaria*	Gaertn.	*Pulicaria*	T23
Rosales	Rosaceae	*Pyracantha*	Roem.	*Pyracantha*	T23
Ericales	Ericaceae	*Pyrola*	L.	*Pyrola*	T23
Rosales	Rosaceae	*Pyrus*	L.	*Pyrus*	T23
Fagales	Fagaceae	*Quercus*	L.	*Quercus*	T23 & this study
Caryophyllales	Caryophyllaceae	*Rabelera*	M.T.Sharples & E.A.Tripp	*Rabelera*	T23
Ranunculales	Ranunculaceae	*Ranunculus*	L.	*Ranunculus*	T23 & this study
Brassicales	Brassicaceae	*Raphanus*	L.	*Raphanus*	T23 & this study
Brassicales	Brassicaceae	*Rapistrum*	Crantz	*Rapistrum*	T23 & this study
Asterales	Asteraceae	*Reichardia*	Roth	*Reichardia*	T23
Brassicales	Resedaceae	*Reseda*	L.	*Reseda*	T23
Caryophyllales	Polygonaceae	*Reynoutria*	Houtt.	*Reynoutria*	T23
Rosales	Rhamnaceae	*Rhamnus*	L.	*Rhamnus*	T23
Asterales	Asteraceae	*Rhaponticoides*	Vaill.	*Centaurea*	T23
Caryophyllales	Polygonaceae	*Rheum*	L.	*Rheum*	T23
Lamiales	Orobanchaceae	*Rhinanthus*	L.	*Rhinanthus*	T23
Sapindales	Anacardiaceae	*Rhus*	L.	*Rhus*	T23
Saxifragales	Grossulariaceae	*Ribes*	L.	*Ribes*	T23
Malpighiales	Euphorbiaceae	*Ricinus*	L.	*Ricinus*	T23
Fabales	Fabaceae	*Robinia*	L.	*Robinia*	T23
Ranunculales	Papaveraceae	*Roemeria*	Medik.	*Roemeria*	T23
Brassicales	Brassicaceae	*Rorippa*	Scop.	*Rorippa*	T23
Rosales	Rosaceae	*Rosa*	L.	*Rosa*	T23
Rosales	Rosaceae	*Rosa*	L.	*Rosa*	T23
Poales	Poaceae	*Rostraria*	Trin.	*Rostraria*	T23
Gentianales	Rubiaceae	*Rubia*	L.	*Rubia*	T23 & this study
Rosales	Rosaceae	*Rubus*	L.	*Rubus*	T23 & this study
Asterales	Asteraceae	*Rudbeckia*	L.	*Rudbeckia*	T23
Caryophyllales	Polygonaceae	*Rumex*	L.	*Rumex*	T23 & this study
Asparagales	Asparagaceae	*Ruscus*	L.	*Ruscus*	T23
Sapindales	Rutaceae	*Ruta*	L.	*Ruta*	T23
Poales	Poaceae	*Sacciolepis*	Nash	*Sacciolepis*	T23

Order	Family	Genus	Author	Name in Thompson et al. (2023)	Source
Caryophyllales	Caryophyllaceae	*Sagina*	L.	*Sagina*	T23
Malpighiales	Salicaceae	*Salix*	L.	*Salix*	T23 & this study
Lamiales	Lamiaceae	*Salvia*	L.	*Salvia*	T23 & this study
Dipsacales	Viburnaceae	*Sambucus*	L.	*Sambucus*	T23
Rosales	Rosaceae	*Sanguisorba*	L.	*Sanguisorba*	T23
Apiales	Apiaceae	*Sanicula*	L.	*Sanicula*	T23
Asterales	Asteraceae	*Santolina*	L.	*Santolina*	T23
Caryophyllales	Caryophyllaceae	*Saponaria*	L.	*Saponaria*	T23
Buxales	Buxaceae	*Sarcococca*	Lindl.	*Sarcococca*	T23
Lamiales	Lamiaceae	*Satureja*	L.	*Satureja*	T23
Dipsacales	Caprifoliaceae	*Scabiosa*	L.	*Scabiosa*	T23
Apiales	Apiaceae	*Scandix*	L.	*Scandix*	T23
Poales	Cyperaceae	*Schoenoplectus*	(Rchb.) Palla	*Schoenoplectus*	T23
Poales	Cyperaceae	*Scirpus*	Tourn. ex L.	*Scirpus*	T23
Caryophyllales	Caryophyllaceae	*Scleranthus*	L.	*Scleranthus*	T23
Asterales	Asteraceae	*Scolymus*	L.	*Scolymus*	T23
Fabales	Fabaceae	*Scorpiurus*	L.	*Scorpiurus*	T23
Asterales	Asteraceae	*Scorzonera*	L.	*Scorzonera*	T23 & this study
Asterales	Asteraceae	*Scorzoneroides*	Moench	*Scorzoneroides*	T23
Lamiales	Scrophulariaceae	*Scrophularia*	L.	*Scrophularia*	T23
Lamiales	Lamiaceae	*Scutellaria*	L.	*Scutellaria*	T23
Poales	Poaceae	*Secale*	L.	*Secale*	T23
Saxifragales	Crassulaceae	*Sedum*	L.	*Sedum*	T23
Asterales	Asteraceae	*Senecio*	L.	*Senecio*	T23
Fabales	Fabaceae	*Senna*	Mill.	*Senna*	T23
Apiales	Apiaceae	*Seseli*	L.	*Seseli*	T23
Caryophyllales	Aizoaceae	*Sesuvium*	L.	*Sesuvium*	T23
Gentianales	Rubiaceae	*Sherardia*	L.	*Sherardia*	T23 & this study
Ericales	Sapotaceae	*Sideroxylon*	L.	*Sideroxylon*	T23
Caryophyllales	Caryophyllaceae	*Silene*	L.	*Silene*	T23 & this study
Caryophyllales	Caryophyllaceae	*Silene*	L.	*Lychnis*	T23 & this study
Asterales	Asteraceae	*Silybum*	Vaill. ex Adans.	*Silybum*	T23
Asparagales	Asphodelaceae	*Simethis*	Kunth	*Simethis*	T23
Brassicales	Brassicaceae	*Sinapis*	L.	*Sinapis*	T23 & this study
Brassicales	Brassicaceae	*Sisymbrium*	L.	*Sisymbrium*	T23
Dipsacales	Caprifoliaceae	*Sixalix*	Raf.	*Scabiosa*	T23 & this study
Apiales	Apiaceae	*Smyrnium*	L.	*Smyrnium*	T23
Solanales	Solanaceae	*Solanum*	L.	*Lycoperscion*	T23
Solanales	Solanaceae	*Solanum*	L.	*Solanum*	T23
Asterales	Asteraceae	*Solidago*	L.	*Solidago*	T23
Asterales	Asteraceae	*Sonchus*	L.	*Sonchus*	T23 & this study
Rosales	Rosaceae	*Sorbaria*	(Ser. ex DC.) A.Br.	*Sorbaria*	T23
Rosales	Rosaceae	*Sorbus*	L.	*Sorbus*	T23
Poales	Poaceae	*Sorghum*	Moench	*Sorghum*	T23
Caryophyllales	Amaranthaceae	*Spinacia*	L.	*Spinacia*	T23
Rosales	Rosaceae	*Spiraea*	L.	*Spiraea*	T23
Lamiales	Lamiaceae	*Stachys*	L.	*Stachys*	T23
Lamiales	Verbenaceae	*Stachytarpheta*	Vahl	*Stachytarpheta*	T23
Crossosomatales	Staphyleaceae	*Staphylea*	L.	*Staphylea*	T23
Caryophyllales	Caryophyllaceae	*Stellaria*	L.	*Stellaria*	T23
Asterales	Asteraceae	*Stokesia*	L'Hér.	*Stokesia*	T23
Ranunculales	Papaveraceae	*Stylophorum*	Nutt.	*Stylophorum*	T23
Dipsacales	Caprifoliaceae	*Succisa*	Haller	*Succisa*	T23 & this study
Dipsacales	Caprifoliaceae	*Succisella*	Beck	*Succisella*	T23
Dipsacales	Caprifoliaceae	*Symphoricarpos*	Dill. ex Juss.	*Symphoricarpos*	T23
Asterales	Asteraceae	*Symphyotrichu*	Nees	*Symphyotrichum*	T23

Order	Family	Genus	Author	Name in Thompson et al. (2023)	Source
		m			
Boraginales	Boraginaceae	*Symphytum*	L.	*Symphytum*	T23
Lamiales	Oleaceae	*Syringa*	L.	*Syringa*	T23
Asterales	Asteraceae	*Tagetes*	L.	*Tagetes*	T23
Malvales	Malvaceae	*Talipariti*	Fryxell	*Hibiscus*	T23
Caryophyllales	Tamaricaceae	*Tamarix*	L.	*Tamarix*	T23
Asterales	Asteraceae	*Tanacetum*	L.	*Chrysanthemum*	T23
Asterales	Asteraceae	*Tanacetum*	L.	*Tanacetum*	T23
Asterales	Asteraceae	*Taraxacum*	Weber	*Taraxacum*	T23 & this study
Pinales	Taxaceae	*Taxus*	L.	*Taxus*	T23
Saxifragales	Saxifragaceae	*Tellima*	R.Br.	*Tellima*	T23
Lamiales	Lamiaceae	*Teucrium*	L.	*Teucrium*	T23
Ranunculales	Ranunculaceae	*Thalictrum*	L.	*Thalictrum*	T23
Gentianales	Rubiaceae	*Theligonum*	L.	*Theligonum*	T23
Polypodiales	Thelypteridaceae	*Thelypteris*	Schmidel	*Thelypteris*	T23
Santalales	Thesiaceae	*Thesium*	L.	*Thesium*	T23
Brassicales	Brassicaceae	*Thlaspi*	Thlaspi	*Thlaspi*	T23
Asterales	Asteraceae	*Thrincia*	Roth	*NA*	This study
Lamiales	Lamiaceae	*Thymus*	L.	*Thymus*	T23
Apiales	Apiaceae	*Thysselinum*	Adans.	*Peucedanum*	T23
Malvales	Malvaceae	*Tilia*	L.	*Tilia*	T23
Asterales	Asteraceae	*Tolpis*	Adans.	*Tolpis*	T23
Apiales	Apiaceae	*Tordylium*	L.	*Tordylium*	T23
Apiales	Apiaceae	*Torilis*	Adans.	*Torilis*	T23 & this study
Sapindales	Anacardiaceae	*Toxicodendron*	Mill.	*Toxicodendron*	T23
Gentianales	Apocynaceae	*Trachelospermum*	Lem.	*Trachelospermum*	T23
Commelinales	Commelinaceae	*Tradescantia*	Ruppius ex L.	*Tradescantia*	T23
Asterales	Asteraceae	*Tragopogon*	L.	*Tragopogon*	T23 & this study
Fabales	Fabaceae	*Trifolium*	Tourn. ex L.	*Trifolium*	T23 & this study
Alismatales	Juncaginaceae	*Triglochin*	Riv. ex L.	*Triglochin*	T23
Asterales	Asteraceae	*Tripleurospermum*	Sch.Bip.	*Tripleurospermum*	T23
Asterales	Asteraceae	*Tripolium*	Nees	*Tripolium*	T23
Poales	Poaceae	*Triticosecale*	×Wittm. ex A.Camus	*xTriticosecale*	T23
Poales	Poaceae	*Triticum*	L.	*Triticum*	T23
Ranunculales	Ranunculaceae	*Trollius*	L.	*Trollius*	T23
Asterales	Asteraceae	*Trommsdorffia*	Bernh.	*Hypochoeris*	T23
Brassicales	Tropaeolaceae	*Tropaeolum*	L.	*Tropaeolum*	T23
Liliales	Liliaceae	*Tulipa*	L.	*Tulipa*	T23
Asterales	Asteraceae	*Tussilago*	L.	*Tussilago*	T23
Asterales	Asteraceae	*Tyrimnus*	(Cass.) Bosc	*NA*	This study
Fabales	Fabaceae	*Ulex*	L.	*Ulex*	T23
Rosales	Ulmaceae	*Ulmus*	L.	*Ulmus*	T23
Saxifragales	Crassulaceae	*Umbilicus*	DC.	*Umbilicus*	T23
Poales	Poaceae	*Urochloa*	P.Beauv.	*Brachiaria*	T23
Asterales	Asteraceae	*Urospermum*	Scop.	*Urospermum*	T23
Rosales	Urticaceae	*Urtica*	L.	*Urtica*	T23
Ericales	Ericaceae	*Vaccinium*	L.	*Vaccinium*	T23
Dipsacales	Caprifoliaceae	*Valeriana*	L.	*Valeriana*	T23
Dipsacales	Caprifoliaceae	*Valerianella*	Mill.	*Valerianella*	T23 & this study
Lamiales	Scrophulariaceae	*Verbascum*	L.	*Verbascum*	T23
Lamiales	Verbenaceae	*Verbena*	L.	*Verbena*	T23
Lamiales	Plantaginaceae	*Veronica*	L.	*Hebe*	T23
Lamiales	Plantaginaceae	*Veronica*	L.	*Veronica*	T23
Lamiales	Plantaginaceae	*Veronicastrum*	Heist. ex Fabr.	*Veronicastrum*	T23
Dipsacales	Viburnaceae	*Viburnum*	L.	*Viburnum*	T23
Fabales	Fabaceae	*Vicia*	L.	*Vicia*	T23 & this study
Gentianales	Apocynaceae	*Vinca*	L.	*Vinca*	T23
Malpighiales	Violaceae	*Viola*	L.	*Viola*	T23
Apiales	Apiaceae	*Visnaga*	Gaertn.	*Ammi*	T23
Vitales	Vitaceae	*Vitis*	L.	*Vitis*	T23
Dipsacales	Caprifoliaceae	*Weigela*	Thunb.	*Weigela*	T23
Malvales	Thymelaeaceae	*Wikstroemia*	Endl.	*Wikstroemia*	T23

Order	Family	Genus	Author	Name in Thompson et al. (2023)	Source
Asterales	Asteraceae	*Wyethia*	Nutt.	*Wyethia*	T23
Apiales	Apiaceae	*Xanthoselinum*	Schur	*Peucedanum*	T23
Alismatales	Araceae	*Zantedeschia*	Spreng.	*Zantedeschia*	T23
Poales	Poaceae	*Zea*	L.	*Zea*	T23

Table S11.2. Plant species listed in Thompson et al. [1] and in the present study as hosts of *P. spumarius*. Taxonomy follows the currently accepted names in GBIF [2] after check with 'get_gbif_taxonomy' function in R [3]. "T23" in the "Source" column stands for "Already reported [1]".

Order	Family	Name	Author	Name in Thompson et al. (2023)	Source
Dipsacales	Caprifoliaceae	*Abelia chinensis*	R.Br.	*Linnaea x grandiflora*	T23
Pinales	Pinaceae	*Abies balsamea*	(L.) Mill.	*Abies balsamea*	T23
Fabales	Fabaceae	*Acacia longifolia*	(Andrews) Willd.	*Acacia longifolia*	T23
Asterales	Asteraceae	*Acanthospermum australe*	(Loefl.) Kuntze	*Acanthospermum australe*	T23
Lamiales	Acanthaceae	*Acanthus mollis*	L.	*Acanthus mollis*	T23
Sapindales	Sapindaceae	*Acer campestre*	L.	*Acer campestre*	T23
Sapindales	Sapindaceae	*Acer negundo*	L.	*Acer negundo*	T23
Sapindales	Sapindaceae	*Acer palmatum*	Thunb.	*Acer palmatum*	T23
Sapindales	Sapindaceae	*Acer platanoides*	L.	*Acer platanoides*	T23
Sapindales	Sapindaceae	*Acer pseudoplatanus*	L.	*Acer pseudoplatanus*	T23
Sapindales	Sapindaceae	*Acer pycnanthum*	K.Koch	*Acer pycnanthum*	T23
Sapindales	Sapindaceae	*Acer rubrum*	L.	*Acer rubrum*	T23
Sapindales	Sapindaceae	*Acer spicatum*	Lam.	*Acer spicatum*	T23
Asterales	Asteraceae	*Achillea ageratum*	L.	*Achillea ageratum*	T23
Asterales	Asteraceae	*Achillea filipendulina*	Lam.	*Achillea filipendulina*	T23
Asterales	Asteraceae	*Achillea millefolium*	L.	*Achillea millefolium*	T23 & this study
Asterales	Asteraceae	*Achillea ptarmica*	L.	*Achillea ptarmica*	T23
Acorales	Acoraceae	*Acorus calamus*	L.	*Acorus calamus*	T23
Proteales	Proteaceae	*Adenanthos cuneatus*	Labill.	*Adenanthos cuneatus*	T23
Asterales	Asteraceae	*Adenostyles alpina*	(L.) Bluff & Fingerh.	*Adenostyles alpina*	T23
Apiales	Apiaceae	*Aegopodium podagraria*	L.	*Aegopodium podagraria*	T23
Sapindales	Sapindaceae	*Aesculus hippocastanum*	L.	*Aesculus hippocastanum*	T23
Sapindales	Sapindaceae	*Aesculus parviflora*	Walter	*Aesculus parviflora*	T23
Lamiales	Lamiaceae	*Agastache foeniculum*	(Pursh) Kuntze	*Agastache foeniculum*	T23
Lamiales	Lamiaceae	*Agastache rugosa*	(Fisch. & C.A.Mey.) Kuntze	*Agastache rugosa*	T23
Rosales	Rosaceae	*Agrimonia eupatoria*	L.	*Agrimonia eupatoria*	T23
Caryophyllales	Caryophyllaceae	*Agrostemma githago*	L.	*Agrostemma githago*	T23
Poales	Poaceae	*Agrostis canina*	L.	*Agrostis canina*	T23
Poales	Poaceae	*Agrostis capillaris*	L.	*Agrostis capillaris*	T23
Poales	Poaceae	*Agrostis gigantea*	Roth	*Agrostis gigantea*	T23
Poales	Poaceae	*Agrostis stolonifera*	L.	*Agrostis stolonifera*	T23
Lamiales	Lamiaceae	*Ajuga reptans*	L.	*Ajuga reptans*	T23
Malvales	Malvaceae	*Alcea rosea*	L.	*Alcea rosea*	T23
Rosales	Rosaceae	*Alchemilla glabra*	Neygenf.	*Alchemilla glabra*	T23
Rosales	Rosaceae	*Alchemilla mollis*	(Buser) Rothm.	*Alchemilla mollis*	T23
Rosales	Rosaceae	*Alchemilla vulgaris*	L.	*Alchemilla vulgaris*	T23
Alismatales	Alismataceae	*Alisma triviale*	Pursh	*Alisma triviale*	T23
Solanales	Solanaceae	*Alkekengi officinarum*	Moench	*Physalis alkekengi*	T23
Brassicales	Brassicaceae	*Alliaria petiolata*	(M.Bieb.) Cavara & Grande	*Alliaria petiolata*	T23
Asparagales	Amaryllidaceae	*Allium cepa*	L.	*Allium cepa*	T23
Asparagales	Amaryllidaceae	*Allium proliferum*	Allium ×proliferum (Moench) Schrad. ex Willd.	*Allium × proliferum*	T23
Asparagales	Amaryllidaceae	*Allium sativum*	L.	*Allium sativum*	T23
Asparagales	Amaryllidaceae	*Allium schoenoprasum*	L.	*Allium schoenoprasum*	T23
Asparagales	Amaryllidaceae	*Allium schubertii*	Zucc.	*Allium schubertii*	T23
Asparagales	Amaryllidaceae	*Allium siculum*	Ucria	*Allium siculum*	T23
Asparagales	Amaryllidaceae	*Allium sphaerocephalon*	L.	*Allium sphaerocephalon*	T23
Asparagales	Amaryllidaceae	*Allium triquetrum*	L.	*Allium triquetrum*	T23
Asparagales	Amaryllidaceae	*Allium vineale*	L.	*Allium vineale*	T23
Fagales	Betulaceae	*Alnus glutinosa*	(L.) Gaertn.	*Alnus glutinosa*	T23
Fagales	Betulaceae	*Alnus incana*	(L.) Moench	*Alnus incana*	T23
Fagales	Betulaceae	*Alnus rubra*	Bong.	*Alnus rubra*	T23
Asparagales	Asphodelaceae	*Aloe vera*	(L.) Burm.f.	*Aloe vera*	T23
Poales	Poaceae	*Alopecurus myosuroides*	Huds.	*Alopecurus myosuroides*	T23
Poales	Poaceae	*Alopecurus pratensis*	L.	*Alopercurus pratensis*	T23
Lamiales	Verbenaceae	*Aloysia citrodora*	Paláu	*Aloysia citrodora*	T23
Caryophyllales	Amaranthaceae	*Alternanthera dentata*	(Moench) Stuchl., 1913	*Alternanthera dentata*	T23
Malvales	Malvaceae	*Althaea cannabina*	L.	*Althaea cannabina*	T23 & this study

Order	Family	Name	Author	Name in Thompson et al. (2023)	Source
Malvales	Malvaceae	*Althaea officinalis*	L.	*Althaea officinalis*	T23
Gentianales	Apocynaceae	*Alyxia stellata*	(J.R.Forst. & G.Forst.) Roem. & Schult.	*Alyxia stellata*	T23
Caryophyllales	Amaranthaceae	*Amaranthus retroflexus*	L.	*Amaranthus retroflexus*	T23
Asterales	Asteraceae	*Ambrosia artemisiifolia*	L.	*Ambrosia artemisiifolia*	T23
Asterales	Asteraceae	*Ambrosia trifida*	L.	*Ambrosia trifida*	T23
Rosales	Rosaceae	*Amelanchier humilis*	Wiegand	*Amelanchier spicata*	T23
Apiales	Apiaceae	*Ammi majus*	L.	*Ammi majus*	T23
Boraginales	Boraginaceae	*Amsinckia menziesii*	(Lehm.) A.Nelson & J.F.Macbr.	*Amsinckia menziesii*	T23
Boraginales	Boraginaceae	*Amsinckia spectabilis*	Fisch. & C.A.Mey.	*Amsinckia spectabilis*	T23
Asterales	Asteraceae	*Anacyclus clavatus*	(Desf.) Pers.	*NA*	This study
Asterales	Asteraceae	*Anaphalis margaritacea*	(L.) Benth.	*Anaphalis margaritacea*	T23
Boraginales	Boraginaceae	*Anchusa officinalis*	L.	*Anchusa officinalis*	T23
Ericales	Ericaceae	*Andromeda polifolia*	L.	*Andromeda polifolia*	T23
Asterales	Asteraceae	*Andryala integrifolia*	L.	*Andryala integrifolia*	T23
Poales	Poaceae	*Anemanthele lessoniana*	(Steud.) Veldkamp	*Anemanthele lessoniana*	T23
Ranunculales	Ranunculaceae	*Anemone sylvestris*	L.	*Anemonoides sylvestris*	T23
Apiales	Apiaceae	*Anethum graveolens*	L.	*Anethum graveolens*	T23
Apiales	Apiaceae	*Angelica archangelica*	L.	*Angelica archangelica*	T23
Apiales	Apiaceae	*Angelica sylvestris*	L.	*Angelica sylvestris*	T23
Asterales	Asteraceae	*Antennaria plantaginifolia*	(L.) Hook.	*Antennaria plantaginifolia*	T23
Asterales	Asteraceae	*Anthemis arvensis*	L.	*Anthemis arvensis*	T23
Asterales	Asteraceae	*Anthemis chia*	L.	*Anthemis chia*	T23
Asterales	Asteraceae	*Anthemis cotula*	L.	*Anthemis cotula*	T23
Poales	Poaceae	*Anthoxanthum odoratum*	L.	*Anthoxanthum odoratum*	T23 & this study
Apiales	Apiaceae	*Anthriscus caucalis*	M.Bieb.	*Anthriscus vulgaris*	T23
Apiales	Apiaceae	*Anthriscus cerefolium*	(L.) Hoffm.	*Anthriscus cerefolium*	T23
Apiales	Apiaceae	*Anthriscus sylvestris*	(L.) Hoffm.	*Anthriscus sylvestris*	T23 & this study
Fabales	Fabaceae	*Anthyllis vulneraria*	L.	*Anthyllis vulneraria*	T23
Lamiales	Plantaginaceae	*Antirrhinum majus*	L.	*Antirrhinum majus*	T23
Poales	Poaceae	*Apera spica-venti*	(L.) P.Beauv.	*Apera spica-venti*	T23
Apiales	Apiaceae	*Apium graveolens*	L.	*Apium graveolens*	T23
Ranunculales	Ranunculaceae	*Aquilegia canadensis*	L.	*Aquilegia canadensis*	T23
Ranunculales	Ranunculaceae	*Aquilegia chrysantha*	A.Gray	*Aquilegia chrysantha*	T23
Ranunculales	Ranunculaceae	*Aquilegia vulgaris*	L.	*Aquilegia vulgaris*	T23
Brassicales	Brassicaceae	*Arabis caucasica*	Willd.	*Arabis caucasica*	T23
Fabales	Fabaceae	*Arachis hypogaea*	L.	*Arachis hypogaea*	T23
Ericales	Ericaceae	*Arbutus unedo*	L.	*Arbutus unedo*	T23
Asterales	Asteraceae	*Arctium lappa*	L.	*Arctium lappa*	T23
Asterales	Asteraceae	*Arctium minus*	(Hill) Bernh.	*Arctium minus*	T23
Asterales	Asteraceae	*Arctium tomentosum*	Mill.	*Arctium tormentosum*	T23
Asterales	Asteraceae	*Arctotheca calendula*	(L.) Levyns	*Arctotheca calendula*	T23
Rosales	Rosaceae	*Argentina anserina*	(L.) Rydb.	*Argentina anserina*	T23
Rosales	Rosaceae	*Argentina anserina*	(L.) Rydb.	*Potentilla anserina*	T23
Asterales	Asteraceae	*Argyranthemum frutescens*	(L.) Sch.Bip.	*Argyranthemum frutescens*	T23
Fabales	Fabaceae	*Argyrolobium biebersteinii*	P.W.Ball	*Argyrolobium biebersteinii*	T23
Caryophyllales	Plumbaginaceae	*Armeria maritima*	(Mill.) Willd.	*Armeria maritima*	T23
Brassicales	Brassicaceae	*Armoracia rusticana*	P.Gaertn., B.Mey. & Scherb.	*Armoracia rusticana*	T23
Asterales	Asteraceae	*Arnica montana*	L.	*Arnica montana*	T23
Poales	Poaceae	*Arrhenatherum elatius*	(L.) P.Beauv. ex J.Presl & C.Presl	*Arrhenatherum elatius*	T23 & this study
Asterales	Asteraceae	*Artemisia abrotanum*	L.	*Artemisia abrotanum*	T23
Asterales	Asteraceae	*Artemisia absinthium*	L.	*Artemisia absinthium*	T23
Asterales	Asteraceae	*Artemisia campestris*	L.	*Artemisia campestris*	T23
Asterales	Asteraceae	*Artemisia campestris*	L.	*Artemisia caudata*	T23
Asterales	Asteraceae	*Artemisia dracunculus*	L.	*Artemisia dracunculus*	T23
Asterales	Asteraceae	*Artemisia tridentata*	(Nutt.) W.A.Weber	*Artemisia tridentata*	T23
Asterales	Asteraceae	*Artemisia verlotiorum*	Lamotte	*Artemisia verlotiorum*	T23
Asterales	Asteraceae	*Artemisia vulgaris*	L.	*Artemisia vulgaris*	T23
Alismatales	Araceae	*Arum italicum*	Mill.	*Arum italicum*	T23
Rosales	Rosaceae	*Aruncus sylvester*	Kostel.	*Aruncus sylvester*	T23
Poales	Poaceae	*Arundo donax*	L.	*Arundo donax*	T23

Order	Family	Name	Author	Name in Thompson et al. (2023)	Source
Asparagales	Asparagaceae	*Asparagus officinalis*	L.	*Asparagus officinalis*	T23
Boraginales	Boraginaceae	*Asperugo procumbens*	L.	*Asperugo procumbens*	T23
Asparagales	Asphodelaceae	*Asphodelus ramosus*	L.	*Asphodelus ramosus*	T23
Asterales	Asteraceae	*Aster alpinus*	L.	*Aster alpinus*	T23
Asterales	Asteraceae	*Aster amellus*	L.	*Aster amellus*	T23
Apiales	Apiaceae	*Astrantia major*	L.	*Astrantia major*	T23
Polypodiales	Athyriaceae	*Athyrium filix-femina*	(L.) Roth	*Athyrium filix-femina*	T23
Caryophyllales	Amaranthaceae	*Atriplex prostrata*	DC.	*Atriplex prostrata*	T23
Brassicales	Brassicaceae	*Aubrieta deltoidea*	(L.) DC.	*Aubrieta deltoidea*	T23
Poales	Poaceae	*Avena barbata*	Pott ex Link	*Avena barbata*	T23 & this study
Poales	Poaceae	*Avena fatua*	L.	*Avena fatua*	T23
Poales	Poaceae	*Avena sativa*	L.	*Avena sativa*	T23
Poales	Poaceae	*Avena sterilis*	L.	*Avena sterilis*	T23 & this study
Asterales	Asteraceae	*Baccharis pilularis*	DC.	*Baccharis pilularis*	T23
Asterales	Asteraceae	*Bahia ambrosioides*	Lag.	*Bahia ambrosioides*	T23
Fabales	Fabaceae	*Baptisia australis*	(L.) R.Br.	*Baptisia australis*	T23
Brassicales	Brassicaceae	*Barbarea stricta*	Andrz. ex Besser	*Barbarea stricta*	T23
Brassicales	Brassicaceae	*Barbarea verna*	(Mill.) Asch.	*Barbarea verna*	T23
Brassicales	Brassicaceae	*Barbarea vulgaris*	(L.) W.T.Aiton	*Barbarea vulgaris*	T23
Asterales	Asteraceae	*Bellis perennis*	L.	*Bellis perennis*	T23 & this study
Ranunculales	Berberidaceae	*Berberis darwinii*	Hook.	*Berberis darwinii*	T23
Ranunculales	Berberidaceae	*Berberis julianae*	C.K.Schneid.	*Berberis julianae*	T23
Ranunculales	Berberidaceae	*Berberis thunbergii*	DC.	*Berberis thunbergii*	T23
Saxifragales	Saxifragaceae	*Bergenia crassifolia*	(L.) Fritsch	*Bergenia crassifolia*	T23
Caryophyllales	Amaranthaceae	*Beta vulgaris*	L.	*Beta vulgaris*	T23
Fagales	Betulaceae	*Betula alleghaniensis*	Britton	*Betula alleghaniensis*	T23
Fagales	Betulaceae	*Betula nigra*	L.	*Betula nigra*	T23
Fagales	Betulaceae	*Betula papyrifera*	Marshall	*Betula papyrifera*	T23
Fagales	Betulaceae	*Betula pendula*	Roth	*Betula pendula*	T23
Fagales	Betulaceae	*Betula populifolia*	Marshall	*Betula populifolia*	T23
Fagales	Betulaceae	*Betula pubescens*	Ehrh.	*Betula pubescens*	T23
Asterales	Asteraceae	*Bidens pilosa*	L.	*Bidens pilosa*	T23
Asterales	Asteraceae	*Bidens tripartita*	L.	*Bidens tripartida*	T23
Apiales	Pittosporaceae	*Billardiera heterophylla*	(Lindl.) L.W.Cayzer & Crisp	*Billardiera heterophylla*	T23
Caryophyllales	Polygonaceae	*Bistorta officinalis*	Raf.	*Bistorta officinalis*	T23
Caryophyllales	Polygonaceae	*Bistorta vivipara*	(L.) Delarbre	*Bistorta vivipara*	T23
Fabales	Fabaceae	*Bituminaria bituminosa*	(L.) C.H.Stirt.	*Psorolea bituminosa*	T23
Boraginales	Boraginaceae	*Borago officinalis*	L.	*Borago officinalis*	T23
Asterales	Asteraceae	*Brachyglottis greyi*	(Hook.fil.) B.Nord.	*Brachyglottis grayi*	T23
Poales	Poaceae	*Brachypodium pinnatum*	(L.) P.Beauv.	*Brachypodium pinnatum*	T23
Brassicales	Brassicaceae	*Brassica napus*	L.	*Brassica napus*	T23
Brassicales	Brassicaceae	*Brassica nigra*	(L.) W.D.J.Koch	*Brassica nigra*	T23
Brassicales	Brassicaceae	*Brassica oleracea*	L.	*Brassica oleracea*	T23
Brassicales	Brassicaceae	*Brassica rapa*	L.	*Brassica rapa*	T23
Poales	Poaceae	*Bromus catharticus*	Vahl	*Bromus willenowii*	T23
Poales	Poaceae	*Bromus hordeaceus*	L.	*Bromus hordeaceus*	T23 & this study
Poales	Poaceae	*Bromus secalinus*	L.	*Bromus secalinus*	T23
Poales	Poaceae	*Bromus sterilis*	L.	*Bromus sterilis*	T23
Poales	Poaceae	*Bromus tectorum*	L.	*Bromus tectorum*	T23
Lamiales	Scrophulariaceae	*Buddleja alternifolia*	Maxim.	*Buddleja alternifolia*	T23
Lamiales	Scrophulariaceae	*Buddleja davidii*	Franch.	*Buddleia davidii*	T23
Lamiales	Scrophulariaceae	*Buddleja globosa*	Hope	*Buddleja globosa*	T23
Asterales	Asteraceae	*Buphthalmum salicifolium*	L.	*Buphthalmum salicifolium*	T23
Buxales	Buxaceae	*Buxus sempervirens*	L.	*Buxus sempervirens*	T23
Asterales	Asteraceae	*Calendula arvensis*	L.	*Calendula arvensis*	T23 & this study
Asterales	Asteraceae	*Calendula officinalis*	L.	*Calendula officinalis*	T23
Fabales	Fabaceae	*Calicotome villosa*	(Poir.) Link	*Cytisus laniger*	T23
Ericales	Ericaceae	*Calluna vulgaris*	(L.) Hull	*Calluna vulgaris*	T23
Asterales	Asteraceae	*Calocephalus brownii*	(Cass.) F.Muell.	*Leucophyta brownii*	T23
Ranunculales	Ranunculaceae	*Caltha palustris*	L.	*Caltha palustris*	T23
Solanales	Convolvulaceae	*Calystegia sepium*	(L.) R.Br.	*Calystegia sepium*	T23
Solanales	Convolvulaceae	*Calystegia silvatica*	(Kit.) Griseb.	*Calystegia silvatica*	T23
Asterales	Campanulaceae	*Campanula glomerata*	L.	*Campanula glomerata*	T23
Asterales	Campanulaceae	*Campanula latifolia*	L.	*Campanula latifolia*	T23
Asterales	Campanulaceae	*Campanula medium*	L.	*Campanula medium*	T23

Order	Family	Name	Author	Name in Thompson et al. (2023)	Source
Asterales	Campanulaceae	*Campanula patula*	L.	*Campanula patula*	T23
Asterales	Campanulaceae	*Campanula persicifolia*	L.	*Campanula persicifolia*	T23
Asterales	Campanulaceae	*Campanula portenschlagiana*	Schult.	*Campanula portenschlagiana*	T23
Asterales	Campanulaceae	*Campanula poscharskyana*	Degen	*Campanula poscharskyana*	T23
Asterales	Campanulaceae	*Campanula punctata*	Lam.	*Campanula punctata*	T23
Asterales	Campanulaceae	*Campanula pyramidalis*	L.	*Campanula pyramidalis*	T23
Asterales	Campanulaceae	*Campanula rapunculus*	L.	*Campanula rapunculus*	T23
Asterales	Campanulaceae	*Campanula rotundifolia*	L.	*Campanula rotundifolia*	T23
Asterales	Campanulaceae	*Campanula trachelium*	L.	*Campanula trachelium*	T23
Rosales	Cannabaceae	*Cannabis sativa*	L.	*Cannabis sativa*	T23
Brassicales	Brassicaceae	*Capsella bursa-pastoris*	(L.) Medik.	*Capsella bursa-pastoris*	T23
Solanales	Solanaceae	*Capsicum annuum*	L.	*Capsicum annuum*	T23
Solanales	Solanaceae	*Capsicum chinense*	Jacq.	*Capsicum chinense*	T23
Brassicales	Brassicaceae	*Cardamine flexuosa*	With.	*Cardamine flexuosa*	T23
Brassicales	Brassicaceae	*Cardamine pratensis*	L.	*Cardamine pratensis*	T23
Asterales	Asteraceae	*Carduus acanthoides*	L.	*Carduus acanthoides*	T23
Asterales	Asteraceae	*Carduus crispus*	L.	*Carduus crispus*	T23
Asterales	Asteraceae	*Carduus nutans*	L.	*Carduus nutans*	T23
Asterales	Asteraceae	*Carduus pycnocephalus*	L.	*Carduus pycnocephalus*	T23
Asterales	Asteraceae	*Carduus tenuiflorus*	Curtis	*Carduus tenuiflorus*	T23 & this study
Poales	Cyperaceae	*Carex echinata*	Murray	*Carex echinata*	T23
Poales	Cyperaceae	*Carex hirta*	L.	*Carex hirta*	T23 & this study
Poales	Cyperaceae	*Carex nigra*	(L.) Reichard	*Carex nigra*	T23
Poales	Cyperaceae	*Carex panicea*	L.	*Carex panacea*	T23
Asterales	Asteraceae	*Carlina hispanica*	Lam.	*Carlina hispanica*	T23
Fagales	Betulaceae	*Carpinus orientalis*	Mill.	*Carpinus orientalis*	T23
Caryophyllales	Aizoaceae	*Carpobrotus chilensis*	(Molina) N.E.Br.	*Carpobrotus chilensis*	T23
Caryophyllales	Aizoaceae	*Carpobrotus edulis*	(L.) N.E.Br.	*Carpobrotus edulis*	T23
Asterales	Asteraceae	*Carthamus lanatus*	L.	*NA*	This study
Apiales	Apiaceae	*Carum carvi*	L.	*Carum carvi*	T23
Fagales	Juglandaceae	*Carya ovata*	(Mill.) K.Koch	*Carya ovata*	T23
Fagales	Fagaceae	*Castanea sativa*	Mill.	*Castanea sativa*	T23
Asterales	Asteraceae	*Catananche caerulea*	L.	*Catananche caerulea*	T23
Apiales	Apiaceae	*Caucalis platycarpos*	L.	*Caucalis platycarpos*	T23
Rosales	Rhamnaceae	*Ceanothus thyrsiflorus*	Eschw.	*Ceanothus thyrsiflorus*	T23
Rosales	Cannabaceae	*Celtis occidentalis*	L.	*Celtis occidentalis*	T23
Poales	Poaceae	*Cenchrus clandestinus*	(Hochst. ex Chiov.) Morrone	*Pennisetum clandestinum*	T23
Asterales	Asteraceae	*Centaurea aspera*	L.	*Centaurea aspera*	T23 & this study
Asterales	Asteraceae	*Centaurea cyanus*	L.	*Centaurea cyanus*	T23
Asterales	Asteraceae	*Centaurea jacea*	L.	*Centaurea jacea*	T23
Asterales	Asteraceae	*Centaurea macrocephala*	Muss.Puschk. ex Willd.	*Centaurea macrocephala*	T23
Asterales	Asteraceae	*Centaurea montana*	L.	*Centaurea montana*	T23
Asterales	Asteraceae	*Centaurea nigra*	L.	*Centaurea nigra*	T23 & this study
Asterales	Asteraceae	*Centaurea ornata*	Willd.	*Centaurea ornata*	T23
Asterales	Asteraceae	*Centaurea phrygia*	L.	*Centaurea phrygia*	T23
Asterales	Asteraceae	*Centaurea scabiosa*	L.	*Centaurea scabiosa*	T23
Asterales	Asteraceae	*Centaurea solstitialis*	L.	*Centaurea solstitialis*	T23
Gentianales	Gentianaceae	*Centaurium erythraea*	Rafn	*Centaurium erythraea*	T23
Dipsacales	Caprifoliaceae	*Centranthus calcitrapae*	(L.) Dufr.	*Centranthus calcitrapae*	T23
Dipsacales	Caprifoliaceae	*Centranthus ruber*	(L.) DC.	*Centranthus ruber*	T23
Dipsacales	Caprifoliaceae	*Cephalaria gigantea*	(Ledeb.) Bobrov	*Cephalaria gigantea*	T23
Caryophyllales	Caryophyllaceae	*Cerastium arvense*	L.	*Cerastium arvense*	T23
Caryophyllales	Caryophyllaceae	*Cerastium brachypetalum*	Desp. ex Pers.	*Cerastium brachypetalum*	T23
Caryophyllales	Caryophyllaceae	*Cerastium fontanum*	Baumg.	*Cerastium fontanum*	T23
Caryophyllales	Caryophyllaceae	*Cerastium*	Thuill.	*Cerastium*	T23

Order	Family	Name	Author	Name in Thompson et al. (2023)	Source
		glomeratum		*glomeratum*	
Caryophyllales	Caryophyllaceae	*Cerastium holosteoides*	Fr.	*Cerastium holosteoides*	T23
Saxifragales	Cercidiphyllaceae	*Cercidiphyllum japonicum*	Siebold & Zucc.	*Cercidiphyllum japonicum*	T23
Solanales	Solanaceae	*Cestrum elegans*	(Brongn. ex Neumann) Schltdl.	*Cestrum elegans*	T23
Rosales	Rosaceae	*Chaenomeles japonica*	(Thunb.) Lindl. ex Spach	*Chaenomeles japonica*	T23
Rosales	Rosaceae	*Chaenomeles speciosa*	(Sweet) Nakai	*Chaenomeles speciosa*	T23
Apiales	Apiaceae	*Chaerophyllum bulbosum*	L.	*Chaerophyllum bulbosum*	T23
Apiales	Apiaceae	*Chaerophyllum hirsutum*	L.	*Chaerophyllum hirsuitum*	T23
Apiales	Apiaceae	*Chaerophyllum temulum*	L.	*Chaerophyllum temulum*	T23
Myrtales	Onagraceae	*Chamaenerion angustifolium*	(L.) Scop.	*Chamaenerion angustifolium*	T23
Ranunculales	Papaveraceae	*Chelidonium majus*	L.	*Chelidonium majus*	T23
Caryophyllales	Amaranthaceae	*Chenopodium album*	L.	*Chenopodium album*	T23
Asparagales	Asparagaceae	*Chlorogalum pomeridianum*	(DC.) Kunth	*Chlorogalum pomeridianum*	T23
Asparagales	Asparagaceae	*Chlorophytum laxum*	R.Br.	*Chlorophytum laxum*	T23
Asterales	Asteraceae	*Chondrilla juncea*	L.	*Chondrilla juncea*	T23
Asterales	Asteraceae	*Chrysanthemum makinoi*	Matsum. & Nakai	*Chrysanthemum makinoi*	T23
Asterales	Asteraceae	*Chrysanthemum maximum*	L.	*Chrysanthemum maximum*	T23
Asterales	Asteraceae	*Chrysanthemum morifolium*	Chrysanthemum ×morifolium Ramat. ex Hemsl.	*Chrysanthemum x morifolium*	T23
Lamiales	Oleaceae	*Chrysojasminum humile*	(L.) Banfi	*Jasminum humile*	T23
Saxifragales	Saxifragaceae	*Chrysosplenium oppositifolium*	L.	*Chrysosplenium oppositifolium*	T23
Asterales	Asteraceae	*Cicerbita alpina*	(L.) Wallr.	*Cicerbita alpina*	T23
Asterales	Asteraceae	*Cichorium intybus*	L.	*Cichorium intybus*	T23 & this study
Myrtales	Onagraceae	*Circaea canadensis*	(L.) Hill	*Circaea canadensis*	T23
Asterales	Asteraceae	*Cirsium arvense*	(L.) Scop.	*Cirsium arvense*	T23
Asterales	Asteraceae	*Cirsium discolor*	(Muhl. ex Willd.) Spreng.	*Cirsium discolor*	T23
Asterales	Asteraceae	*Cirsium dissectum*	(L.) Hill	*Cirsium dissectum*	T23
Asterales	Asteraceae	*Cirsium heterophyllum*	(L.) Hill	*Cirsium heterophyllum*	T23
Asterales	Asteraceae	*Cirsium oleraceum*	(L.) Scop.	*Cirsium oleraceum*	T23
Asterales	Asteraceae	*Cirsium palustre*	(L.) Scop.	*Cirsium palustre*	T23
Asterales	Asteraceae	*Cirsium rivulare*	(Jacq.) All.	*Cirsium rivulare*	T23
Asterales	Asteraceae	*Cirsium vulgare*	(Savi) Ten.	*Cirsium lanceolatum*	T23
Asterales	Asteraceae	*Cirsium vulgare*	(Savi) Ten.	*Cirsium vulgare*	T23
Malvales	Cistaceae	*Cistus corbariensis*	Cistus ×corbariensis Pourr.	*Cistus corbariensis*	T23
Malvales	Cistaceae	*Cistus creticus*	L.	*Cistus creticus*	T23
Malvales	Cistaceae	*Cistus monspeliensis*	L.	*Cistus monspeliensis*	T23
Malvales	Cistaceae	*Cistus salviifolius*	L.	*Cistus salvifolius*	T23
Caryophyllales	Montiaceae	*Claytonia virginica*	L.	*Claytonia virginica*	T23
Ranunculales	Ranunculaceae	*Clematis cirrhosa*	L.	*Clematis cirrhosa*	T23
Ranunculales	Ranunculaceae	*Clematis montana*	Buch.-Ham. ex DC.	*Clematis montana*	T23
Ranunculales	Ranunculaceae	*Clematis tangutica*	(Maxim.) Korsh.	*Clematis tangutica*	T23
Ranunculales	Ranunculaceae	*Clematis vitalba*	L.	*Clematis vitalba*	T23
Caryophyllales	Aizoaceae	*Cleretum bellidiforme*	(Burm.fil.) G.D.Rowley	*Cleretum bellidiforme*	T23
Poales	Poaceae	*Coix lacryma-jobi*	L.	*Coix lachryma-jobi*	T23
Asterales	Asteraceae	*Coleostephus myconis*	(L.) Rchb.fil.	*Coleostephus myconis*	T23
Rosales	Rosaceae	*Comarum palustre*	L.	*Comarum palustre*	T23
Rosales	Rosaceae	*Comarum palustre*	L.	*Potentilla palustris*	T23
Commelinales	Commelinaceae	*Commelina diffusa*	Burm.f.	*Commelina diffusa*	T23
Fagales	Myricaceae	*Comptonia peregrina*	(L.) J.M.Coult.	*Comptonia peregrina*	T23
Apiales	Apiaceae	*Conium maculatum*	L.	*Conium maculatum*	T23
Apiales	Apiaceae	*Conopodium majus*	(Gouan) Loret	*Conopodium majus*	T23
Asparagales	Asparagaceae	*Convallaria majalis*	L.	*Convallaria majalis*	T23
Solanales	Convolvulaceae	*Convolvulus arvensis*	L.	*Convolvulus arvensis*	T23 & this study
Solanales	Convolvulaceae	*Convolvulus cneorum*	L.	*Convolvulus cneorum*	T23
Solanales	Convolvulaceae	*Convolvulus sericophyllus*	T.Anderson	*Convovulus acicularis*	T23
Asterales	Asteraceae	*Conyza sumatrensis*	(S.F.Blake) Pruski & G.Sancho, 2006	*Conyza sumatrensis*	T23
Gentianales	Rubiaceae	*Coprosma*	A.Gray	*Coprosma*	T23

Order	Family	Name	Author	Name in Thompson et al. (2023)	Source
		ernodeoides		*ernodeoides*	
Gentianales	Rubiaceae	*Coprosma rhynchocarpa*	A.Gray	*Coprosma rhynchocarpa*	T23
Asparagales	Asparagaceae	*Cordyline australis*	(G.Forst.) Endl.	*Cordyline australis*	T23
Asparagales	Asparagaceae	*Cordyline fruticosa*	(L.) A.Chev.	*Cordyline fruticosa*	T23
Apiales	Apiaceae	*Coriandrum sativum*	L.	*Coriandrum sativum*	T23
Cornales	Cornaceae	*Cornus alba*	L.	*Cornus alba*	T23
Cornales	Cornaceae	*Cornus canadensis*	L.	*Cornus canadensis*	T23
Cornales	Cornaceae	*Cornus racemosa*	Lam.	*Cornus racemosa*	T23
Cornales	Cornaceae	*Cornus sanguinea*	L.	*Cornus sanguinea*	T23
Cornales	Cornaceae	*Cornus suecica*	L.	*Cornus suecia*	T23
Fabales	Fabaceae	*Coronilla emerus*	Sm., 1832	*Coronilla emerus*	T23
Fabales	Fabaceae	*Coronilla scorpioides*	(L.) W.D.J.Koch	*Coronilla scorpioides*	T23
Fagales	Betulaceae	*Corylus avellana*	L.	*Corylus avellana*	T23
Poales	Poaceae	*Corynephorus canescens*	(L.) P.Beauv.	*Corynephorus canescens*	T23
Asterales	Asteraceae	*Cosmos atrosanguineus*	(Hook.) Voss	*Cosmos atrosanguineus*	T23
Asterales	Asteraceae	*Cota altissima*	(L.) Gay	*NA*	This study
Sapindales	Anacardiaceae	*Cotinus coggygria*	Scop.	*Cotinus coggygria*	T23
Rosales	Rosaceae	*Cotoneaster conspicuus*	Comber ex Marquand	*Cotoneaster conspicuus*	T23
Rosales	Rosaceae	*Cotoneaster franchetii*	Boiss.	*Cotoneaster franchetii*	T23
Rosales	Rosaceae	*Cotoneaster horizontalis*	Decne.	*Cotoneaster horizontalis*	T23
Rosales	Rosaceae	*Cotoneaster salicifolius*	Franch.	*Cotoneaster salicifolius*	T23
Saxifragales	Crassulaceae	*Crassula sarcocaulis*	Eckl. & Zeyh.	*Crassula sarcocaulis*	T23
Rosales	Rosaceae	*Crataegus monogyna*	Jacq.	*Crataegus monogyna*	T23
Asterales	Asteraceae	*Crepis biennis*	L.	*Crepis biennis*	T23
Asterales	Asteraceae	*Crepis bursifolia*	L.	*NA*	This study
Asterales	Asteraceae	*Crepis capillaris*	(L.) Wallr.	*Crepis capillaris*	T23
Asterales	Asteraceae	*Crepis foetida*	L.	*NA*	This study
Asterales	Asteraceae	*Crepis neglecta*	L.	*Crepis neglecta*	T23
Asterales	Asteraceae	*Crepis paludosa*	(L.) Moench	*Crepis paludosa*	T23
Asterales	Asteraceae	*Crepis sancta*	(L.) Bornm.	*NA*	This study
Asterales	Asteraceae	*Crepis vesicaria*	L.	*Crepis vesicaria*	T23 & this study
Oxalidales	Elaeocarpaceae	*Crinodendron hookerianum*	Gay	*Crinodendron hookerianum*	T23
Apiales	Apiaceae	*Crithmum maritimum*	L.	*Crithmum maritimum*	T23
Asparagales	Iridaceae	*Crocosmia crocosmiiflora*	Crocosmia ×crocosmiiflora (Lemoine) N.E.Br.	*Crocosmia × crocosmiiflora*	T23
Gentianales	Rubiaceae	*Crucianella maritima*	L.	*Crucianella maritima*	T23
Gentianales	Rubiaceae	*Cruciata laevipes*	Opiz	*Cruciata laevipes*	T23
Cucurbitales	Cucurbitaceae	*Cucumis sativus*	L.	*Cucumis sativus*	T23
Cucurbitales	Cucurbitaceae	*Cucurbita pepo*	L.	*Cucurbita pepo*	T23
Pinales	Cupressaceae	*Cupressus macrocarpa*	Hartw. ex Gordon	*Hesperocyparis macrocarpa*	T23
Rosales	Rosaceae	*Cydonia oblonga*	Mill.	*Cydonia oblonga*	T23
Asterales	Asteraceae	*Cynara cardunculus*	L.	*Cynara cardunculus*	T23
Asterales	Asteraceae	*Cynara scolymus*	L.	*Cynara scolymus*	T23
Poales	Poaceae	*Cynodon dactylon*	(L.) Pers.	*Cynodon dactylon*	T23
Poales	Poaceae	*Cynosurus cristatus*	L.	*Cynosurus cristatus*	T23
Poales	Cyperaceae	*Cyperus brevifolius*	(Rottb.) Hassk.	*Kyllinga brevifolia*	T23
Fabales	Fabaceae	*Cytisus kewensis*	Cytisus ×kewensis Bean	*Cytisus × kewensis*	T23
Fabales	Fabaceae	*Cytisus multiflorus*	(L'Hér.) Sweet	*Cytisus multiflora*	T23
Fabales	Fabaceae	*Cytisus praecox*	Cytisus ×praecox Bean	*Cytisus praecox*	T23
Fabales	Fabaceae	*Cytisus scoparius*	(L.) Link	*Cytisus scoparius*	T23
Poales	Poaceae	*Dactylis glomerata*	L.	*Dactylis glomerata*	T23 & this study
Asparagales	Orchidaceae	*Dactylorhiza maculata*	(L.) Soó	*Dactylorhiza fuchsii*	T23
Asparagales	Orchidaceae	*Dactylorhiza maculata*	(L.) Soó	*Dactylorhiza maculata*	T23
Malvales	Thymelaeaceae	*Daphne mezereum*	L.	*Daphne mezereum*	T23
Rosales	Rosaceae	*Dasiphora fruticosa*	(L.) Rydb.	*Dasiphora fruticosa*	T23
Poales	Poaceae	*Dasypyrum villosum*	(L.) Borbás	*Dasypyrum villosum*	T23
Apiales	Apiaceae	*Daucus carota*	L.	*Daucus carota*	T23 & this study

Order	Family	Name	Author	Name in Thompson et al. (2023)	Source
Poales	Poaceae	*Deschampsia cespitosa*	(L.) P.Beauv.	*Deschampsia cespitosa*	T23
Brassicales	Brassicaceae	*Descurainia sophia*	(L.) Webb ex Prantl	*Descurainia sophia*	T23
Caryophyllales	Caryophyllaceae	*Dianthus armeria*	L.	*Dianthus armeria*	T23
Caryophyllales	Caryophyllaceae	*Dianthus barbatus*	L.	*Dianthus barbatus*	T23
Caryophyllales	Caryophyllaceae	*Dianthus carthusianorum*	L.	*Dianthus carthusianorum*	T23
Caryophyllales	Caryophyllaceae	*Dianthus caryophyllus*	L.	*Dianthus caryophyllus*	T23
Caryophyllales	Caryophyllaceae	*Dianthus chinensis*	L.	*Dianthus chinensis*	T23
Caryophyllales	Caryophyllaceae	*Dianthus gratianopolitanus*	Vill.	*Dianthus gratianopolitanus*	T23
Caryophyllales	Caryophyllaceae	*Dianthus plumarius*	L.	*Dianthus plumarius*	T23
Poales	Poaceae	*Dichanthelium dichotomum*	(L.) Gould	*Dichanthelium dichotomum*	T23
Poales	Poaceae	*Digitaria horizontalis*	Willd.	*Digitaria horizontalis*	T23
Asterales	Asteraceae	*Dimorphotheca pluvialis*	(L.) Moench	*Dimorphotheca pluvialis*	T23
Lamiales	Phrymaceae	*Diplacus aurantiacus*	(Curtis) Jeps.	*Diplacus aurantiacus*	T23
Brassicales	Brassicaceae	*Diplotaxis tenuifolia*	(L.) DC.	*Diplotaxis tenuifolia*	T23
Dipsacales	Caprifoliaceae	*Dipsacus fullonum*	L.	*Dipsacus fullonum*	T23
Malvales	Thymelaeaceae	*Dirca palustris*	L.	*Dirca palustris*	T23
Asterales	Asteraceae	*Dittrichia viscosa*	(L.) Greuter	*Dittrichia viscosa*	T23
Lamiales	Lamiaceae	*Dracocephalum parviflorum*	Nutt.	*Dracocephalum parviflorum*	T23
Polypodiales	Dryopteridaceae	*Dryopteris carthusiana*	(Vill.) H.P.Fuchs	*Dryopteris carthusiana*	T23
Asterales	Asteraceae	*Dubautia scabra*	(DC.) D.D.Keck	*Dubautia scabra*	T23
Cucurbitales	Cucurbitaceae	*Ecballium elaterium*	(L.) A.Rich.	*Ecballium elaterium*	T23
Asterales	Asteraceae	*Echinacea pallida*	(Nutt.) Nutt.	*Echinacea pallida*	T23
Asterales	Asteraceae	*Echinops bannaticus*	Rochel ex Schrad.	*Echinops bannaticus*	T23
Asterales	Asteraceae	*Echinops ritro*	L.	*Echinops ritro*	T23
Asterales	Asteraceae	*Echinops sphaerocephalus*	L.	*Echinops sphaerocephalus*	T23
Boraginales	Boraginaceae	*Echium pininana*	Webb & Berthel.	*Echium pininana*	T23
Boraginales	Boraginaceae	*Echium plantagineum*	L.	*Echium plantagineum*	T23
Boraginales	Boraginaceae	*Echium vulgare*	L.	*Echium vulgare*	T23
Poales	Poaceae	*Elymus repens*	(L.) Gould	*Elymus repens*	T23 & this study
Myrtales	Onagraceae	*Epilobium alsinifolium*	Vill.	*Epilobium alsinifolium*	T23
Myrtales	Onagraceae	*Epilobium ciliatum*	Raf.	*Epilobium ciliatum*	T23
Myrtales	Onagraceae	*Epilobium hirsutum*	L.	*Epilobium hirsutum*	T23
Myrtales	Onagraceae	*Epilobium lanceolatum*	Sebast. & Mauri	*Epilobium lanceolatum*	T23
Myrtales	Onagraceae	*Epilobium montanum*	L.	*Epilobium montanum*	T23
Myrtales	Onagraceae	*Epilobium obscurum*	Schreb.	*Epilobium obscurum*	T23
Myrtales	Onagraceae	*Epilobium parviflorum*	Schreb.	*Epilobium parviflorum*	T23
Myrtales	Onagraceae	*Epilobium roseum*	(Schreb.) Schreb.	*Epilobium roseum*	T23
Myrtales	Onagraceae	*Epilobium tetragonum*	L.	*Epilobium tetragonum*	T23
Equisetales	Equisetaceae	*Equisetum arvense*	L.	*Equisetum arvense*	T23
Equisetales	Equisetaceae	*Equisetum sylvaticum*	L.	*Equisetum silvaticum*	T23
Ericales	Ericaceae	*Erica cinerea*	L.	*Erica cinerea*	T23
Ericales	Ericaceae	*Erica darleyensis*	Erica ×darleyensis Bean	*Erica x darleyensis*	T23
Ericales	Ericaceae	*Erica scoparia*	L.	*Erica scoparia*	T23
Ericales	Ericaceae	*Erica tetralix*	L.	*Erica tetralix*	T23
Asterales	Asteraceae	*Erigeron annuus*	(L.) Pers.	*Erigeron annuus*	T23
Asterales	Asteraceae	*Erigeron bonariensis*	L.	*Erigeron bonariensis*	T23
Asterales	Asteraceae	*Erigeron canadensis*	L.	*Erigeron canadensis*	T23
Asterales	Asteraceae	*Erigeron glaucus*	Ker Gawl.	*Erigeron glaucus*	T23
Asterales	Asteraceae	*Erigeron karvinskianus*	DC.	*Erigeron karvinskianus*	T23
Asterales	Asteraceae	*Erigeron philadelphicus*	L.	*Erigeron philadelphicus*	T23
Asterales	Asteraceae	*Erigeron strigosus*	Muhl. ex Willd.	*Erigeron strigosus*	T23
Asterales	Asteraceae	*Erigeron sumatrensis*	Retz.	*Erigeron sumatrensis*	T23
Ranunculales	Ranunculaceae	*Eriocapitella hupehensis*	(Lemoine) Christenh. & Byng	*Eriocapitella hupehensis*	T23
Geraniales	Geraniaceae	*Erodium botrys*	(Cav.) Bertol.	*NA*	This study
Geraniales	Geraniaceae	*Erodium ciconium*	(L.) L'Hér.	*NA*	This study
Geraniales	Geraniaceae	*Erodium cicutarium*	(L.) L'Hér.	*Erodium cicutarium*	T23 & this study

Order	Family	Name	Author	Name in Thompson et al. (2023)	Source
Brassicales	Brassicaceae	*Eruca vesicaria*	(L.) Cav.	*Eruca vesicaria*	T23
Apiales	Apiaceae	*Eryngium campestre*	L.	*Eryngium campestre*	T23 & this study
Apiales	Apiaceae	*Eryngium maritimum*	L.	*Eryngium maritimum*	T23
Apiales	Apiaceae	*Eryngium planum*	L.	*Eryngium planum*	T23
Brassicales	Brassicaceae	*Erysimum cheiranthoides*	L.	*Erysimum cheiranthoides*	T23
Brassicales	Brassicaceae	*Erysimum cheiri*	(L.) Crantz	*Erysimum cheiri*	T23
Lamiales	Phrymaceae	*Erythranthe guttata*	(DC.) G.L.Nesom	*Erythranthe guttata*	T23
Lamiales	Phrymaceae	*Erythranthe lutea*	(L.) G.L.Nesom	*Erythranthe lutea*	T23
Ranunculales	Papaveraceae	*Eschscholzia californica*	Cham.	*Eschscholzia californica*	T23
Myrtales	Myrtaceae	*Eucalyptus amygdalina*	Labill.	*Eucalyptus amygdalina*	T23
Asparagales	Asparagaceae	*Eucomis comosa*	(Houtt.) H.R.Wehrh.	*Eucomis comosa*	T23
Celastrales	Celastraceae	*Euonymus europaeus*	L.	*Euonymus europaeus*	T23
Celastrales	Celastraceae	*Euonymus fortunei*	(Turcz.) Hand.-Mazz.	*Euonymus fortunei*	T23
Celastrales	Celastraceae	*Euonymus japonicus*	Thunb.	*Euonymus japonicus*	T23
Asterales	Asteraceae	*Eupatorium cannabinum*	L.	*Eupatorium cannabinum*	T23
Malpighiales	Euphorbiaceae	*Euphorbia characias*	L.	*Euphorbia characias*	T23
Malpighiales	Euphorbiaceae	*Euphorbia cyparissias*	L.	*Euphorbia cyparissias*	T23
Malpighiales	Euphorbiaceae	*Euphorbia dulcis*	L.	*Euphorbia dulcis*	T23
Malpighiales	Euphorbiaceae	*Euphorbia griffithii*	Hook.f.	*Euphorbia griffithii*	T23
Malpighiales	Euphorbiaceae	*Euphorbia helioscopia*	L.	*NA*	This study
Malpighiales	Euphorbiaceae	*Euphorbia purpurea*	(Raf.) Fernald	*Euphorbia purpurea*	T23
Malpighiales	Euphorbiaceae	*Euphorbia serrata*	L.	*Euphorbia serrata*	T23
Malpighiales	Euphorbiaceae	*Euphorbia terracina*	L.	*Euphorbia terracina*	T23
Lamiales	Orobanchaceae	*Euphrasia officinalis*	L.	*Euphrasia officinalis*	T23
Asterales	Asteraceae	*Eurybia divaricata*	(L.) G.L.Nesom	*Eurybia divaricata*	T23
Asterales	Asteraceae	*Euthamia graminifolia*	(L.) Nutt.	*Euthenia graminifolia*	T23
Asterales	Asteraceae	*Eutrochium maculatum*	(L.) E.E.Lamont	*Eutrochium maculatum*	T23
Caryophyllales	Polygonaceae	*Fagopyrum esculentum*	Moench	*Fagopyrum esculentum*	T23
Fagales	Fagaceae	*Fagus sylvatica*	L.	*Fagus sylvatica*	T23
Apiales	Apiaceae	*Falcaria vulgaris*	Bernh.	*Falcaria vulgaris*	T23 & this study
Caryophyllales	Polygonaceae	*Fallopia baldschuanica*	(Regel) Holub	*Fallopia baldschuanica*	T23
Caryophyllales	Polygonaceae	*Fallopia convolvulus*	(L.) Á.Löve	*Fallopia convovulus*	T23
Poales	Bromeliaceae	*Fascicularia bicolor*	(Ruiz & Pav.) Mez	*Fascicularia bicolor*	T23
Asterales	Asteraceae	*Felicia petiolata*	(Harv.) N.E.Br.	*Felicia petiolata*	T23
Poales	Poaceae	*Festuca glauca*	Vill.	*Festuca glauca*	T23
Poales	Poaceae	*Festuca ovina*	L.	*Festuca ovina*	T23
Poales	Poaceae	*Festuca rubra*	L.	*Festuca rubra*	T23
Rosales	Moraceae	*Ficus carica*	L.	*Ficus carica*	T23
Rosales	Rosaceae	*Filipendula ulmaria*	(L.) Maxim.	*Filipendula ulmaria*	T23
Rosales	Rosaceae	*Filipendula vulgaris*	Moench	*Filipendula vulgaris*	T23 & this study
Apiales	Apiaceae	*Foeniculum vulgare*	Mill.	*Foeniculum vulgare*	T23 & this study
Lamiales	Oleaceae	*Forsythia intermedia*	Forsythia ×intermedia Zabel	*Forsythia × intermedia*	T23
Lamiales	Oleaceae	*Forsythia suspensa*	(Thunb.) Vahl	*Forsythia suspensa*	T23
Rosales	Rosaceae	*Fragaria ananassa*	Fragaria ×ananassa (Weston) Rozier	*Fragaria x ananassa*	T23
Rosales	Rosaceae	*Fragaria chiloensis*	(L.) Mill.	*Fragaria chiloensis*	T23
Rosales	Rosaceae	*Fragaria moschata*	Duchesne	*Fragaria moschata*	T23
Rosales	Rosaceae	*Fragaria vesca*	L.	*Fragaria vesca*	T23
Rosales	Rosaceae	*Fragaria virginiana*	Duchesne	*Fragaria virginiana*	T23
Lamiales	Oleaceae	*Fraxinus excelsior*	L.	*Fraxinus excelsior*	T23
Lamiales	Oleaceae	*Fraxinus ornus*	L.	*Fraxinus ornus*	T23
Lamiales	Oleaceae	*Fraxinus pennsylvanica*	Marshall	*Fraxinus pennsylvanica*	T23
Myrtales	Onagraceae	*Fuchsia arborescens*	La Llave, 1832	*Fuchsia arborescens*	T23
Myrtales	Onagraceae	*Fuchsia magellanica*	Lam.	*Fuchsia magellanica*	T23
Myrtales	Onagraceae	*Fuchsia microphylla*	Kunth	*Fuchsia microphylla*	T23
Myrtales	Onagraceae	*Fuchsia procumbens*	R.Cunn.	*Fuchsia procumbens*	T23
Myrtales	Onagraceae	*Fuchsia triphylla*	L.	*Fuchsia triphylla*	T23
Asterales	Asteraceae	*Galactites tomentosa*	Moench	*Galactites tomentosa*	T23 & this study

Order	Family	Name	Author	Name in Thompson et al. (2023)	Source
Fabales	Fabaceae	*Galega officinalis*	L.	*Galega officinalis*	T23
Lamiales	Lamiaceae	*Galeopsis bifida*	Boenn.	*Galeopsis bifida*	T23
Lamiales	Lamiaceae	*Galeopsis speciosa*	Mill.	*Galeopsis speciosa*	T23
Lamiales	Lamiaceae	*Galeopsis tetrahit*	L.	*Galeopsis tetrahit*	T23
Asterales	Asteraceae	*Galinsoga parviflora*	Cav.	*Galinsoga parviflora*	T23
Asterales	Asteraceae	*Galinsoga quadriradiata*	Ruiz & Pav.	*Galinsoga ciliata*	T23
Gentianales	Rubiaceae	*Galium album*	Mill.	*NA*	This study
Gentianales	Rubiaceae	*Galium aparine*	L.	*Galium aparine*	T23
Gentianales	Rubiaceae	*Galium asprellum*	Michx.	*Galium asprellum*	T23
Gentianales	Rubiaceae	*Galium boreale*	L.	*Galium boreale*	T23
Gentianales	Rubiaceae	*Galium mollugo*	L.	*Galium mollugo*	T23 & this study
Gentianales	Rubiaceae	*Galium palustre*	L.	*Galium palustre*	T23 & this study
Gentianales	Rubiaceae	*Galium saxatile*	L.	*Galium saxatile*	T23
Gentianales	Rubiaceae	*Galium sylvaticum*	L.	*Galium sylvaticum*	T23
Gentianales	Rubiaceae	*Galium uliginosum*	L.	*Galium uliginosum*	T23
Gentianales	Rubiaceae	*Galium verum*	L.	*Galium verum*	T23 & this study
Asterales	Asteraceae	*Gamochaeta purpurea*	(L.) Cabrera	*Gamochaeta purpurea*	T23
Asterales	Asteraceae	*Gazania rigens*	(L.) Gaertn.	*Gazania rigens*	T23
Gentianales	Gentianaceae	*Gentiana asclepiadea*	L.	*Gentiana asclepiadea*	T23
Geraniales	Geraniaceae	*Geranium carolinianum*	L.	*Geranium carolinianum*	T23
Geraniales	Geraniaceae	*Geranium cinereum*	Cav.	*Geranium cinereum*	T23
Geraniales	Geraniaceae	*Geranium dissectum*	L.	*Geranium dissectum*	T23 & this study
Geraniales	Geraniaceae	*Geranium macrorrhizum*	L.	*Geranium macrorrhizum*	T23
Geraniales	Geraniaceae	*Geranium maculatum*	L.	*Geranium maculatum*	T23
Geraniales	Geraniaceae	*Geranium molle*	L.	*Geranium molle*	T23 & this study
Geraniales	Geraniaceae	*Geranium platypetalum*	Fisch. & C.A.Mey.	*Geranium platypetalum*	T23
Geraniales	Geraniaceae	*Geranium pratense*	L.	*Geranium pratense*	T23
Geraniales	Geraniaceae	*Geranium pusillum*	L.	*Geranium pusillum*	T23
Geraniales	Geraniaceae	*Geranium pyrenaicum*	Burm.f.	*Geranium pyrenaicum*	T23
Geraniales	Geraniaceae	*Geranium robertianum*	L.	*Geranium robertianum*	T23
Geraniales	Geraniaceae	*Geranium sanguineum*	L.	*Geranium sanguineum*	T23
Geraniales	Geraniaceae	*Geranium sylvaticum*	L.	*Geranium sylvaticum*	T23
Rosales	Rosaceae	*Geum aleppicum*	Jacq.	*Geum aleppicum*	T23
Rosales	Rosaceae	*Geum coccineum*	Sibth. & Sm.	*Geum coccineum*	T23
Rosales	Rosaceae	*Geum quellyon*	Sweet	*Geum quellyon*	T23
Rosales	Rosaceae	*Geum rivale*	L.	*Geum rivale*	T23
Rosales	Rosaceae	*Geum urbanum*	L.	*Geum ubanum*	T23
Asparagales	Iridaceae	*Gladiolus communis*	L.	*Gladiolus communis*	T23
Boraginales	Boraginaceae	*Glandora diffusa*	(Lag.) D.C.Thomas	*Glandora diffusa*	T23
Asterales	Asteraceae	*Glebionis carinata*	(Schousb.) Tzvelev	*Chrysanthemum carinatum*	T23
Asterales	Asteraceae	*Glebionis coronaria*	(L.) Tzvelev	*Glebionis coronaria*	T23
Lamiales	Lamiaceae	*Glechoma hederacea*	L.	*Glechoma hederacea*	T23 & this study
Fabales	Fabaceae	*Glycine max*	(L.) Merr.	*Glycine max*	T23
Asterales	Asteraceae	*Gnaphalium sylvaticum*	Sm.	*Gnaphalium sylvaticum*	T23
Malvales	Malvaceae	*Gossypium hirsutum*	L.	*Gossypium hirsutum*	T23
Proteales	Proteaceae	*Grevillea rosmarinifolia*	A.Cunn.	*Grevillea rosmarinifolia*	T23
Ranunculales	Ranunculaceae	*Halerpestes cymbalaria*	(Pursh) Greene	*Ranunculus cymbalaria*	T23
Apiales	Araliaceae	*Hedera canariensis*	Willd.	*Hedera canariensis*	T23
Apiales	Araliaceae	*Hedera colchica*	(K.Koch) K.Koch	*Hedera colchica*	T23
Apiales	Araliaceae	*Hedera helix*	L.	*Hedera helix*	T23
Zingiberales	Zingiberaceae	*Hedychium coronarium*	J.Koenig	*Hedychium coronarium*	T23
Asterales	Asteraceae	*Hedypnois cretica*	(L.) Dum.Cours.	*Hedypnois cretica*	T23
Malvales	Cistaceae	*Helianthemum nummularium*	(L.) Mill.	*Helianthemum nummularium*	T23
Asterales	Asteraceae	*Helianthus annuus*	L.	*Helianthus annuus*	T23
Asterales	Asteraceae	*Helianthus giganteus*	L.	*Helicanthus giganteus*	T23

Order	Family	Name	Author	Name in Thompson et al. (2023)	Source
Asterales	Asteraceae	*Helianthus tuberosus*	L.	*Helianthus tuberosus*	T23
Asterales	Asteraceae	*Helichrysum italicum*	(Roth) G.Don	*Helichrysum italicum*	T23
Poales	Poaceae	*Helictotrichon pratense*	(L.) Besser	*Helictotrichon pratense*	T23
Asterales	Asteraceae	*Heliopsis helianthoides*	(L.) Sweet	*Heliopsis scabra*	T23
Boraginales	Heliotropiaceae	*Heliotropium arborescens*	L.	*Heliotropium arborescens*	T23
Asterales	Asteraceae	*Helminthotheca echioides*	(L) Holub	*Helminthoteca echioides*	T23 & this study
Apiales	Apiaceae	*Helosciadium nodiflorum*	(L.) W.D.J.Koch	*Apium nodiflorum*	T23
Asparagales	Asphodelaceae	*Hemerocallis fulva*	(L.) L.	*Hemerocallis fulva*	T23
Asparagales	Asphodelaceae	*Hemerocallis lilioasphodelus*	L.	*Hemerocallis lilioasphodelus*	T23
Asterales	Asteraceae	*Hemizonia congesta*	DC.	*Hemizonia congesta*	T23
Apiales	Apiaceae	*Heracleum mantegazzianum*	Sommier & Levier	*Heracleum mantegazzianum*	T23
Apiales	Apiaceae	*Heracleum maximum*	W.Bartram	*Heracleum maximum*	T23
Apiales	Apiaceae	*Heracleum sphondylium*	L.	*Heracleum sphondylium*	T23
Asparagales	Iridaceae	*Hesperantha coccinea*	(Backh. & Harv.) Goldblatt & J.C.Manning	*Hesperantha coccinea*	T23
Brassicales	Brassicaceae	*Hesperis matronalis*	L.	*Hesperis matronalis*	T23
Saxifragales	Saxifragaceae	*Heuchera sanguinea*	Engelm.	*Heuchera sanguinea*	T23
Malvales	Malvaceae	*Hibiscus syriacus*	L.	*Hibiscus syriacus*	T23
Asterales	Asteraceae	*Hieracium caespitosum*	Dahlst., 1894	*Hieracium caespitosum*	T23
Asterales	Asteraceae	*Hieracium lepidulum*	Stenstr., 1889	*Hieracium lepidulum*	T23
Asterales	Asteraceae	*Hieracium maculatum*	Schrank	*Hieracium maculatum*	T23
Asterales	Asteraceae	*Hieracium murorum*	L.	*Hieracium murorum*	T23
Asterales	Asteraceae	*Hieracium pilosum*	Schleich. ex Froel.	*Hieracium pilosella*	T23
Asterales	Asteraceae	*Hieracium praealtum*	Rchb.	*Hieracium praealtum*	T23
Asterales	Asteraceae	*Hieracium tridentatum*	Fr.	*Hieracium tridentatum*	T23
Asterales	Asteraceae	*Hieracium umbellatum*	L.	*Hieracium umbellatum*	T23
Rosales	Elaeagnaceae	*Hippophae rhamnoides*	L.	*Hippophae rhamnoides*	T23
Lamiales	Plantaginaceae	*Hippuris vulgaris*	L.	*Hippuris vulgaris*	T23
Poales	Poaceae	*Holcus lanatus*	L.	*Holcus lanatus*	T23 & this study
Poales	Poaceae	*Holcus mollis*	L.	*Holcus mollis*	T23
Poales	Poaceae	*Hordeum brachyantherum*	Nevski	*Hordeum brachyantherum*	T23
Poales	Poaceae	*Hordeum bulbosum*	L.	*Hordeum bulbosum*	T23
Poales	Poaceae	*Hordeum murinum*	L.	*Hordeum murinum*	T23
Poales	Poaceae	*Hordeum vulgare*	L.	*Hordeum vulgare*	T23
Piperales	Saururaceae	*Houttuynia cordata*	Thunb.	*Houttuynia cordata*	T23
Rosales	Cannabaceae	*Humulus lupulus*	L.	*Humulus lupulus*	T23
Asparagales	Asparagaceae	*Hyacinthoides hispanica*	(Mill.) Rothm.	*Hyacinthoides hispanica*	T23
Asparagales	Asparagaceae	*Hyacinthoides non-scripta*	(L.) Chouard ex Rothm.	*Hyacinthoides non-scripta*	T23
Cornales	Hydrangeaceae	*Hydrangea macrophylla*	(Thunb.) Ser.	*Hydrangea macrophylla*	T23
Cornales	Hydrangeaceae	*Hydrangea paniculata*	Siebold	*Hydrangea paniculata*	T23
Cornales	Hydrangeaceae	*Hydrangea petiolaris*	Siebold & Zucc.	*Hydrangea petiolaris*	T23
Saxifragales	Crassulaceae	*Hylotelephium spectabile*	(Boreau) H.Ohba	*Hylotelephium spectabile*	T23
Malpighiales	Hypericaceae	*Hypericum androsaemum*	L.	*Hypericum androsaemum*	T23
Malpighiales	Hypericaceae	*Hypericum calycinum*	L.	*Hypericum calycinum*	T23
Malpighiales	Hypericaceae	*Hypericum hirsutum*	L.	*Hypericum hirsutum*	T23
Malpighiales	Hypericaceae	*Hypericum maculatum*	Crantz	*Hypericum maculatum*	T23
Malpighiales	Hypericaceae	*Hypericum moserianum*	Hypericum ×moserianum André	*Hypericum moserianum*	T23
Malpighiales	Hypericaceae	*Hypericum olympicum*	L.	*Hypericum olympicum*	T23
Malpighiales	Hypericaceae	*Hypericum perforatum*	L.	*Hypericum perforatum*	T23 & this study
Malpighiales	Hypericaceae	*Hypericum pulchrum*	L.	*Hypericum pulchrum*	T23
Malpighiales	Hypericaceae	*Hypericum tetrapterum*	Fr.	*Hypericum tetrapterum*	T23
Asterales	Asteraceae	*Hypochaeris glabra*	L.	*Hypochaeris glabra*	T23

Order	Family	Name	Author	Name in Thompson et al. (2023)	Source
Asterales	Asteraceae	*Hypochaeris radicata*	L.	*Hypochaeris radicata*	T23 & this study
Lamiales	Lamiaceae	*Hyssopus officinalis*	L.	*Hyssopus officinalis*	T23
Brassicales	Brassicaceae	*Iberis sempervirens*	L.	*Iberis sempervirens*	T23
Aquifoliales	Aquifoliaceae	*Ilex anomala*	Hook. & Arn.	*Ilex anomala*	T23
Aquifoliales	Aquifoliaceae	*Ilex verticillata*	(L.) A.Gray	*Ilex verticillata*	T23
Ericales	Balsaminaceae	*Impatiens capensis*	Meerb.	*Impatiens capensis*	T23
Ericales	Balsaminaceae	*Impatiens glandulifera*	Royle	*Impatiens glandulifera*	T23
Ericales	Balsaminaceae	*Impatiens noli-tangere*	L.	*Impatiens noli-tangere*	T23
Solanales	Convolvulaceae	*Ipomoea batatas*	(L.) Lam.	*Ipomea batatas*	T23
Solanales	Convolvulaceae	*Ipomoea indica*	(Burm.) Merr.	*Ipomoea indica*	T23
Solanales	Convolvulaceae	*Ipomoea muricata*	(L.) Jacq.	*Ipomoea muricata*	T23
Asparagales	Iridaceae	*Iris germanica*	Iris ×germanica L.	*Iris germanica*	T23
Asparagales	Iridaceae	*Iris pseudacorus*	L.	*Iris pseudacorus*	T23
Asparagales	Iridaceae	*Iris sibirica*	L.	*Iris sibirica*	T23
Asparagales	Iridaceae	*Iris versicolor*	L.	*Iris versicolor*	T23
Asterales	Asteraceae	*Jacobaea aquatica*	(Hill) G.Gaertn., B.Mey. & Scherb.	*Jacobaea aquatica*	T23
Asterales	Asteraceae	*Jacobaea erratica*	(Bertol.) Fourr.	*NA*	This study
Asterales	Asteraceae	*Jacobaea maritima*	(L.) Pelser & Meijden	*Jacobaea maritima*	T23
Asterales	Asteraceae	*Jacobaea paludosa*	(L.) G.Gaertn., B.Mey. & Scherb.	*Jacobaea paludosa*	T23
Asterales	Asteraceae	*Jacobaea vulgaris*	Gaertn.	*Jacobaea vulgaris*	T23 & this study
Asterales	Campanulaceae	*Jasione maritima*	(Duby) L.M.Dufour ex Merino	*Jasione maritima*	T23
Lamiales	Oleaceae	*Jasminum nudiflorum*	Lindl.	*Jasminum nudiflorum*	T23
Lamiales	Oleaceae	*Jasminum officinale*	L.	*Jasminum officinale*	T23
Fagales	Juglandaceae	*Juglans nigra*	L.	*Juglans nigra*	T23
Fagales	Juglandaceae	*Juglans regia*	L.	*Juglans regia*	T23
Poales	Juncaceae	*Juncus acutiflorus*	Ehrh. ex Hoffm.	*Juncus acutiflorus*	T23
Poales	Juncaceae	*Juncus acutus*	L.	*Juncus acutus*	T23
Poales	Juncaceae	*Juncus effusus*	L.	*Juncus effusus*	T23
Poales	Juncaceae	*Juncus gerardi*	Loisel.	*Juncus gerardii*	T23
Poales	Juncaceae	*Juncus inflexus*	L.	*Juncus inflexus*	T23
Poales	Juncaceae	*Juncus squarrosus*	L.	*Juncus squarrosus*	T23
Pinales	Cupressaceae	*Juniperus communis*	L.	*Juniperus communis*	T23
Pinales	Cupressaceae	*Juniperus oxycedrus*	L.	*Juniperus oxycedrus*	T23
Pinales	Cupressaceae	*Juniperus virginiana*	L.	*Juniperus virginiana*	T23
Rosales	Rosaceae	*Kerria japonica*	(L.) DC.	*Kerria japonica*	T23
Dipsacales	Caprifoliaceae	*Knautia arvensis*	(L.) Coult.	*Knautia arvensis*	T23
Dipsacales	Caprifoliaceae	*Knautia drymeia*	Heuff.	*Knautia sylvatica*	T23
Dipsacales	Caprifoliaceae	*Knautia macedonica*	Griseb.	*Knautia macedonica*	T23
Asparagales	Asphodelaceae	*Kniphofia galpinii*	Baker	*Kniphofia galpinii*	T23
Malvales	Malvaceae	*Kokia drynarioides*	(Seem.) Lewton	*Kokia drynarioides*	T23
Dipsacales	Caprifoliaceae	*Kolkwitzia amabilis*	Graebn.	*Linnaea amabilis*	T23
Asterales	Asteraceae	*Lactuca sativa*	L.	*Lactuca sativa*	T23
Asterales	Asteraceae	*Lactuca serriola*	L.	*Lactuca serriola*	T23
Asterales	Asteraceae	*Lactuca virosa*	L.	*Lactuca virosa*	T23
Poales	Poaceae	*Lagurus ovatus*	L.	*Lagurus ovatus*	T23
Proteales	Proteaceae	*Lambertia rariflora*	Meisn.	*Lambertia rariflora*	T23
Lamiales	Lamiaceae	*Lamium album*	L.	*Lamium album*	T23
Lamiales	Lamiaceae	*Lamium amplexicaule*	L.	*Lamium amplexicaule*	T23
Lamiales	Lamiaceae	*Lamium galeobdolon*	(L.) L.	*Lamium galeobdolon*	T23
Lamiales	Lamiaceae	*Lamium maculatum*	(L.) L.	*Lamium maculatum*	T23
Asterales	Asteraceae	*Lapsana communis*	L.	*Lapsana communis*	T23
Apiales	Apiaceae	*Laserpitium latifolium*	L.	*Laserpitium latifolium*	T23
Fabales	Fabaceae	*Lathyrus japonicus*	Willd.	*Lathyrus japonicus*	T23
Fabales	Fabaceae	*Lathyrus latifolius*	L.	*Lathyrus latifolius*	T23
Fabales	Fabaceae	*Lathyrus linifolius*	(Reichard) Bässler	*Lathyrus linifolius*	T23
Fabales	Fabaceae	*Lathyrus nissolia*	L.	*Lathyrus nissolia*	T23
Fabales	Fabaceae	*Lathyrus ochrus*	(L.) DC.	*Lathyrus ochrus*	T23
Fabales	Fabaceae	*Lathyrus odoratus*	L.	*Lathyrus odoratus*	T23
Fabales	Fabaceae	*Lathyrus oleraceus*	Lam.	*Pisum sativum*	T23
Fabales	Fabaceae	*Lathyrus palustris*	L.	*Lathyrus palustris*	T23
Fabales	Fabaceae	*Lathyrus pratensis*	L.	*Lathyrus pratensis*	T23 & this study
Fabales	Fabaceae	*Lathyrus sativus*	L.	*Lathyrus sativa*	T23
Fabales	Fabaceae	*Lathyrus sylvestris*	L.	*Lathyrus silvestris*	T23
Fabales	Fabaceae	*Lathyrus vernus*	(L.) Bernh.	*Lathyrus vernus*	T23
Laurales	Lauraceae	*Laurus nobilis*	L.	*Laurus nobilis*	T23
Lamiales	Lamiaceae	*Lavandula*	Mill.	*Lavandula*	T23

Order	Family	Name	Author	Name in Thompson et al. (2023)	Source
		angustifolia		*angustifolia*	
Lamiales	Lamiaceae	*Lavandula intermedia*	Lavandula ×intermedia Emeric ex Loisel.	*Lavandula x intermedia*	T23
Lamiales	Lamiaceae	*Lavandula stoechas*	L.	*Lavandula stoechas*	T23
Asterales	Asteraceae	*Leontodon hispidus*	L.	*Leontodon hispidus*	T23
Asterales	Asteraceae	*Leontodon taraxacoides*	Lacaita, 1918	*Leontodon taraxacoides*	T23
Brassicales	Brassicaceae	*Lepidium campestre*	(L.) W.T.Aiton	*Lepidium campestre*	T23
Brassicales	Brassicaceae	*Lepidium didymum*	L.	*Lepidium didymum*	T23
Brassicales	Brassicaceae	*Lepidium draba*	L.	*Lepidium draba*	T23
Brassicales	Brassicaceae	*Lepidium ruderale*	L.	*Lepidium ruderale*	T23
Brassicales	Brassicaceae	*Lepidium virginicum*	L.	*Lepidium virginicum*	T23
Ericales	Ericaceae	*Leptecophylla tameiameiae*	(Cham. & Schltdl.) C.M.Weiller	*Leptecophylla tameiameiae*	T23
Myrtales	Myrtaceae	*Leptospermum laevigatum*	(Gaertn.) F.Muell.	*Leptospermum laevigatum*	T23
Myrtales	Myrtaceae	*Leptospermum scoparium*	Forst.	*Leptospermum scoparium*	T23
Asterales	Asteraceae	*Leucanthemum ircutianum*	(Turcz.) DC.	*NA*	This study
Asterales	Asteraceae	*Leucanthemum superbum*	Leucanthemum ×superbum (Bergmans ex J.W.Ingram) D.H.Kent	*Leucanthemum x superbum*	T23
Asterales	Asteraceae	*Leucanthemum sylvaticum*	(Hoffmanns. & Link) Nyman	*Leucanthemum merinoi*	T23
Asterales	Asteraceae	*Leucanthemum vulgare*	Lam.	*Leucanthemum vulgare*	T23
Asterales	Asteraceae	*Leuzea carthamoides*	(Willd.) DC.	*Rhaponticum carthamoides*	T23
Asterales	Asteraceae	*Leuzea pusilla*	Spreng.	*Rhaponticum scariosum*	T23
Apiales	Apiaceae	*Levisticum officinale*	W.D.J.Koch	*Levisticum officinale*	T23
Poales	Poaceae	*Leymus arenarius*	(L.) Hochst.	*Leymus arenarius*	T23
Asparagales	Iridaceae	*Libertia ixioides*	(G.Forst.) Spreng.	*Libertia ixioides*	T23
Apiales	Apiaceae	*Ligusticum scoticum*	Lour., 1790	*Ligusticum scoticum*	T23
Lamiales	Oleaceae	*Ligustrum ovalifolium*	Hassk.	*Ligustrum ovalifolium*	T23
Lamiales	Oleaceae	*Ligustrum vulgare*	L.	*Ligustrum vulgare*	T23
Liliales	Liliaceae	*Lilium martagon*	L.	*Lilium martagon*	T23
Lamiales	Plantaginaceae	*Linaria purpurea*	(L.) Mill.	*Linaria purpurea*	T23
Lamiales	Plantaginaceae	*Linaria vulgaris*	Mill.	*Linaria vulgaris*	T23
Malpighiales	Linaceae	*Linum catharticum*	L.	*Linum catharticum*	T23
Malpighiales	Linaceae	*Linum usitatissimum*	L.	*Linum usitatissimum*	T23
Saxifragales	Altingiaceae	*Liquidambar styraciflua*	L.	*Liquidambar styraciflua*	T23
Boraginales	Boraginaceae	*Lithospermum caroliniense*	(J.F.Gmel.) Mac Mill.	*Lithospermum croceum*	T23
Boraginales	Boraginaceae	*Lithospermum officinale*	L.	*Lithospermum officinale*	T23
Asterales	Campanulaceae	*Lobelia cardinalis*	L.	*Lobelia cardinalis*	T23
Asterales	Campanulaceae	*Lobelia gibbosa*	Labill.	*Lobelia gibbosa*	T23
Asterales	Asteraceae	*Logfia gallica*	(L.) Dumort.	*Logfia gallica*	T23
Poales	Poaceae	*Lolium arundinaceum*	(Schreb.) Darbysh.	*Festuca arundinacea*	T23 & this study
Poales	Poaceae	*Lolium multiflorum*	Lam.	*Lolium multiflorum*	T23
Poales	Poaceae	*Lolium perenne*	L.	*Lolium perenne*	T23
Poales	Poaceae	*Lolium rigidum*	Gaudin	*NA*	This study
Dipsacales	Caprifoliaceae	*Lonicera caerulea*	L.	*Lonicera caerulea*	T23
Dipsacales	Caprifoliaceae	*Lonicera caprifolium*	L.	*Lonicera caprifolium*	T23
Dipsacales	Caprifoliaceae	*Lonicera etrusca*	Santi	*Lonicera etrusca*	T23
Dipsacales	Caprifoliaceae	*Lonicera japonica*	Thunb.	*Lonicera japonica*	T23
Dipsacales	Caprifoliaceae	*Lonicera periclymenum*	L.	*Lonicera periclymenum*	T23
Dipsacales	Caprifoliaceae	*Lonicera pileata*	Oliv.	*Lonicera nitida*	T23
Dipsacales	Caprifoliaceae	*Lonicera pileata*	Oliv.	*Lonicera pileata*	T23
Asterales	Asteraceae	*Lophiolepis eriophora*	(L.) Del Guacchio, Bures, Iamonico & P.Caputo	*Cirsium eriophorum*	T23
Fabales	Fabaceae	*Lotus angustissimus*	L.	*Lotus angustissimus*	T23
Fabales	Fabaceae	*Lotus corniculatus*	L.	*Lotus corniculatus*	T23 & this study
Fabales	Fabaceae	*Lotus tetragonolobus*	L.	*Lotus tetragonolobus*	T23
Brassicales	Brassicaceae	*Lunaria annua*	L.	*Lunaria annua*	T23
Fabales	Fabaceae	*Lupinus arboreus*	Sims	*Lupinus arboreus*	T23
Lamiales	Lamiaceae	*Lycopus europaeus*	L.	*Lycopus europaeus*	T23

Order	Family	Name	Author	Name in Thompson et al. (2023)	Source
Ericales	Primulaceae	*Lysimachia arvensis*	(L.) U.Manns & Anderb.	*Anagallis arvensis*	T23
Ericales	Primulaceae	*Lysimachia arvensis*	(L.) U.Manns & Anderb.	*Lysimachia arvensis*	T23
Ericales	Primulaceae	*Lysimachia ciliata*	L.	*Lysimachia ciliata*	T23
Ericales	Primulaceae	*Lysimachia clethroides*	Duby	*Lysimachia clethroides*	T23
Ericales	Primulaceae	*Lysimachia europaea*	(L.) U.Manns & Anderb.	*Lysimachia europaea*	T23
Ericales	Primulaceae	*Lysimachia foemina*	(Mill.) U.Manns & Anderb.	*Lysimachia foemina*	T23
Ericales	Primulaceae	*Lysimachia maritima*	(L.) Galasso, Banfi & Soldano	*Glaux maritima*	T23
Ericales	Primulaceae	*Lysimachia nemorum*	L.	*Lysimachia nemorum*	T23
Ericales	Primulaceae	*Lysimachia nummularia*	L.	*Lysimachia nummularia*	T23
Ericales	Primulaceae	*Lysimachia punctata*	L.	*Lysimachia punctata*	T23
Ericales	Primulaceae	*Lysimachia thyrsiflora*	L.	*Lysimachia thyrsiflora*	T23
Ericales	Primulaceae	*Lysimachia vulgaris*	L.	*Lysimachia vulgaris*	T23
Myrtales	Lythraceae	*Lythrum maritimum*	Kunth	*Lythrum maritimum*	T23
Myrtales	Lythraceae	*Lythrum salicaria*	L.	*Lythrum salicaria*	T23
Asterales	Asteraceae	*Madia elegans*	D.Don	*Madia elegans*	T23
Magnoliales	Magnoliaceae	*Magnolia denudata*	Desr.	*Magnolia denudata*	T23
Magnoliales	Magnoliaceae	*Magnolia liliiflora*	Desr.	*Magnolia liliiflora*	T23
Magnoliales	Magnoliaceae	*Magnolia stellata*	(Siebold & Zucc.) Maxim.	*Magnolia stellata*	T23
Ranunculales	Berberidaceae	*Mahonia aquifolium*	(Pursh) Nutt.	*Mahonia aquifolium*	T23
Asparagales	Asparagaceae	*Maianthemum bifolium*	(L.) F.W.Schmidt	*Maianthemum bifolium*	T23
Rosales	Rosaceae	*Malus domestica*	(Suckow) Borkh.	*Malus domestica*	T23
Rosales	Rosaceae	*Malus sylvestris*	Mill.	*Malus sylvestris*	T23
Malvales	Malvaceae	*Malva moschata*	L.	*Malva moschata*	T23
Malvales	Malvaceae	*Malva neglecta*	Wallr.	*Malva neglecta*	T23
Malvales	Malvaceae	*Malva parviflora*	L.	*Malva parviflora*	T23
Malvales	Malvaceae	*Malva sylvestris*	L.	*Malva sylvestris*	T23 & this study
Cucurbitales	Cucurbitaceae	*Marah fabacea*	(Naudin) Greene	*Echinocystis fabacea*	T23
Rosales	Rosaceae	*Margyricarpus pinnatus*	(Lam.) Kuntze	*Margyricarpus pinnatus*	T23
Asterales	Asteraceae	*Matricaria chamomilla*	L.	*Matricaria camomilla*	T23
Asterales	Asteraceae	*Matricaria discoidea*	DC.	*Matricaria discoidea*	T23
Brassicales	Brassicaceae	*Matthiola incana*	(L.) W.T.Aiton	*Matthiola incana*	T23
Fabales	Fabaceae	*Medicago arabica*	(L.) Huds.	*NA*	This study
Fabales	Fabaceae	*Medicago ciliaris*	(L.) All.	*Medicago cilaris*	T23
Fabales	Fabaceae	*Medicago littoralis*	Rohde ex Loisel.	*Medicago littoralis*	T23
Fabales	Fabaceae	*Medicago lupulina*	L.	*Medicago lupulina*	T23
Fabales	Fabaceae	*Medicago minima*	(L.) Bartal.	*NA*	This study
Fabales	Fabaceae	*Medicago polymorpha*	L.	*Medicago polymorpha*	T23 & this study
Fabales	Fabaceae	*Medicago rigidula*	(L.) All.	*Medicago rigidula*	T23 & this study
Fabales	Fabaceae	*Medicago sativa*	L.	*Medicago sativa*	T23 & this study
Fabales	Fabaceae	*Medicago scutellata*	(L.) Mill.	*Medicago scutellata*	T23
Myrtales	Myrtaceae	*Melaleuca granitica*	(Hawkeswood) Craven & R.D.Edwards	*Calothamnus graniticus*	T23
Lamiales	Orobanchaceae	*Melampyrum nemorosum*	L.	*Melampyrum nemorosum*	T23
Lamiales	Orobanchaceae	*Melampyrum pratense*	L.	*Melampyrum pratense*	T23
Lamiales	Orobanchaceae	*Melampyrum sylvaticum*	L.	*Melampyrum sylvaticum*	T23
Poales	Poaceae	*Melica uniflora*	Retz.	*Melica uniflora*	T23
Fabales	Fabaceae	*Melilotus albus*	Medik.	*Melilotus alba Desv.*	T23
Fabales	Fabaceae	*Melilotus indicus*	(L.) All.	*Melilotus indica*	T23
Fabales	Fabaceae	*Melilotus officinalis*	(L.) Lam.	*Melilotus officinalis*	T23
Lamiales	Lamiaceae	*Melissa officinalis*	L.	*Melissa officinalis*	T23
Lamiales	Lamiaceae	*Mentha aquatica*	L.	*Mentha aquatica*	T23 & this study
Lamiales	Lamiaceae	*Mentha arvensis*	L.	*Mentha arvensis*	T23
Lamiales	Lamiaceae	*Mentha gracilis*	Mentha ×gracilis Sole	*Mentha x gracilis*	T23
Lamiales	Lamiaceae	*Mentha piperita*	Mentha ×piperita L.	*Mentha x piperita*	T23
Lamiales	Lamiaceae	*Mentha spicata*	L.	*Mentha spicata*	T23
Lamiales	Lamiaceae	*Mentha suaveolens*	Ehrh.	*Mentha suaveolens*	T23
Asterales	Menyanthaceae	*Menyanthes trifoliata*	L.	*Menyanthes trifoliata*	T23
Malpighiales	Euphorbiaceae	*Mercurialis perennis*	L.	*Mercurialis perennis*	T23
Myrtales	Myrtaceae	*Metrosideros collina*	(Forst.) A.Gray	*Metrosideros collina*	T23

Order	Family	Name	Author	Name in Thompson et al. (2023)	Source
Poales	Poaceae	*Milium effusum*	L.	*Milium effusum*	T23
Malvales	Malvaceae	*Modiola caroliniana*	(L.) G.Don	*Modiola caroliniana*	T23
Caryophyllales	Caryophyllaceae	*Moehringia trinervia*	(L.) Clairv.	*Moehringia trinervia*	T23
Poales	Poaceae	*Molinia caerulea*	(L.) Moench	*Molinia caerulea*	T23
Lamiales	Lamiaceae	*Monarda fistulosa*	L.	*Monarda fistulosa*	T23
Fagales	Myricaceae	*Morella faya*	(Aiton) Wilbur	*Myrica faya*	T23
Rosales	Moraceae	*Morus alba*	L.	*Morus alba*	T23
Poales	Poaceae	*Muhlenbergia rigens*	(Benth.) Hitchc.	*Muhlenbergia rigens*	T23
Asparagales	Asparagaceae	*Muscari comosum*	(L.) Mill.	*Muscari comosum*	T23
Brassicales	Brassicaceae	*Myagrum perfoliatum*	L.	*Myagrum perfoliatum*	T23
Asterales	Asteraceae	*Mycelis muralis*	(L.) Dumort.	*Lactuca muralis*	T23
Boraginales	Boraginaceae	*Myosotis arvensis*	(L.) Hill	*Myosotis arvensis*	T23
Boraginales	Boraginaceae	*Myosotis azorica*	H.C.Watson	*Myosotis azorica*	T23
Boraginales	Boraginaceae	*Myosotis scorpioides*	L.	*Myosotis scorpioides*	T23
Fagales	Myricaceae	*Myrica gale*	L.	*Myrica gale*	T23
Apiales	Apiaceae	*Myrrhis odorata*	(L.) Scop.	*Myrrhis odorata*	T23
Myrtales	Myrtaceae	*Myrtus communis*	L.	*Myrtus communis*	T23
Ranunculales	Berberidaceae	*Nandina domestica*	Thunb.	*Nandina domestica*	T23
Dioscoreales	Nartheciaceae	*Narthecium ossifragum*	(L.) Huds.	*Narthecium ossifragum*	T23
Poales	Poaceae	*Nassella tenuissima*	(Trin.) Barkworth	*Nassella tenuissima*	T23
Brassicales	Brassicaceae	*Nasturtium officinale*	R.Br.	*Nasturtium officinale*	T23
Asparagales	Orchidaceae	*Neottia ovata*	(L.) Bluff & Fingerh.	*Neottia ovata*	T23
Lamiales	Lamiaceae	*Nepeta cataria*	L.	*Nepeta cataria*	T23
Lamiales	Lamiaceae	*Nepeta faassenii*	Nepeta ×faassenii Bergmans ex Stearn	*Nepeta x faassenii*	T23
Lamiales	Lamiaceae	*Nepeta racemosa*	Lam.	*Nepeta racemosa*	T23
Solanales	Solanaceae	*Nicotiana tabacum*	L.	*Nicotiana tabacum*	T23
Ranunculales	Ranunculaceae	*Nigella damascena*	L.	*Nigella damascena*	T23
Lamiales	Lamiaceae	*Ocimum basilicum*	L.	*Ocimum basilicum*	T23
Asterales	Asteraceae	*Oclemena acuminata*	(Michx.) Greene	*Oclemena acuminata*	T23
Asterales	Asteraceae	*Oclemena nemoralis*	(Aiton) Greene	*Oclemena nemoralis*	T23
Lamiales	Orobanchaceae	*Odontites vernus*	(Bellardi) Dumort.	*Odontites vernus*	T23
Myrtales	Onagraceae	*Oenothera biennis*	L.	*Oenothera biennis*	T23
Myrtales	Onagraceae	*Oenothera glazioviana*	Micheli	*Oenothera glaziovana*	T23
Myrtales	Onagraceae	*Oenothera lindheimeri*	(Engelm. & A.Gray) W.L.Wagner & Hoch	*Oenothera lindheimeri*	T23
Myrtales	Onagraceae	*Oenothera odorata*	Jacq.	*Oenothera odorata*	T23
Myrtales	Onagraceae	*Oenothera stricta*	Link	*Oenothera stricta*	T23
Lamiales	Oleaceae	*Olea europaea*	L.	*Olea europaea*	T23
Fabales	Fabaceae	*Onobrychis viciifolia*	Scop.	*Onobrychis viciifolia*	T23
Fabales	Fabaceae	*Ononis arvensis*	L.	*Ononis arvensis*	T23
Fabales	Fabaceae	*Ononis spinosa*	L.	*Ononis repens*	T23 & this study
Fabales	Fabaceae	*Ononis spinosa*	L.	*Ononis spinosa*	T23 & this study
Asterales	Asteraceae	*Onopordum acanthium*	L.	*Onopordum acanthium*	T23
Lamiales	Lamiaceae	*Origanum majorana*	L.	*Origanum majorana*	T23
Lamiales	Lamiaceae	*Origanum vulgare*	L.	*Origanum vulgare*	T23
Asparagales	Asparagaceae	*Ornithogalum orthophyllum*	Ten.	*Ornithogalum ortophyllum*	T23
Fabales	Fabaceae	*Ornithopus compressus*	L.	*Ornithopus compressus*	T23
Lamiales	Oleaceae	*Osmanthus delavayi*	Franch.	*Osmanthus delavayi*	T23
Osmundales	Osmundaceae	*Osmunda regalis*	L.	*Osmunda regalis*	T23
Fagales	Betulaceae	*Ostrya carpinifolia*	Scop.	*Ostrya carpinifolia*	T23
Oxalidales	Oxalidaceae	*Oxalis acetosella*	L.	*Oxalis acetosella*	T23
Oxalidales	Oxalidaceae	*Oxalis corniculata*	L.	*Oxalis corniculata*	T23
Oxalidales	Oxalidaceae	*Oxalis stricta*	L.	*Oxalis stricta*	T23
Ericales	Ericaceae	*Oxydendrum arboreum*	(L.) DC.	*Oxydendrum arboreum*	T23
Asterales	Asteraceae	*Ozothamnus rosmarinifolius*	(Labill.) DC.	*Ozothamnus rosmarinifolius*	T23
Gentianales	Rubiaceae	*Palicourea elata*	(Sw.) Borhidi	*Palicourea elata*	T23
Asterales	Asteraceae	*Pallenis spinosa*	(L.) Cass.	*Pallenis spinosa*	T23
Poales	Poaceae	*Panicum virgatum*	L.	*Panicum virgatum*	T23
Ranunculales	Papaveraceae	*Papaver cambricum*	L.	*Papaver cambricum*	T23
Ranunculales	Papaveraceae	*Papaver dubium*	L.	*Papaver dubium*	T23
Ranunculales	Papaveraceae	*Papaver nudicaule*	L.	*Papaver nudicaule*	T23
Ranunculales	Papaveraceae	*Papaver orientale*	L.	*Papaver orientale*	T23
Ranunculales	Papaveraceae	*Papaver rhoeas*	L.	*Papaver rhoeas*	T23
Ranunculales	Papaveraceae	*Papaver somniferum*	L.	*Papaver somniferum*	T23

Order	Family	Name	Author	Name in Thompson et al. (2023)	Source
Rosales	Urticaceae	*Parietaria judaica*	L.	*Parietaria judaica*	T23
Rosales	Urticaceae	*Parietaria officinalis*	L.	*Parietaria officinalis*	T23
Vitales	Vitaceae	*Parthenocissus henryana*	(Hemsl.) Diels & Gilg	*Parthenocissus henryana*	T23
Vitales	Vitaceae	*Parthenocissus quinquefolia*	(L.) Planch.	*Parthenocissus quinquefolia*	T23
Apiales	Apiaceae	*Pastinaca sativa*	L.	*Pastinaca sativa*	T23
Lamiales	Orobanchaceae	*Pedicularis palustris*	L.	*Pedicularis palustris*	T23
Geraniales	Geraniaceae	*Pelargonium domesticum*	Pelargonium ×domesticum L.H.Bailey	*Pelargonium × domesticum*	T23
Geraniales	Geraniaceae	*Pelargonium graveolens*	(Thunb.) L'Hér.	*Pelargonium graveolens*	T23
Geraniales	Geraniaceae	*Pelargonium hortorum*	Pelargonium ×hortorum L.H.Bailey	*Pelargonium × hortorum*	T23
Geraniales	Geraniaceae	*Pelargonium peltatum*	(L.) L'Hér.	*Pelargonium peltatum*	T23
Boraginales	Boraginaceae	*Pentaglottis sempervirens*	(L.) Tausch ex L.H.Bailey	*Pentaglottis sempervirens*	T23
Caryophyllales	Polygonaceae	*Persicaria amphibia*	(L.) Gray	*Persicaria amphibia*	T23
Caryophyllales	Polygonaceae	*Persicaria lapathifolia*	(L.) Gray	*Persicaria lapathifolia*	T23
Caryophyllales	Polygonaceae	*Persicaria lapathifolia*	(L.) Gray	*Polygonum lapathifolium*	T23
Caryophyllales	Polygonaceae	*Persicaria maculosa*	Gray	*Persecaria maculosa*	T23
Caryophyllales	Polygonaceae	*Persicaria odorata*	(Lour.) Soják	*Persicaria odorata*	T23
Asterales	Asteraceae	*Petasites hybridus*	(L.) G.Gaertn., B.Mey. & Scherb.	*Petasites hybridus*	T23
Asterales	Asteraceae	*Petasites pyrenaicus*	(L.) G.López	*Petasites pyrenaicus*	T23
Caryophyllales	Caryophyllaceae	*Petrorhagia prolifera*	(L.) P.W.Ball & Heywood	*NA*	This study
Saxifragales	Crassulaceae	*Petrosedum rupestre*	(L.) P.V.Heath	*Petrosedum rupestre*	T23
Apiales	Apiaceae	*Petroselinum crispum*	(Mill.) Fuss	*Petroselinum crispum*	T23
Apiales	Apiaceae	*Peucedanum officinale*	L.	*Peucedanum officinale*	T23
Boraginales	Hydrophyllaceae	*Phacelia tanacetifolia*	Benth.	*Phacelia tanacetifolia*	T23
Poales	Poaceae	*Phalaris aquatica*	L.	*Phalaris aquatica*	T23
Poales	Poaceae	*Phalaris arundinacea*	L.	*Phalaris arundinacea*	T23
Fabales	Fabaceae	*Phaseolus coccineus*	L.	*Phaseolus coccineus*	T23
Fabales	Fabaceae	*Phaseolus vulgaris*	L.	*Phaseolus vulgaris*	T23
Saxifragales	Crassulaceae	*Phedimus spurius*	(M.Bieb.) 't Hart	*Phedimus spurius*	T23
Cornales	Hydrangeaceae	*Philadelphus coronarius*	L.	*Philadelphus coronarius*	T23
Poales	Poaceae	*Phleum pratense*	L.	*Phleum pratense*	T23
Ericales	Polemoniaceae	*Phlox douglasii*	Hook.	*Phlox douglasii*	T23
Ericales	Polemoniaceae	*Phlox paniculata*	L.	*Phlox paniculata*	T23
Ericales	Polemoniaceae	*Phlox subulata*	L.	*Phlox subulata*	T23
Rosales	Rosaceae	*Photinia fraseri*	Photinia ×fraseri Dress	*Photinia x fraseri*	T23
Poales	Poaceae	*Phragmites australis*	(Cav.) Trin. ex Steud.	*Phragmites australis*	T23
Lamiales	Scrophulariaceae	*Phygelius capensis*	E.Mey. ex Benth.	*Phygelius capensis*	T23
Malpighiales	Phyllanthaceae	*Phyllanthus niruri*	L.	*Phyllanthus niruri*	T23
Solanales	Solanaceae	*Physalis peruviana*	L.	*Physalis peruviana*	T23
Rosales	Rosaceae	*Physocarpus opulifolius*	(L.) Maxim.	*Physocarpus opulifolius*	T23
Lamiales	Lamiaceae	*Physostegia virginiana*	(L.) Benth.	*Physostegia virginiana*	T23
Asterales	Campanulaceae	*Phyteuma nigrum*	F.W.Schmidt	*Phyteuma nigrum*	T23
Pinales	Pinaceae	*Picea glauca*	(Moench) Voss	*Picea glauca*	T23
Asterales	Asteraceae	*Picris hieracioides*	L.	*Picris hieracioides*	T23 & this study
Asterales	Asteraceae	*Pilosella aurantiaca*	(L.) F.W.Schultz & Sch.Bip.	*Pilosella aurantiaca*	T23
Asterales	Asteraceae	*Pilosella caespitosa*	(Dumort.) P.D.Sell & C.West	*Pilosella caespitosa*	T23
Asterales	Asteraceae	*Pilosella officinarum*	Vaill.	*Pilosella officinarum*	T23
Apiales	Apiaceae	*Pimpinella anisum*	L.	*Pimpinella anisum*	T23
Apiales	Apiaceae	*Pimpinella major*	(L.) Huds.	*Pimpinella major*	T23
Apiales	Apiaceae	*Pimpinella saxifraga*	L.	*Pimpinella saxifraga*	T23
Pinales	Pinaceae	*Pinus banksiana*	Lamb.	*Pinus banksiana*	T23
Pinales	Pinaceae	*Pinus contorta*	Douglas ex Loudon	*Pinus contorta*	T23
Pinales	Pinaceae	*Pinus halepensis*	Mill.	*Pinus halepensis*	T23
Pinales	Pinaceae	*Pinus ponderosa*	Douglas ex C.Lawson	*Pinus ponderosa*	T23
Pinales	Pinaceae	*Pinus radiata*	D.Don	*Pinus radiata*	T23
Pinales	Pinaceae	*Pinus resinosa*	Aiton	*Pinus resinosa*	T23
Pinales	Pinaceae	*Pinus strobus*	L.	*Pinus strobus*	T23
Pinales	Pinaceae	*Pinus sylvestris*	L.	*Pinus sylvestris*	T23
Pinales	Pinaceae	*Pinus virginiana*	Mill.	*Pinus virginiana*	T23
Sapindales	Anacardiaceae	*Pistacia lentiscus*	L.	*Pistacia lentiscus*	T23

Order	Family	Name	Author	Name in Thompson et al. (2023)	Source
Sapindales	Anacardiaceae	*Pistacia terebinthus*	L.	*Pistacia terebinthus*	T23
Apiales	Pittosporaceae	*Pittosporum tenuifolium*	Gaertn.	*Pittosporum tenuifolium*	T23
Apiales	Pittosporaceae	*Pittosporum undulatum*	Vent.	*Pittosporum undulatum*	T23
Lamiales	Plantaginaceae	*Plantago coronopus*	L.	*Plantago coronopus*	T23 & this study
Lamiales	Plantaginaceae	*Plantago lagopus*	L.	*Plantago lagopus*	T23
Lamiales	Plantaginaceae	*Plantago lanceolata*	L.	*Plantago lanceolata*	T23 & this study
Lamiales	Plantaginaceae	*Plantago major*	L.	*Plantago major*	T23
Lamiales	Plantaginaceae	*Plantago maritima*	L.	*Plantago maritima*	T23
Lamiales	Plantaginaceae	*Plantago media*	L.	*Plantago media*	T23
Lamiales	Plantaginaceae	*Plantago rugelii*	Decne.	*Plantago rugelii*	T23
Lamiales	Plantaginaceae	*Plantago sempervirens*	Crantz	*NA*	This study
Proteales	Platanaceae	*Platanus occidentalis*	L.	*Platanus occidentalis*	T23
Myrtales	Melastomataceae	*Pleroma semidecandrum*	(Schrank & Mart. ex DC.) Triana	*Tibouchina semidecandra*	T23
Asterales	Asteraceae	*Pluchea odorata*	(L.) Cass.	*Pluchea odorata*	T23
Poales	Poaceae	*Poa annua*	L.	*Poa annua*	T23
Poales	Poaceae	*Poa pratensis*	L.	*Poa pratensis*	T23
Poales	Poaceae	*Poa trivialis*	L.	*Poa trivialis*	T23 & this study
Ranunculales	Berberidaceae	*Podophyllum peltatum*	L.	*Podophyllum peltatum*	T23
Ericales	Polemoniaceae	*Polemonium caeruleum*	L.	*Polemonium caeruleum*	T23
Caryophyllales	Polygonaceae	*Polygonum aviculare*	L.	*Polygonum aviculare*	T23
Malpighiales	Salicaceae	*Populus nigra*	L.	*Populus nigra*	T23
Malpighiales	Salicaceae	*Populus tremula*	L.	*Populus tremula*	T23
Rosales	Rosaceae	*Potentilla argentea*	L.	*Potentilla argentea*	T23
Rosales	Rosaceae	*Potentilla canadensis*	L.	*Potentilla canadensis*	T23
Rosales	Rosaceae	*Potentilla erecta*	(L.) Raeusch.	*Potentilla erecta*	T23
Rosales	Rosaceae	*Potentilla norvegica*	L.	*Potentilla norvegica*	T23
Rosales	Rosaceae	*Potentilla recta*	L.	*Potentilla recta*	T23
Rosales	Rosaceae	*Potentilla reptans*	L.	*Potentilla reptans*	T23 & this study
Rosales	Rosaceae	*Potentilla sterilis*	(L.) Garcke	*Potentilla sterilis*	T23
Rosales	Rosaceae	*Poterium sanguisorba*	L.	*Sanguisorba minor*	T23 & this study
Rosales	Rosaceae	*Poterium verrucosum*	Link ex G.Don	*Sanguisorba verrucosa*	T23
Ericales	Primulaceae	*Primula auricula*	L.	*Primula auricula*	T23
Ericales	Primulaceae	*Primula denticulata*	Sm.	*Primula denticulata*	T23
Ericales	Primulaceae	*Primula prolifera*	Wall.	*Primula prolifera*	T23
Ericales	Primulaceae	*Primula rosea*	Royle	*Primula rosea*	T23
Ericales	Primulaceae	*Primula veris*	L.	*Primula veris*	T23
Ericales	Primulaceae	*Primula vialii*	Delavay ex Franch.	*Primula vialii*	T23
Ericales	Primulaceae	*Primula vulgaris*	Huds.	*Primula vulgaris*	T23
Lamiales	Lamiaceae	*Prunella vulgaris*	L.	*Prunella vulgaris*	T23
Rosales	Rosaceae	*Prunus americana*	Marshall	*Prunus americana*	T23
Rosales	Rosaceae	*Prunus amygdalus*	Batsch	*Prunus dulcis*	T23
Rosales	Rosaceae	*Prunus armeniaca*	L.	*Prunus armeniaca*	T23
Rosales	Rosaceae	*Prunus avium*	(L.) L.	*Prunus avium*	T23
Rosales	Rosaceae	*Prunus cerasifera*	Ehrh.	*Prunus cerasifera*	T23
Rosales	Rosaceae	*Prunus cerasus*	L.	*Prunus cerasus*	T23
Rosales	Rosaceae	*Prunus domestica*	L.	*Prunus domestica*	T23
Rosales	Rosaceae	*Prunus laurocerasus*	L.	*Prunus laurocerasus*	T23
Rosales	Rosaceae	*Prunus padus*	L.	*Prunus padus*	T23
Rosales	Rosaceae	*Prunus pensylvanica*	L.fil.	*Prunus pensylvanica*	T23
Rosales	Rosaceae	*Prunus persica*	(L.) Stokes	*Prunus persica*	T23
Rosales	Rosaceae	*Prunus serrulata*	Lindl.	*Prunus serrulata*	T23
Rosales	Rosaceae	*Prunus spinosa*	L.	*Prunus spinosa*	T23
Rosales	Rosaceae	*Prunus virginiana*	L.	*Prunus virginiana*	T23
Asterales	Asteraceae	*Psephellus dealbatus*	(Willd.) K.Koch	*Psephellus dealbatus*	T23
Polypodiales	Dennstaedtiaceae	*Pteridium aquilinum*	(L.) Kuhn	*Pteridium aquilinum*	T23
Asterales	Asteraceae	*Pulicaria dysenterica*	(L.) Bernh.	*Pulicaria dysenterica*	T23
Rosales	Rosaceae	*Pyracantha crenulata*	(D.Don) M.Roem.	*Pyracantha rogersiana*	T23
Ericales	Ericaceae	*Pyrola minor*	L.	*Pyrola minor*	T23
Rosales	Rosaceae	*Pyrus communis*	L.	*Pyrus communis*	T23
Rosales	Rosaceae	*Pyrus spinosa*	Forssk.	*Pyrus spinosa*	T23
Fagales	Fagaceae	*Quercus agrifolia*	Née	*Quercus agrifolia*	T23
Fagales	Fagaceae	*Quercus cerris*	L.	*Quercus cerris*	T23

Order	Family	Name	Author	Name in Thompson et al. (2023)	Source
Fagales	Fagaceae	*Quercus crenata*	Quercus ×crenata Lam.	*Quercus crenata*	T23
Fagales	Fagaceae	*Quercus ilex*	L.	*Quercus ilex*	T23
Fagales	Fagaceae	*Quercus infectoria*	G.Olivier	*Quercus infectoria*	T23
Fagales	Fagaceae	*Quercus petraea*	(Matt.) Liebl.	*Quercus petraea.*	T23
Fagales	Fagaceae	*Quercus pubescens*	Willd.	*Quercus pubescens*	T23
Fagales	Fagaceae	*Quercus robur*	L.	*Quercus robur*	T23 & this study
Fagales	Fagaceae	*Quercus trojana*	Webb	*Quercus trojana*	T23
Caryophyllales	Caryophyllaceae	*Rabelera holostea*	(L.) M.T.Sharples & E.A.Tripp	*Rabelera holostea*	T23
Ranunculales	Ranunculaceae	*Ranunculus abortivus*	L.	*Ranunculus abortivus*	T23
Ranunculales	Ranunculaceae	*Ranunculus acris*	L.	*Ranunculus acris*	T23 & this study
Ranunculales	Ranunculaceae	*Ranunculus arvensis*	L.	*Ranunculus arvensis*	T23
Ranunculales	Ranunculaceae	*Ranunculus auricomus*	L.	*Ranunculus auricomus*	T23
Ranunculales	Ranunculaceae	*Ranunculus bulbosus*	L.	*Ranunculus bulbosus*	T23 & this study
Ranunculales	Ranunculaceae	*Ranunculus flammula*	L.	*Ranunculus flammula*	T23
Ranunculales	Ranunculaceae	*Ranunculus lingua*	L.	*Ranunculus lingua*	T23
Ranunculales	Ranunculaceae	*Ranunculus repens*	L.	*Ranunculus repens*	T23 & this study
Ranunculales	Ranunculaceae	*Ranunculus sceleratus*	L.	*Ranunculus sceleratus*	T23
Brassicales	Brassicaceae	*Raphanus raphanistrum*	L.	*Raphanus raphanistrum*	T23 & this study
Brassicales	Brassicaceae	*Rapistrum rugosum*	(L.) All.	*NA*	This study
Brassicales	Resedaceae	*Reseda lutea*	L.	*Reseda lutea*	T23
Caryophyllales	Polygonaceae	*Reynoutria multiflora*	(Thunb.) Moldenke	*Reynoutria multiflora*	T23
Rosales	Rhamnaceae	*Rhamnus alaternus*	L.	*Rhamnus alaternus*	T23
Rosales	Rhamnaceae	*Rhamnus cathartica*	L.	*Rhamnus cathartica*	T23
Asterales	Asteraceae	*Rhaponticoides centaurium*	(L.) M.V.Agab. & Greuter	*Centaurea centaurium*	T23
Caryophyllales	Polygonaceae	*Rheum hybridum*	Rheum ×hybridum Murray	*Rheum x hybridum (?)*	T23
Caryophyllales	Polygonaceae	*Rheum rhaponticum*	L.	*Rheum rhaponticum*	T23
Lamiales	Orobanchaceae	*Rhinanthus minor*	L.	*Rhinanthus minor*	T23
Lamiales	Orobanchaceae	*Rhinanthus serotinus*	(Schönh. ex Halácsy & Heinr.Braun) Oborny	*Rhinanthus serotinus*	T23
Sapindales	Anacardiaceae	*Rhus glabra*	L.	*Rhus glabra*	T23
Saxifragales	Grossulariaceae	*Ribes nigrum*	L.	*Ribes nigrum*	T23
Saxifragales	Grossulariaceae	*Ribes rubrum*	L.	*Ribes rubrum*	T23
Saxifragales	Grossulariaceae	*Ribes sanguineum*	Pursh	*Ribes sanguineum*	T23
Saxifragales	Grossulariaceae	*Ribes uva-crispa*	L.	*Ribes uva-crispa*	T23
Malpighiales	Euphorbiaceae	*Ricinus communis*	L.	*Ricinus communis*	T23
Fabales	Fabaceae	*Robinia pseudoacacia*	L.	*Robinia pseudoacacia*	T23
Ranunculales	Papaveraceae	*Roemeria argemone*	(L.) C.Morales, R.Mend. & Romero García	*Roemeria argemone*	T23
Brassicales	Brassicaceae	*Rorippa amphibia*	(L.) Besser	*Rorippa amphibia*	T23
Rosales	Rosaceae	*Rosa arvensis*	Huds.	*Rosa arvensis*	T23
Rosales	Rosaceae	*Rosa canina*	L.	*Rosa canina*	T23
Rosales	Rosaceae	*Rosa chinensis*	Jacq.	*Rosa chinensis*	T23
Rosales	Rosaceae	*Rosa damascena*	Rosa ×damascena Mill.	*Rosa × damascena*	T23
Rosales	Rosaceae	*Rosa foetida*	Herrm.	*Rosa foetida*	T23
Rosales	Rosaceae	*Rosa gallica*	L.	*Rosa gallica*	T23
Rosales	Rosaceae	*Rosa multiflora*	Thunb.	*Rosa multiflora*	T23
Rosales	Rosaceae	*Rosa nitida*	Willd.	*Rosa nitida*	T23
Rosales	Rosaceae	*Rosa rubiginosa*	L.	*Rosa rubiginosa*	T23
Rosales	Rosaceae	*Rosa rugosa*	Thunb.	*Rosa rugosa*	T23
Rosales	Rosaceae	*Rosa setigera*	Michx.	*Rosa setigera*	T23
Rosales	Rosaceae	*Rosa spinosissima*	L.	*Rosa pimpinellifolia*	T23
Gentianales	Rubiaceae	*Rubia peregrina*	L.	*Rubia peregrina*	T23 & this study
Gentianales	Rubiaceae	*Rubia tinctorum*	L.	*Rubia tinctorum*	T23
Rosales	Rosaceae	*Rubus allegheniensis*	Porter	*Rubus allegheniensis*	T23
Rosales	Rosaceae	*Rubus arcticus*	L.	*Rubus arcticus*	T23
Rosales	Rosaceae	*Rubus argutus*	Link	*Rubus argutus*	T23
Rosales	Rosaceae	*Rubus armeniacus*	Focke	*Rubus armeniacus*	T23
Rosales	Rosaceae	*Rubus caesius*	L.	*Rubus caesius*	T23
Rosales	Rosaceae	*Rubus canadensis*	L.	*Rubus canadensis*	T23
Rosales	Rosaceae	*Rubus canescens*	A.DC.	*Rubus canescens*	T23
Rosales	Rosaceae	*Rubus chamaemorus*	L.	*Rubus chamaemorus*	T23
Rosales	Rosaceae	*Rubus fruticosus*	L.	*Rubus fruticosus*	T23
Rosales	Rosaceae	*Rubus hispidus*	L.	*Rubus hispidus*	T23
Rosales	Rosaceae	*Rubus idaeus*	L.	*Rubus idaeus*	T23

Order	Family	Name	Author	Name in Thompson et al. (2023)	Source
Rosales	Rosaceae	*Rubus laciniatus*	Willd.	*Rubus laciniatus*	T23
Rosales	Rosaceae	*Rubus loganobaccus*	Rubus ×loganobaccus L.H.Bailey	*Rubus × loganobaccus*	T23
Rosales	Rosaceae	*Rubus occidentalis*	L.	*Rubus occidentalis*	T23
Rosales	Rosaceae	*Rubus parviflorus*	Kük.	*Rubus parviflorus*	T23
Rosales	Rosaceae	*Rubus procerus*	P.J.Müll. ex Genev.	*Rubus procerus*	T23
Rosales	Rosaceae	*Rubus rosaceus*	Weihe	*Rubus rosaceus*	T23
Rosales	Rosaceae	*Rubus saxatilis*	L.	*Rubus saxatilis*	T23
Rosales	Rosaceae	*Rubus ulmifolius*	Schott	*Rubus ulmifolius*	T23
Rosales	Rosaceae	*Rubus ursinus*	Cham. & Schltdl.	*Rubus ursinus*	T23
Rosales	Rosaceae	*Rubus vitifolius*	Cham. & Schltdl.	*Rubus vitifolius*	T23
Asterales	Asteraceae	*Rudbeckia fulgida*	Aiton	*Rudbeckia fulgida*	T23
Asterales	Asteraceae	*Rudbeckia hirta*	L.	*Rudbeckia hirta*	T23
Asterales	Asteraceae	*Rudbeckia laciniata*	L.	*Rudbeckia laciniata*	T23
Caryophyllales	Polygonaceae	*Rumex acetosa*	L.	*Rumex acetosa*	T23 & this study
Caryophyllales	Polygonaceae	*Rumex acetosella*	L.	*Rumex acetosella*	T23 & this study
Caryophyllales	Polygonaceae	*Rumex aquaticus*	L.	*Rumex aquaticus*	T23
Caryophyllales	Polygonaceae	*Rumex bucephalophorus*	L.	*Rumex bucephalophorus*	T23
Caryophyllales	Polygonaceae	*Rumex conglomeratus*	Murray	*Rumex conglomeratus*	T23 & this study
Caryophyllales	Polygonaceae	*Rumex crispus*	L.	*Rumex crispis*	T23 & this study
Caryophyllales	Polygonaceae	*Rumex hydrolapathum*	(Scop.) Huds.	*Rumex hydrolapathum*	T23
Caryophyllales	Polygonaceae	*Rumex induratus*	Boiss. & Reut.	*Rumex induratus*	T23
Caryophyllales	Polygonaceae	*Rumex longifolius*	DC.	*Rumex longifolius*	T23
Caryophyllales	Polygonaceae	*Rumex obtusifolius*	L.	*Rumex obtusifolius*	T23
Caryophyllales	Polygonaceae	*Rumex occidentalis*	(Michx.) S.Watson	*Rumex occidentalis*	T23
Caryophyllales	Polygonaceae	*Rumex sanguineus*	L.	*Rumex sanguineus*	T23
Asparagales	Asparagaceae	*Ruscus aculeatus*	L.	*Ruscus aculeatus*	T23
Poales	Poaceae	*Sacciolepis indica*	(L.) Chase	*Sacciolepis indica*	T23
Caryophyllales	Caryophyllaceae	*Sagina apetala*	Ard.	*Sagina apetala*	T23
Aplousobranchia	Polycitoridae	*Salix alba*	(Crawford, 1914) Crawford, 1914	*Salix alba*	T23
Malpighiales	Salicaceae	*Salix babylonica*	L.	*Salix babylonica*	T23
Malpighiales	Salicaceae	*Salix caprea*	L.	*Salix caprea*	T23
Malpighiales	Salicaceae	*Salix cinerea*	L.	*Salix cinerea*	T23 & this study
Malpighiales	Salicaceae	*Salix fragilis*	L.	*Salix fragilis*	T23
Malpighiales	Salicaceae	*Salix phylicifolia*	L.	*Salix phylicifolia*	T23
Malpighiales	Salicaceae	*Salix purpurea*	L.	*Salix purpurea*	T23
Malpighiales	Salicaceae	*Salix repens*	L.	*Salix repens*	T23
Malpighiales	Salicaceae	*Salix viminalis*	L.	*Salix viminalis*	T23
Lamiales	Lamiaceae	*Salvia coccinea*	Buc'hoz ex Etl.	*Salvia coccinea*	T23
Lamiales	Lamiaceae	*Salvia elegans*	Vahl	*Salvia elegans*	T23
Lamiales	Lamiaceae	*Salvia farinacea*	Benth.	*Salvia farinacea*	T23
Lamiales	Lamiaceae	*Salvia guaranitica*	A.St.-Hil. ex Benth.	*Salvia guaranitica*	T23
Lamiales	Lamiaceae	*Salvia jamensis*	Salvia ×jamensis J.Compton	*Salvia x jamensis*	T23
Lamiales	Lamiaceae	*Salvia longispicata*	M.Martens & Galeotti	*Salvia longispicata*	T23
Lamiales	Lamiaceae	*Salvia microphylla*	Kunth	*Salvia mircophylla*	T23
Lamiales	Lamiaceae	*Salvia nemorosa*	L.	*Salvia nemorosa*	T23
Lamiales	Lamiaceae	*Salvia officinalis*	L.	*Salvia officialis*	T23
Lamiales	Lamiaceae	*Salvia pratensis*	L.	*NA*	This study
Lamiales	Lamiaceae	*Salvia rosmarinus*	Spenn.	*Salvia rosmarinus*	T23
Lamiales	Lamiaceae	*Salvia splendens*	Sellow ex Nees	*Salvia splendens*	T23
Lamiales	Lamiaceae	*Salvia verbenaca*	L.	*NA*	This study
Lamiales	Lamiaceae	*Salvia yangii*	B.T.Drew	*Salvia yangii*	T23
Dipsacales	Viburnaceae	*Sambucus canadensis*	L.	*Sambucus canadensis*	T23
Dipsacales	Viburnaceae	*Sambucus nigra*	L.	*Sambucus nigra*	T23
Dipsacales	Viburnaceae	*Sambucus racemosa*	L.	*Sambucus racemosa*	T23
Rosales	Rosaceae	*Sanguisorba officinalis*	L.	*Sanguisorba officinalis*	T23
Apiales	Apiaceae	*Sanicula liberta*	Cham. & Schltdl.	*Sanicula liberta*	T23
Asterales	Asteraceae	*Santolina chamaecyparissus*	L.	*Santolina chamaecyparissus*	T23
Caryophyllales	Caryophyllaceae	*Saponaria officinalis*	L.	*Saponaria officinalis*	T23
Buxales	Buxaceae	*Sarcococca confusa*	Sealy	*Sarcococca confusa*	T23
Lamiales	Lamiaceae	*Satureja hortensis*	L.	*Satureja hortensis*	T23
Lamiales	Lamiaceae	*Satureja montana*	L.	*Satureja montana*	T23
Dipsacales	Caprifoliaceae	*Scabiosa columbaria*	L.	*Scabiosa columbaria*	T23

Order	Family	Name	Author	Name in Thompson et al. (2023)	Source
Apiales	Apiaceae	*Scandix pecten-veneris*	L.	*Scandix pecten-veneris*	T23
Poales	Cyperaceae	*Schoenoplectus tabernaemontani*	(C.C.Gmel.) Palla	*Schoenoplectus tabernaemontani*	T23
Caryophyllales	Caryophyllaceae	*Scleranthus annuus*	L.	*Scleranthus annuus*	T23
Asterales	Asteraceae	*Scolymus hispanicus*	L.	*Scolymus hispanicus*	T23
Asterales	Asteraceae	*Scorzonera aristata*	Ramond ex DC.	*Scorzonera aristata*	T23
Asterales	Asteraceae	*Scorzonera laciniata*	L.	*NA*	This study
Asterales	Asteraceae	*Scorzoneroides autumnalis*	(L.) Moench	*Scorzoneroides autumnalis*	T23
Lamiales	Scrophulariaceae	*Scrophularia auriculata*	L.	*Scrophularia auriculata*	T23
Lamiales	Scrophulariaceae	*Scrophularia californica*	Cham. & Schltdl.	*Scrophularia californica*	T23
Lamiales	Scrophulariaceae	*Scrophularia nodosa*	L.	*Scrophularia nodosa*	T23
Lamiales	Lamiaceae	*Scutellaria galericulata*	L.	*Scutellaria galericulata*	T23
Poales	Poaceae	*Secale cereale*	L.	*Secale cereale*	T23
Saxifragales	Crassulaceae	*Sedum acre*	L.	*Sedum acre*	T23
Saxifragales	Crassulaceae	*Sedum album*	L.	*Sedum album*	T23
Asterales	Asteraceae	*Senecio jacobaea*	Loscos & Pardo	*Senecio jacobaea*	T23
Asterales	Asteraceae	*Senecio mikanioides*	Otto	*Senecio mikanioides*	T23
Asterales	Asteraceae	*Senecio squalidus*	L.	*Senecio squalidus*	T23
Asterales	Asteraceae	*Senecio vulgaris*	L.	*Senecio vulgaris*	T23
Fabales	Fabaceae	*Senna corymbosa*	(Lam.) H.S.Irwin & Barneby	*Senna corymbosa*	T23
Apiales	Apiaceae	*Seseli tortuosum*	L.	*Seseli tortuosum*	T23
Caryophyllales	Aizoaceae	*Sesuvium portulacastrum*	(L.) L.	*Sesuvium portulacastrum*	T23
Gentianales	Rubiaceae	*Sherardia arvensis*	L.	*Sherardia arvensis*	T23 & this study
Caryophyllales	Caryophyllaceae	*Silene coronaria*	(L.) Clairv.	*Silene coronaria*	T23
Caryophyllales	Caryophyllaceae	*Silene dioica*	(L.) Clairv.	*Silene dioica*	T23
Caryophyllales	Caryophyllaceae	*Silene flos-cuculi*	(L.) Greuter & Burdet	*Silene flos-cuculi*	T23 & this study
Caryophyllales	Caryophyllaceae	*Silene gallica*	L.	*Silene gallica*	T23 & this study
Caryophyllales	Caryophyllaceae	*Silene latifolia*	Poir.	*Silene latifolia*	T23 & this study
Caryophyllales	Caryophyllaceae	*Silene noctiflora*	L.	*Silene noctiflora*	T23
Caryophyllales	Caryophyllaceae	*Silene nocturna*	L.	*NA*	This study
Caryophyllales	Caryophyllaceae	*Silene uniflora*	Roth	*Silene uniflora*	T23
Caryophyllales	Caryophyllaceae	*Silene vulgaris*	(Moench) Garcke	*Silene vulgaris*	T23 & this study
Asterales	Asteraceae	*Silybum marianum*	(L.) Gaertn.	*Silybum marianum*	T23
Asparagales	Asphodelaceae	*Simethis planifolia*	J.Lloyd & Foucaud, 1886	*Simethis planifolia*	T23
Brassicales	Brassicaceae	*Sinapis arvensis*	L.	*Sinapis arvensis*	T23 & this study
Brassicales	Brassicaceae	*Sisymbrium altissimum*	L.	*Sisymbrium altissimum*	T23
Brassicales	Brassicaceae	*Sisymbrium officinale*	(L.) Scop.	*Sisymbrium officinale*	T23
Dipsacales	Caprifoliaceae	*Sixalix atropurpurea*	(L.) Greuter & Burdet	*Scabiosa atropurpurea*	T23
Dipsacales	Caprifoliaceae	*Sixalix maritima*	(L.) Rottenst.	*NA*	This study
Apiales	Apiaceae	*Smyrnium olusatrum*	L.	*Smyrnium olusatrum*	T23
Solanales	Solanaceae	*Solanum dulcamara*	L.	*Solanum dulcamara*	T23
Solanales	Solanaceae	*Solanum lycopersicoides*	Dunal	*Lycoperscion esculentum*	T23
Solanales	Solanaceae	*Solanum lycopersicum*	L.	*Solanum lycopersicum*	T23
Solanales	Solanaceae	*Solanum nigrum*	L.	*Solanum nigrum*	T23
Solanales	Solanaceae	*Solanum tuberosum*	L.	*Solanum tuberosum*	T23
Asterales	Asteraceae	*Solidago altissima*	L.	*Solidago altissima*	T23
Asterales	Asteraceae	*Solidago canadensis*	L.	*Solidago canadensis*	T23
Asterales	Asteraceae	*Solidago gigantea*	Aiton	*Solidago gigantea*	T23
Asterales	Asteraceae	*Solidago rugosa*	Mill.	*Solidago rugosa*	T23
Asterales	Asteraceae	*Solidago sempervirens*	L.	*Solidago sempervirens*	T23
Asterales	Asteraceae	*Solidago shortii*	Torr. & A.Gray	*Solidago shortii*	T23
Asterales	Asteraceae	*Solidago virgaurea*	L.	*Solidago virgaurea*	T23
Asterales	Asteraceae	*Sonchus arvensis*	L.	*Sonchus arvensis*	T23
Asterales	Asteraceae	*Sonchus asper*	(L.) Hill	*Sonchus asper*	T23 & this study

Order	Family	Name	Author	Name in Thompson et al. (2023)	Source
Asterales	Asteraceae	*Sonchus oleraceus*	L.	*Sonchus oleraceus*	T23 & this study
Asterales	Asteraceae	*Sonchus tenerrimus*	L.	*Sonchus terrenimus*	T23
Rosales	Rosaceae	*Sorbaria sorbifolia*	(L.) A.Braun	*Sorbaria sorbifolia*	T23
Rosales	Rosaceae	*Sorbus americana*	Marshall	*Sorbus americana*	T23
Rosales	Rosaceae	*Sorbus aucuparia*	L.	*Sorbus aucuparia*	T23
Poales	Poaceae	*Sorghum halepense*	(L.) Pers.	*Sorghum halepense*	T23
Caryophyllales	Amaranthaceae	*Spinacia oleracea*	L.	*Spinacia oleracea*	T23
Rosales	Rosaceae	*Spiraea alba*	Du Roi	*Spiraea alba*	T23
Rosales	Rosaceae	*Spiraea arguta*	Spiraea ×arguta Zabel	*Spiraea arguta*	T23
Rosales	Rosaceae	*Spiraea cantoniensis*	Lour.	*Spiraea cantoniensis*	T23
Rosales	Rosaceae	*Spiraea douglasii*	Hook.	*Spiraea douglasii*	T23
Rosales	Rosaceae	*Spiraea japonica*	L.fil.	*Spiraea japonica*	T23
Rosales	Rosaceae	*Spiraea nipponica*	Maxim.	*Spiraea nipponica*	T23
Rosales	Rosaceae	*Spiraea splendens*	Baumann ex K.Koch	*Spiraea splendens*	T23
Rosales	Rosaceae	*Spiraea vanhouttei*	Spiraea ×vanhouttei (Briot) Zabel	*Spiraea vanhouttei*	T23
Lamiales	Lamiaceae	*Stachys ajugoides*	Benth.	*Stachys ajugoides*	T23
Lamiales	Lamiaceae	*Stachys bullata*	Benth.	*Stachys bullata*	T23
Lamiales	Lamiaceae	*Stachys byzantina*	K.Koch	*Stachys byzantina*	T23
Lamiales	Lamiaceae	*Stachys germanica*	L.	*Stachys germanica*	T23
Lamiales	Lamiaceae	*Stachys palustris*	L.	*Stachys palustris*	T23
Lamiales	Lamiaceae	*Stachys rigida*	Nutt. ex Benth.	*Stachys rigida*	T23
Lamiales	Lamiaceae	*Stachys sylvatica*	L.	*Stachys sylvatica*	T23
Caryophyllales	Caryophyllaceae	*Stellaria alsine*	Grimm	*Stellaria alsine*	T23
Caryophyllales	Caryophyllaceae	*Stellaria graminea*	L.	*Stellaria graminea*	T23
Caryophyllales	Caryophyllaceae	*Stellaria media*	(L.) Vill.	*Stellaria media*	T23
Caryophyllales	Caryophyllaceae	*Stellaria nemorum*	L.	*Stellaria nemorum*	T23
Caryophyllales	Caryophyllaceae	*Stellaria palustris*	(Murray ex Ehrh.) Hoffm.	*Stellaria palustris*	T23
Asterales	Asteraceae	*Stokesia laevis*	(Hill) Greene	*Stokesia laevis*	T23
Ranunculales	Papaveraceae	*Stylophorum diphyllum*	(Michx.) Nutt.	*Stylophorum diphyllum*	T23
Dipsacales	Caprifoliaceae	*Succisa pratensis*	Moench	*Succisa pratensis*	T23 & this study
Dipsacales	Caprifoliaceae	*Succisella inflexa*	(Kluk) G.Beck	*Succisella inflexa*	T23
Dipsacales	Caprifoliaceae	*Symphoricarpos albus*	(L.) K.Koch	*Symphoricarpos albus*	T23
Asterales	Asteraceae	*Symphyotrichum cordifolium*	(L.) G.L.Nesom	*Symphyotrichum cordifolium*	T23
Asterales	Asteraceae	*Symphyotrichum ericoides*	(L.) G.L.Nesom	*Symphyotrichum ericoides*	T23
Asterales	Asteraceae	*Symphyotrichum lanceolatum*	(Willd.) G.L.Nesom	*Symphyotrichum lanceolatus*	T23
Asterales	Asteraceae	*Symphyotrichum lateriflorum*	(L.) Á.Löve & D.Löve	*Symphyotrichum lateriflorum*	T23
Asterales	Asteraceae	*Symphyotrichum novae-angliae*	(L.) G.L.Nesom	*Symphyotrichum novae-angliae*	T23
Asterales	Asteraceae	*Symphyotrichum novi-belgii*	(L.) G.L.Nesom	*Symphyotrichum novi-belgii*	T23
Asterales	Asteraceae	*Symphyotrichum tradescantii*	(L.) G.L.Nesom	*Symphyotrichum tradescantii*	T23
Asterales	Asteraceae	*Symphyotrichum turbinellum*	(Lindl.) G.L.Nesom	*Symphyotrichum turbinellum*	T23
Boraginales	Boraginaceae	*Symphytum officinale*	L.	*Symphytum officinale*	T23
Boraginales	Boraginaceae	*Symphytum uplandicum*	Symphytum ×uplandicum Nyman	*Symphytum x uplandicum*	T23
Lamiales	Oleaceae	*Syringa vulgaris*	L.	*Syringa vulgaris*	T23
Asterales	Asteraceae	*Tagetes lucida*	Cav.	*Tagetes lucida*	T23
Malvales	Malvaceae	*Talipariti tiliaceum*	(L.) Fryxell	*Hibiscus tiliaceus*	T23
Caryophyllales	Tamaricaceae	*Tamarix tetrandra*	Pall. ex M.Bieb.	*Tamarix tetrandra*	T23
Asterales	Asteraceae	*Tanacetum parthenium*	(L.) Sch.Bip.	*Tanacetum parthenium*	T23
Asterales	Asteraceae	*Tanacetum vulgare*	L.	*Chrysanthemum vulgare*	T23
Asterales	Asteraceae	*Tanacetum vulgare*	L.	*Tanacetum vulgare*	T23
Asterales	Asteraceae	*Taraxacum kok-saghyz*	Rodin	*Taraxacum kok-saghyz*	T23
Asterales	Asteraceae	*Taraxacum officinale*	Weber ex F.H.Wigg.	*Taraxacum officinale*	T23
Pinales	Taxaceae	*Taxus baccata*	L.	*Taxus baccata*	T23
Saxifragales	Saxifragaceae	*Tellima grandiflora*	(Pursh) Douglas ex Lindl.	*Tellima grandiflora*	T23
Lamiales	Lamiaceae	*Teucrium scorodonia*	L.	*Teucrium scorodonia*	T23
Ranunculales	Ranunculaceae	*Thalictrum flavum*	L.	*Thalictrum flavum*	T23
Polypodiales	Thelypteridaceae	*Thelypteris palustris*	Schott	*Thelypteris palustris*	T23
Brassicales	Brassicaceae	*Thlaspi arvense*	L.	*Thlaspi arvense*	T23
Asterales	Asteraceae	*Thrincia saxatilis*	(Lam.) Holub & Moravec	*NA*	This

Order	Family	Name	Author	Name in Thompson et al. (2023)	Source
					study
Lamiales	Lamiaceae	*Thymus citriodorus*	Thymus ×citriodorus (Pers.) Schreb.	*Thymus citriodorus*	T23
Lamiales	Lamiaceae	*Thymus mastichina*	(L.) L.	*Thymus mastichina*	T23
Lamiales	Lamiaceae	*Thymus serpyllum*	L.	*Thymus serpyllum*	T23
Lamiales	Lamiaceae	*Thymus vulgaris*	L.	*Thymus vulgaris*	T23
Apiales	Apiaceae	*Thysselinum palustre*	(L.) Hoffm.	*Peucedanum palustre*	T23
Malvales	Malvaceae	*Tilia platyphyllos*	Scop.	*Tilia platyphyllos*	T23
Asterales	Asteraceae	*Tolpis umbellata*	Bertol.	*Tolpis umbellata*	T23
Apiales	Apiaceae	*Torilis arvensis*	(Huds.) Link	*NA*	This study
Apiales	Apiaceae	*Torilis japonica*	(Houtt.) DC.	*Torilis japonica*	T23
Apiales	Apiaceae	*Torilis nodosa*	(L.) Gaertn.	*Torilis nodosa*	T23
Sapindales	Anacardiaceae	*Toxicodendron diversilobum*	(Torr. & A.Gray) Greene	*Toxicodendron diversilobum*	T23
Sapindales	Anacardiaceae	*Toxicodendron radicans*	(L.) Kuntze	*Toxicodendron radicans*	T23
Gentianales	Apocynaceae	*Trachelospermum jasminoides*	(Lindl.) Lem.	*Trachelospermum jasminoides*	T23
Commelinales	Commelinaceae	*Tradescantia fluminensis*	Vell.	*Tradescantia fluminensi*	T23
Asterales	Asteraceae	*Tragopogon dubius*	Scop.	*Tragopogon dubius*	T23 & this study
Asterales	Asteraceae	*Tragopogon porrifolius*	L.	*Tragopogon porrifolius*	T23 & this study
Asterales	Asteraceae	*Tragopogon pratensis*	L.	*Tragopogon pratensis*	T23
Fabales	Fabaceae	*Trifolium alexandrinum*	L.	*Trifolium alexandrinum*	T23
Fabales	Fabaceae	*Trifolium arvense*	L.	*Trifolium arvense*	T23
Fabales	Fabaceae	*Trifolium campestre*	Schreb.	*Trifolium campestre*	T23 & this study
Fabales	Fabaceae	*Trifolium dubium*	Sibth.	*NA*	This study
Fabales	Fabaceae	*Trifolium fragiferum*	L.	*NA*	This study
Fabales	Fabaceae	*Trifolium hybridum*	L.	*Trifolium hybridum*	T23
Fabales	Fabaceae	*Trifolium lappaceum*	L.	*NA*	This study
Fabales	Fabaceae	*Trifolium medium*	L.	*Trifolium medium*	T23
Fabales	Fabaceae	*Trifolium pratense*	L.	*Trifolium pratense*	T23 & this study
Fabales	Fabaceae	*Trifolium repens*	L.	*Trifolium repens*	T23 & this study
Fabales	Fabaceae	*Trifolium rubens*	L.	*Trifolium rubens*	T23
Fabales	Fabaceae	*Trifolium spadiceum*	L.	*Trifolium spadiceum*	T23
Alismatales	Juncaginaceae	*Triglochin maritima*	L.	*Triglochin maritima*	T23
Asterales	Asteraceae	*Tripleurospermum inodorum*	(L.) Sch.-Bip	*Tripleurospermum inodorum*	T23
Asterales	Asteraceae	*Tripolium pannonicum*	(Jacq.) Dobrocz.	*Tripolium pannoncium*	T23
Poales	Poaceae	*Triticum aestivum*	L.	*Triticum aestivum*	T23
Poales	Poaceae	*Triticum monococcum*	L.	*Triticum monococcum*	T23
Poales	Poaceae	*Triticum turgidum*	L.	*Triticum polonicum*	T23
Ranunculales	Ranunculaceae	*Trollius chinensis*	Bunge	*Trollius chinensis*	T23
Asterales	Asteraceae	*Trommsdorffia maculata*	(L.) Bernh.	*Hypochoeris maculata*	T23
Brassicales	Tropaeolaceae	*Tropaeolum majus*	L.	*Tropaeolum majus*	T23
Asterales	Asteraceae	*Tussilago farfara*	L.	*Tussilago farfara*	T23
Asterales	Asteraceae	*Tyrimnus leucographus*	(L.) Cass.	*NA*	This study
Fabales	Fabaceae	*Ulex europaeus*	L.	*Ulex europeaus*	T23
Rosales	Ulmaceae	*Ulmus glabra*	Huds.	*Ulmus glabra*	T23
Rosales	Ulmaceae	*Ulmus laevis*	Pall.	*Ulmus laevis*	T23
Rosales	Ulmaceae	*Ulmus minor*	Mill.	*Ulmus minor*	T23
Saxifragales	Crassulaceae	*Umbilicus oppositifolius*	Ledeb.	*Umbilicus oppositifolius*	T23
Saxifragales	Crassulaceae	*Umbilicus rupestris*	(Salisb.) Dandy	*Umbilicus repestris*	T23
Poales	Poaceae	*Urochloa mutica*	(Forssk.) T.Q.Nguyen	*Brachiaria mutica*	T23
Asterales	Asteraceae	*Urospermum dalechampii*	(L.) Scop. ex F.W.Schmidt	*Urospermum dalechampii*	T23
Asterales	Asteraceae	*Urospermum picroides*	(L.) Scop. ex F.W.Schmidt	*Urospermum picroides*	T23
Rosales	Urticaceae	*Urtica dioica*	L.	*Urtica dioica*	T23
Rosales	Urticaceae	*Urtica urens*	L.	*Urtica urens*	T23

Order	Family	Name	Author	Name in Thompson et al. (2023)	Source
Ericales	Ericaceae	*Vaccinium angustifolium*	Aiton	*Vaccinium angustifolium*	T23
Ericales	Ericaceae	*Vaccinium cylindraceum*	Sm.	*Vaccinium cylindraceum*	T23
Ericales	Ericaceae	*Vaccinium macrocarpon*	Aiton	*Vaccinium macrocarpon*	T23
Ericales	Ericaceae	*Vaccinium myrtillus*	L.	*Vaccinium myrtillus*	T23
Ericales	Ericaceae	*Vaccinium ovatum*	Pursh	*Vaccinium ovatum*	T23
Ericales	Ericaceae	*Vaccinium reticulatum*	Sm.	*Vaccinium reticulatum*	T23
Ericales	Ericaceae	*Vaccinium uliginosum*	L.	*Vaccinium uliginosum*	T23
Ericales	Ericaceae	*Vaccinium vitis-idaea*	L.	*Vaccinium vitis-idaea*	T23
Dipsacales	Caprifoliaceae	*Valeriana excelsa*	Poir.	*Valeriana excelsa*	T23
Dipsacales	Caprifoliaceae	*Valeriana officinalis*	L.	*Valeriana officinalis*	T23
Dipsacales	Caprifoliaceae	*Valeriana sambucifolia*	J.C.Mikan	*Valeriana sambucifolia*	T23
Dipsacales	Caprifoliaceae	*Valerianella eriocarpa*	Desv.	*NA*	This study
Dipsacales	Caprifoliaceae	*Valerianella locusta*	(L.) Laterr.	*Valerianella locusta*	T23
Lamiales	Scrophulariaceae	*Verbascum phoeniceum*	L.	*Verbascum phoeniceum*	T23
Lamiales	Scrophulariaceae	*Verbascum thapsus*	L.	*Verbascum thapsus*	T23
Lamiales	Verbenaceae	*Verbena bonariensis*	L.	*Verbena bonariensis*	T23
Lamiales	Verbenaceae	*Verbena hastata*	L.	*Verbena hastata*	T23
Lamiales	Verbenaceae	*Verbena litoralis*	Kunth	*Verbena litoralis*	T23
Lamiales	Verbenaceae	*Verbena rigida*	Spreng.	*Verbena rigida*	T23
Lamiales	Plantaginaceae	*Veronica agrestis*	L.	*Veronica agrestis*	T23
Lamiales	Plantaginaceae	*Veronica arvensis*	L.	*Veronica arvensis*	T23
Lamiales	Plantaginaceae	*Veronica beccabunga*	L.	*Veronica beccabunga*	T23
Lamiales	Plantaginaceae	*Veronica chamaedrys*	L.	*Veronica chamaedrys*	T23
Lamiales	Plantaginaceae	*Veronica hederifolia*	L.	*Veronica hederifolia*	T23
Lamiales	Plantaginaceae	*Veronica longifolia*	L.	*Veronica longifolia*	T23
Lamiales	Plantaginaceae	*Veronica officinalis*	L.	*Veronica officinalis*	T23
Lamiales	Plantaginaceae	*Veronica peduncularis*	M.Bieb.	*Veronica peduncularis*	T23
Lamiales	Plantaginaceae	*Veronica peregrina*	L.	*Veronica peregrina*	T23
Lamiales	Plantaginaceae	*Veronica persica*	Poir.	*Veronica persica*	T23
Lamiales	Plantaginaceae	*Veronica pinguifolia*	Hook.fil.	*Veronica pinguifolia*	T23
Lamiales	Plantaginaceae	*Veronica plebeia*	R.Br.	*Veronica plebeia*	T23
Lamiales	Plantaginaceae	*Veronica rakaiensis*	J.B.Armstr.	*Hebe rakaiensis*	T23
Lamiales	Plantaginaceae	*Veronica salicifolia*	G.Forst.	*Hebe salicifolia*	T23
Lamiales	Plantaginaceae	*Veronica serpyllifolia*	L.	*Veronica serpyllifolia*	T23
Lamiales	Plantaginaceae	*Veronica spicata*	L.	*Veronica spicata*	T23
Lamiales	Plantaginaceae	*Veronica urticifolia*	Jacq.	*Veronica urticifolia*	T23
Dipsacales	Viburnaceae	*Viburnum bodnantense*	Viburnum ×bodnantense Aberc. ex Stearn	*Viburnum x bodnantense*	T23
Dipsacales	Viburnaceae	*Viburnum carlcephalum*	Viburnum ×carlcephalum Burkwood	*Viburnum x carlcephalum*	T23
Dipsacales	Viburnaceae	*Viburnum lantana*	L.	*Viburnum lantana*	T23
Dipsacales	Viburnaceae	*Viburnum opulus*	L.	*Viburnum opulus*	T23
Dipsacales	Viburnaceae	*Viburnum sargentii*	Koehne	*Viburnum sargentii*	T23
Dipsacales	Viburnaceae	*Viburnum tinus*	L.	*Viburnum tinus*	T23
Fabales	Fabaceae	*Vicia cracca*	L.	*Vicia cracca*	T23
Fabales	Fabaceae	*Vicia faba*	L.	*Vicia faba*	T23 & this study
Fabales	Fabaceae	*Vicia hirsuta*	(L.) Gray	*Vicia hirsuta*	T23
Fabales	Fabaceae	*Vicia melanops*	Sm.	*Vicia melanops*	T23
Fabales	Fabaceae	*Vicia sativa*	L.	*Vicia sativa*	T23 & this study
Fabales	Fabaceae	*Vicia sepium*	L.	*Vicia sepium*	T23
Fabales	Fabaceae	*Vicia tetrasperma*	(L.) Schreb.	*Vicia tetrasperma*	T23
Fabales	Fabaceae	*Vicia villosa*	Roth	*Vicia villosa Roth*	T23
Gentianales	Apocynaceae	*Vinca major*	L.	*Vinca major*	T23
Gentianales	Apocynaceae	*Vinca minor*	L.	*Vinca minor*	T23
Malpighiales	Violaceae	*Viola canina*	L.	*Viola canina*	T23
Malpighiales	Violaceae	*Viola epipsila*	Ledeb.	*Viola epipsila*	T23
Malpighiales	Violaceae	*Viola odorata*	L.	*Viola odorata*	T23
Malpighiales	Violaceae	*Viola palustris*	L.	*Viola palustris*	T23
Malpighiales	Violaceae	*Viola riviniana*	Rchb.	*Viola riviniana*	T23
Malpighiales	Violaceae	*Viola tricolor*	L.	*Viola tricolor*	T23
Malpighiales	Violaceae	*Viola wittrockiana*	Viola ×wittrockiana Gams	*Viola x wittrockiana*	T23
Apiales	Apiaceae	*Visnaga daucoides*	Gaertn.	*Ammi visnaga*	T23
Vitales	Vitaceae	*Vitis vinifera*	L.	*Vitis vinifera*	T23
Malvales	Thymelaeaceae	*Wikstroemia phillyreifolia*	A.Gray	*Wikstroemia phillyreifolia*	T23
Asterales	Asteraceae	*Wyethia amplexicaulis*	(Nutt.) Nutt.	*Wyethia amplexicaulis*	T23

Order	Family	Name	Author	Name in Thompson et al. (2023)	Source
Apiales	Apiaceae	*Xanthoselinum alsaticum*	(L.) Schur	*Peucedanum alsaticum*	T23
Alismatales	Araceae	*Zantedeschia aethiopica*	(L.) Spreng.	*Zantedeschia aethiopica*	T23
Poales	Poaceae	*Zea mays*	L.	*Zea mays*	T23

References cited in this Appendix

1. Thompson V, Harkin C, Stewart AJA. The most polyphagous insect herbivore? Host plant associations of the Meadow spittlebug, *Philaenus spumarius* (L.). Bossart JL, editor. PLoS ONE. 2023;18: e0291734. doi:10.1371/journal.pone.0291734
2. GBIF.org. GBIF Home Page. Available from: https://www.gbif.org. Nov 2023.
3. Schneider FD. Package "traitdataform." 2018. Available: https://ecologicaltraitdata.github.io/traitdataform/
