# Supplementary material for: Insect-habitat-plant interaction networks provide guidelines to mitigate the risk of transmission of Xylella fastidiosa to grapevine in Southern France
Source: PLoS One. 2025 Sep 15;20(9):e0332344. doi: 10.1371/journal.pone.0332344 (PMC12435670; doi:10.1371/journal.pone.0332344)
Supplement: S1 Appendix — (ZIP) [file pone.0332344.s001.zip › S12_Appendix.pdf]

Appendix S12: additional data on insect-plant interaction network

Additionally to the regular sampling protocol for nymphs, other samples were made opportunistically, when we spotted spittles on infrequent plants. The networks below display all interactions observed, including those reported in Appendix S5, and additional observations.

Four new plant families appear in these networks:

- Dennstaedtiaceae, a family of ferns, represented here by *Pteridium aquilinum*;
- Fagaceae, represented by *Quercus robur* (the spittle was found on a seedling);
- Cyperaceae, represented by *Carex hirta* and
- Euphorbiaceae represented by *Euphorbia helioscopia*.

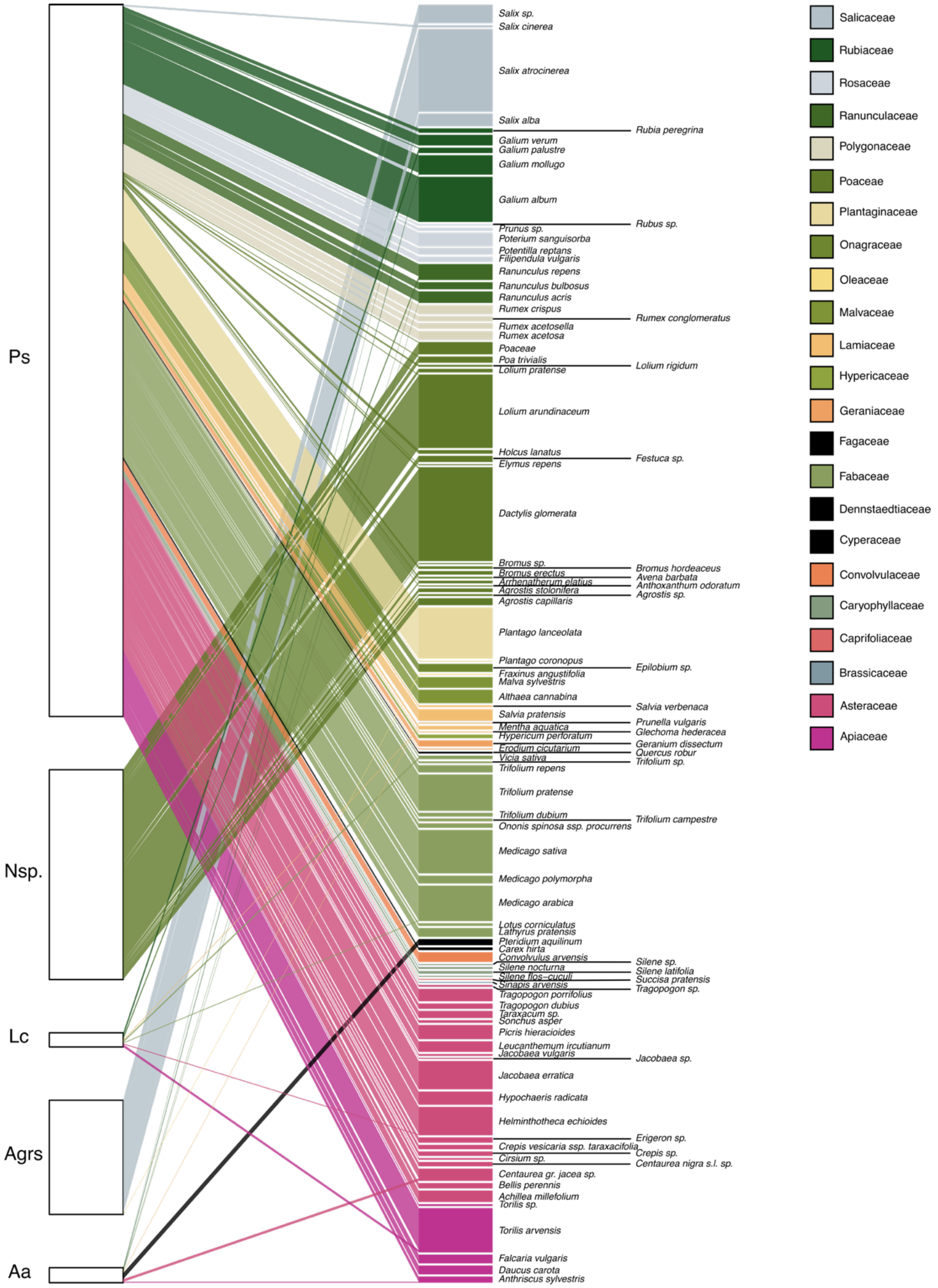

Figure S12.1. Insect plant network in NAQ at the nymph stage (spring 2021), including regular and additional opportunistic observations. The colors are the same as previously, the four new plant families are depicted in black to stand out (see above for the correspondence species-family). Insect species are abbreviated as follows Aa: *Aphrophora alni*, Agrs: *Aphrophora* grp. *salicina*, Cv: *Cicadella viridis*, Lc: *Lepyronia coleoptrata*, Nc: *Neophilaenus campestris*, Nl: *Neophilaenus lineatus*, Nsp.: *Neophilaenus* sp. and Ps: *Philaenus spumarius*.

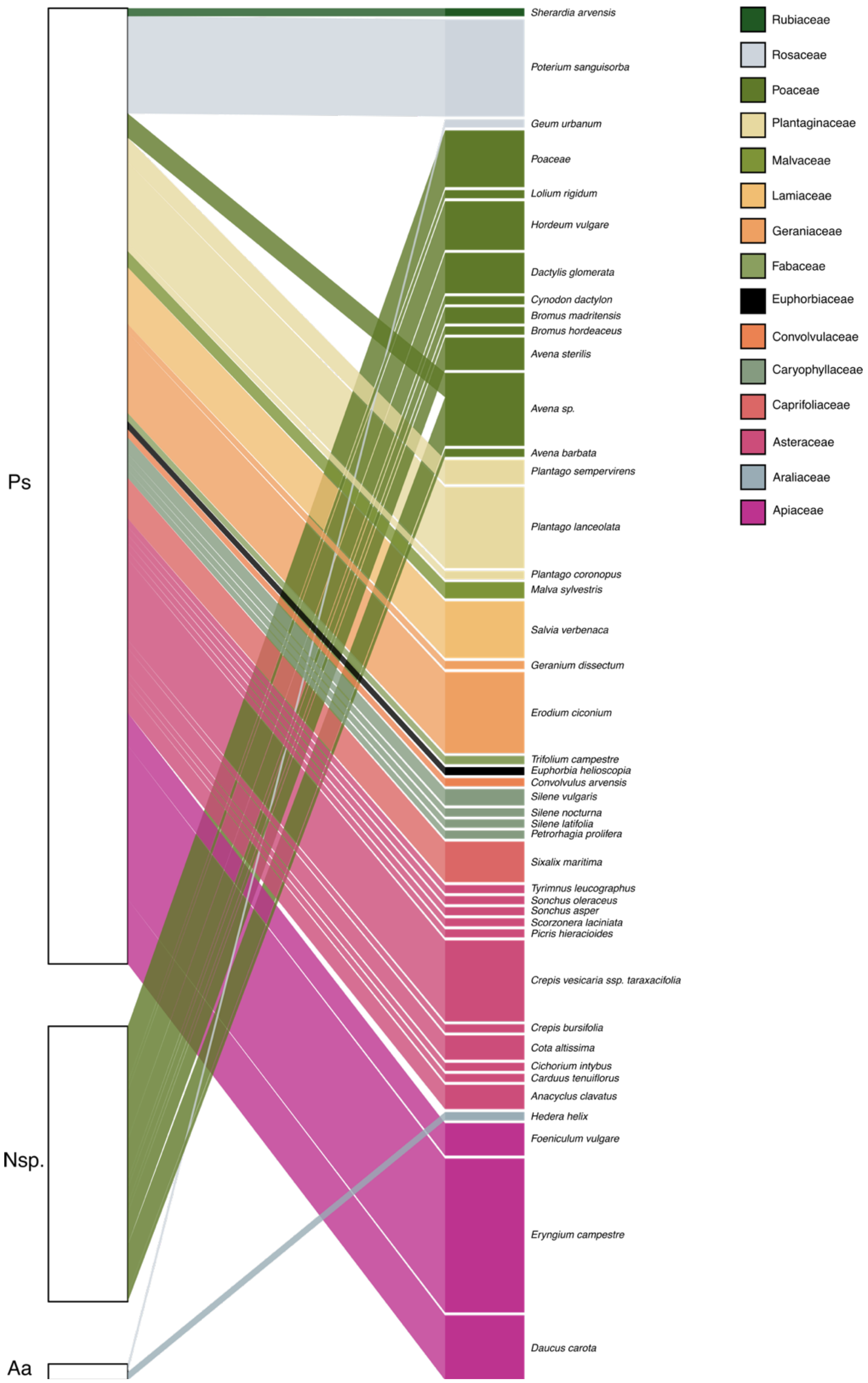

Figure S12.2. Insect plant network in OCC at the nymph stage (spring 2021), including regular and additional opportunistic observations. The colors are the same as previously, the four new plant families are depicted in black to stand out (see above for the correspondence species-family). Insect species are abbreviated as follows Aa: *Aphrophora alni*, Agrs: *Aphrophora* grp. *salicina*, Cv: *Cicadella viridis*, Lc: *Lepyronia coleoptrata*, Nc: *Neophilaenus campestris*, Nl: *Neophilaenus lineatus*, Nsp.: *Neophilaenus* sp. and Ps: *Philaenus spumarius*.

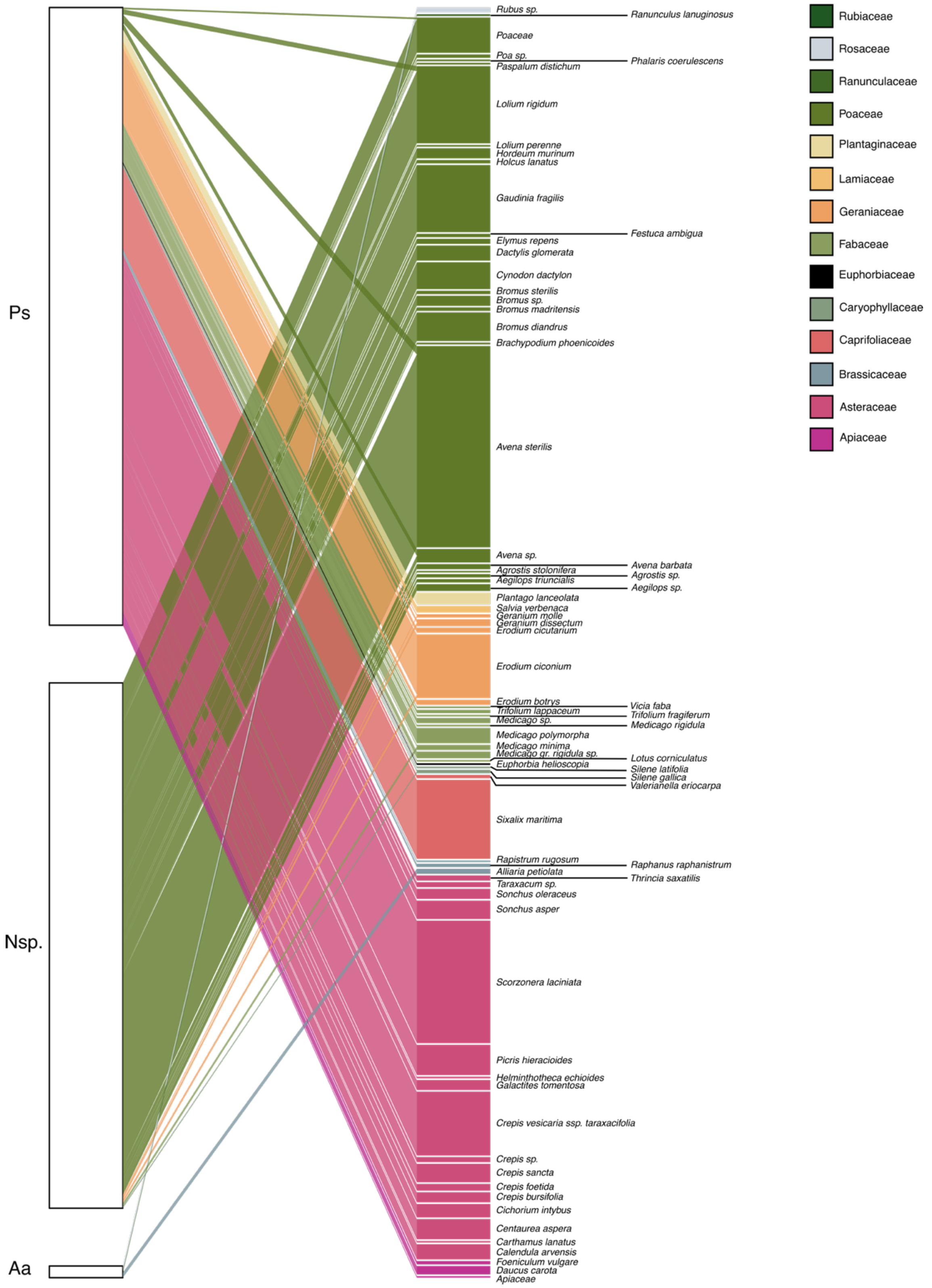

Figure S12.3. Insect plant network in PACA at the nymph stage (spring 2021), including regular and additional opportunistic observations. The colors are the same as previously, the four new plant families are depicted in black to stand out (see above for the correspondence species-family). Insect species are abbreviated as follows Aa: *Aphrophora alni*, Agrs: *Aphrophora* grp. *salicina*, Cv: *Cicadella viridis*, Lc: *Lepyronia coleoptrata*, Nc: *Neophilaenus campestris*, Nl: *Neophilaenus lineatus*, Nsp.: *Neophilaenus* sp. and Ps: *Philaenus spumarius*.

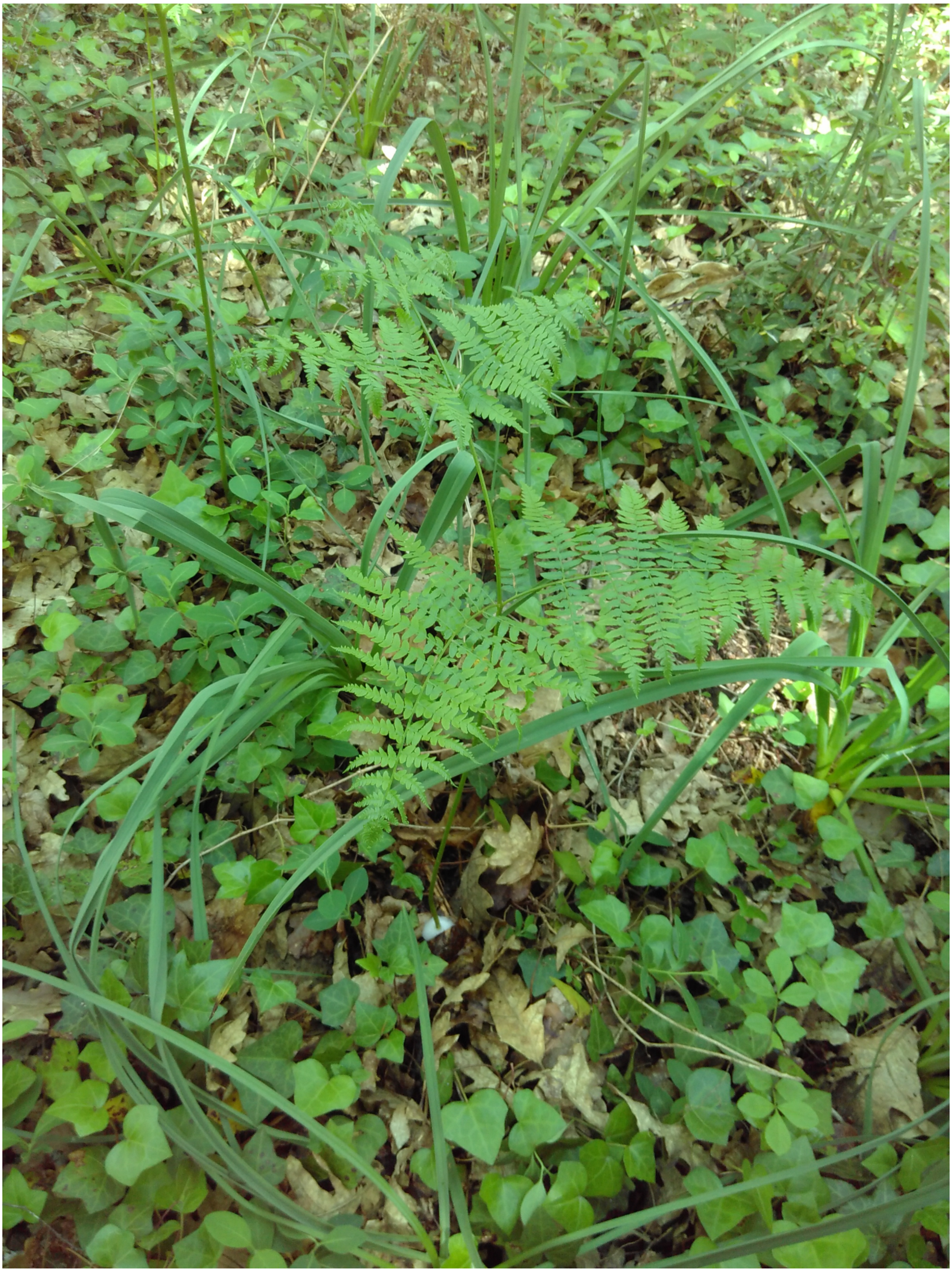

Figure S12.4. ***A. alni*** **spittle on** ***P. aquilinum.***
