# Supplementary figures and images for: Insect-habitat-plant interaction networks provide guidelines to mitigate the risk of transmission of Xylella fastidiosa to grapevine in Southern France
Source: PLoS One. 2025 Sep 15;20(9):e0332344. doi: 10.1371/journal.pone.0332344 (PMC12435670; doi:10.1371/journal.pone.0332344)

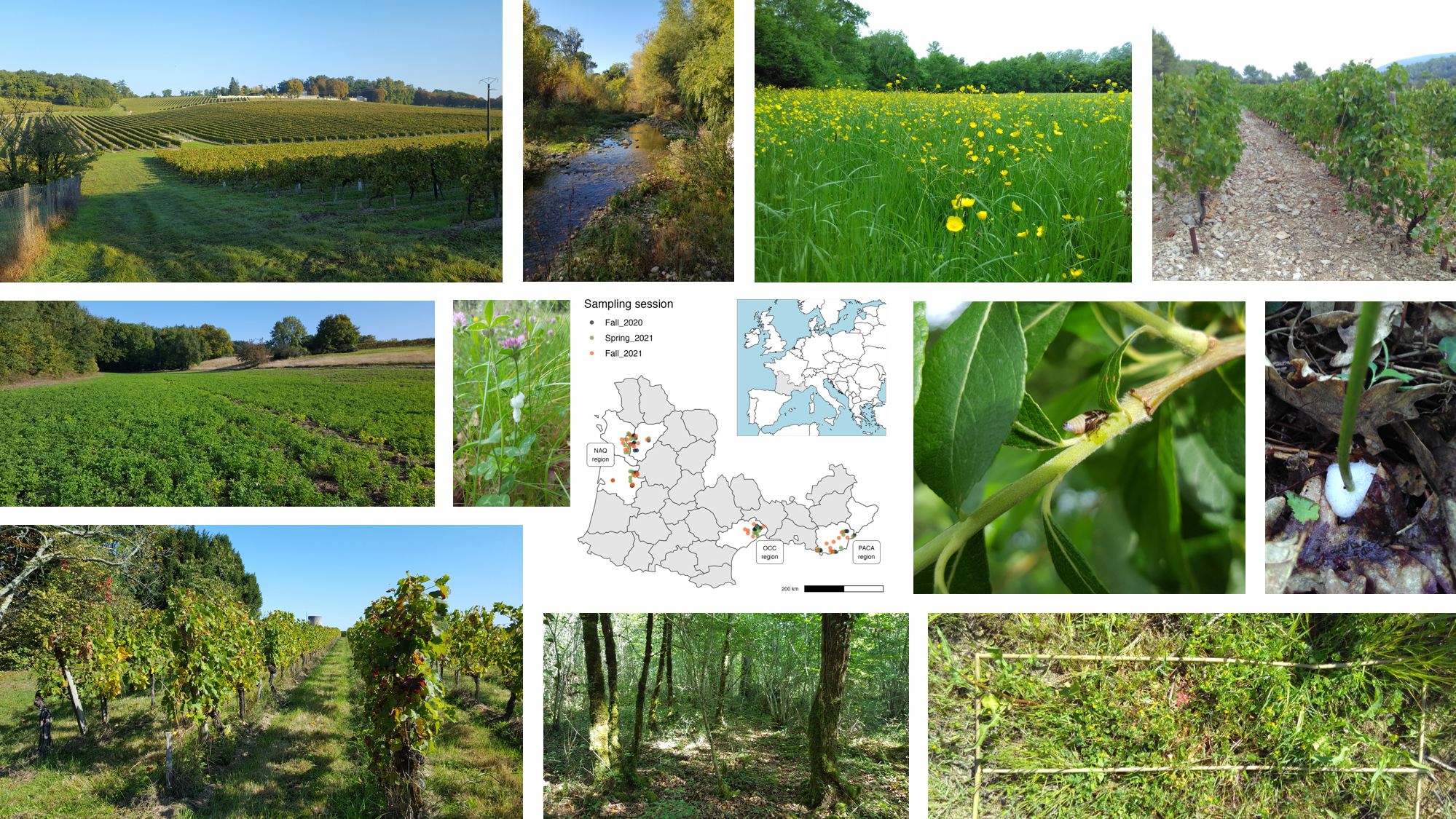

Supplement: S2 — (PNG) [file pone.0332344.s002.png]
