# Supplementary material for: Insect-habitat-plant interaction networks provide guidelines to mitigate the risk of transmission of Xylella fastidiosa to grapevine in Southern France
Source: PLoS One. 2025 Sep 15;20(9):e0332344. doi: 10.1371/journal.pone.0332344 (PMC12435670; doi:10.1371/journal.pone.0332344)
Supplement: Attachment — Submitted filename: Response to Reviewers.docx [file pone.0332344.s004.docx]

**PONE-D-24-38713**

**Insect-habitat-plant interaction networks provide guidelines to mitigate the risk of transmission of *Xylella fastidiosa* to grapevine in Southern France**

**Journal Requirements:**

When submitting your revision, we need you to address these additional requirements.

1. Please ensure that your manuscript meets PLOS ONE's style requirements, including those for file naming. The PLOS ONE style templates can be found at

[https://journals.plos.org/plosone/s/file?id=wjVg/PLOSOne_formatting_sample_main_body.pdf](https://track.editorialmanager.com/CL0/https:%2F%2Fjournals.plos.org%2Fplosone%2Fs%2Ffile%3Fid=wjVg%2FPLOSOne_formatting_sample_main_body.pdf/1/010f0195edfed723-8ed4d0fd-35c7-40e3-a95c-f9398e0f8b0f-000000/WFE0VWvtp274ayOaCG8pdETuUsrP8JhBigl6hlyHD5s=204) and

[https://journals.plos.org/plosone/s/file?id=ba62/PLOSOne_formatting_sample_title_authors_affiliations.pdf](https://track.editorialmanager.com/CL0/https:%2F%2Fjournals.plos.org%2Fplosone%2Fs%2Ffile%3Fid=ba62%2FPLOSOne_formatting_sample_title_authors_affiliations.pdf/1/010f0195edfed723-8ed4d0fd-35c7-40e3-a95c-f9398e0f8b0f-000000/DQN1U5GnfXp_ZSYCpXOvUzDJK9tpriG2OAyefPcDxbg=204)

> We confirm that our manuscript meets PLOS ONE’s style requirements.

2. In your Methods section, please provide additional information regarding the permits you obtained for the work. Please ensure you have included the full name of the authority that approved the field site access and, if no permits were required, a brief statement explaining why.

> No regulation applies to the collection of insect vectors (proven or putative) of *Xylella fastidiosa* in France. None of our sampling sites falls within a protected area and no restriction applied to insect collection in our sampling sites. Thus insect sampling did not require any permission from French authorities. Farmers were contacted before field visits through the network of our funder, Hennessy, and granted individually access to the fields.

A sentence was added in the M&M section to mention that.

3. We note that Figure 1 in your submission contain [map/satellite] images which may be copyrighted. All PLOS content is published under the Creative Commons Attribution License (CC BY 4.0), which means that the manuscript, images, and Supporting Information files will be freely available online, and any third party is permitted to access, download, copy, distribute, and use these materials in any way, even commercially, with proper attribution. For these reasons, we cannot publish previously copyrighted maps or satellite images created using proprietary data, such as Google software (Google Maps, Street View, and Earth). For more information, see our copyright guidelines: [http://journals.plos.org/plosone/s/licenses-and-copyright](http://track.editorialmanager.com/CL0/http:%2F%2Fjournals.plos.org%2Fplosone%2Fs%2Flicenses-and-copyright/1/010f0195edfed723-8ed4d0fd-35c7-40e3-a95c-f9398e0f8b0f-000000/Xt8259NnjoYHqTZyWob22F7GRaWJF1mIS6A-l6Onp9k=204).

We require you to either (1) present written permission from the copyright holder to publish these figures specifically under the CC BY 4.0 license, or (2) remove the figures from your submission:

    a. You may seek permission from the original copyright holder of Figure 1 to publish the content specifically under the CC BY 4.0 license.

We recommend that you contact the original copyright holder with the Content Permission Form (<http://journals.plos.org/plosone/s/file?id=7c09/content-permission-form.pdf>) and the following text:

“I request permission for the open-access journal PLOS ONE to publish XXX under the Creative Commons Attribution License (CCAL) CC BY 4.0 (<http://creativecommons.org/licenses/by/4.0/>). Please be aware that this license allows unrestricted use and distribution, even commercially, by third parties. Please reply and provide explicit written permission to publish XXX under a CC BY license and complete the attached form.”

Please upload the completed Content Permission Form or other proof of granted permissions as an "Other" file with your submission.

In the figure caption of the copyrighted figure, please include the following text: “Reprinted from [ref] under a CC BY license, with permission from [name of publisher], original copyright [original copyright year].”

     b. If you are unable to obtain permission from the original copyright holder to publish these figures under the CC BY 4.0 license or if the copyright holder’s requirements are incompatible with the CC BY 4.0 license, please either i) remove the figure or ii) supply a replacement figure that complies with the CC BY 4.0 license. Please check copyright information on all replacement figures and update the figure caption with source information. If applicable, please specify in the figure caption text when a figure is similar but not identical to the original image and is therefore for illustrative purposes only.

The following resources for replacing copyrighted map figures may be helpful:

USGS National Map Viewer (public domain): <http://viewer.nationalmap.gov/viewer/>

The Gateway to Astronaut Photography of Earth (public domain): <http://eol.jsc.nasa.gov/sseop/clickmap/>

Maps at the CIA (public domain): <https://www.cia.gov/library/publications/the-world-factbook/index.html> and <https://www.cia.gov/library/publications/cia-maps-publications/index.html>

NASA Earth Observatory (public domain): <http://earthobservatory.nasa.gov/>

Landsat: <http://landsat.visibleearth.nasa.gov/>

USGS EROS (Earth Resources Observatory and Science (EROS) Center) (public domain): [http://eros.usgs.gov/#](http://eros.usgs.gov/)

Natural Earth (public domain): <http://www.naturalearthdata.com/>

> No copyrighted images were used to build the map in Fig 1. Country contours were displayed using Natural Earth (version 5.1.1) under a CC0 licence (<https://www.naturalearthdata.com/about/terms-of-use/>), and French departments were displayed using ADMIN EXPRESS database from the National Institute of Geography (IGN), freely available under a CC BY licence (<https://www.data.gouv.fr/fr/datasets/admin-express-admin-express-cog-admin-express-cog-carto-admin-express-cog-carto-pe/#/community-reuses>).

A simplified version of this sentence was added to Figure 1 caption, and to the captions of other maps in Appendices.

**Comments to the Author**

1. Is the manuscript technically sound, and do the data support the conclusions?

The manuscript must describe a technically sound piece of scientific research with data that supports the conclusions. Experiments must have been conducted rigorously, with appropriate controls, replication, and sample sizes. The conclusions must be drawn appropriately based on the data presented.

Reviewer #1: Partly

2. Has the statistical analysis been performed appropriately and rigorously?

Reviewer #1: I Don't Know

3. Have the authors made all data underlying the findings in their manuscript fully available?

The PLOS Data policy requires authors to make all data underlying the findings described in their manuscript fully available without restriction, with rare exception (please refer to the Data Availability Statement in the manuscript PDF file). The data should be provided as part of the manuscript or its supporting information, or deposited to a public repository. For example, in addition to summary statistics, the data points behind means, medians and variance measures should be available. If there are restrictions on publicly sharing data—e.g. participant privacy or use of data from a third party—those must be specified.

Reviewer #1: No

> We are sorry that it was probably not clearly mentioned, but yes, **all datasets and R codes used for data analyses and for computing figures were deposited on a public repository**. Precisely, the dataset and R code (for both figures and statistics) were deposited on the 12^th^ of May 2024 on Zenodo (<https://zenodo.org/records/11181990>). This is mentioned on the appropriate section “Statistical and data reporting” of the manuscript. This has been viewed 67 times and downloaded 36 times.

4. Is the manuscript presented in an intelligible fashion and written in standard English?

PLOS ONE does not copyedit accepted manuscripts, so the language in submitted articles must be clear, correct, and unambiguous. Any typographical or grammatical errors should be corrected at revision, so please note any specific errors here.

Reviewer #1: Yes

**Reviewers' comments: Review Comments to the Author**

Reviewer #1:

In this study, the authors examined insect-habitat and insect-plant networks in three French wine-growing regions to identify xylem feeder habitats and specialization. Surveys across 92 landscapes showed meadows and alfalfa fields hosted the largest communities, while grapevines had few. *Philaenus spumarius*, *Aphrophora alni*, *Lepyronia coleoptrata*, and *Cicadella viridis* were generalists, while *Aphrophora* grp. *salicina* was a strict specialist on riparian forests and Salicaceae. Screening 1,017 insects found no *Xylella fastidiosa*, suggesting it is not widespread, while providing key insights into potential vector ecology. The study is both innovative and valuable, particularly due to its extensive site coverage and novel sampling design. A key strength is the simultaneous collection of insect-habitat and insect-plant network data alongside *Xylella fastidiosa* (Xf) screening, providing a comprehensive ecological perspective. However, several major concerns need to be addressed.

Clarity: The manuscript requires improvement in multiple sections, especially in the descriptions of network and statistical analyses. Additionally, the Introduction should better contextualize the study within existing research.

Sampling: A significant limitation is that sites were sampled only once, and adult insects were monitored exclusively in autumn, which may impact the completeness of the dataset and trend interpretation (see specific comments below).

Analysis: The analytical approach is difficult to follow, and a clearer explanation is needed to ensure full transparency for the reader. Additionally, potential methodological issues should be addressed, including the confounding effects of insect species and habitat characteristics in specialization analyses, as well as concerns regarding model construction and simplification procedures.

\#####
**Introduction.** Interesting and well written. Besides minor comments, I would suggest the authors to orderly present the different *Xf* subspecies, their main problematics in terms of plant protection, their main vectors and relative characteristics. I think this would help a non-specialized audience to better follow the study.

> We strengthened the presentation of *Xf* subspecies, of their main vectors, and of the threats they pose for plant health.

L59 New reference: Cornara, D., Boscia, D., D’Attoma, G., Digiaro, M., Ligorio, A. M., Loconsole, G., ... & Saponari, M. (2024). An integrated strategy for pathogen surveillance unveiled Xylella fastidiosa ST1 outbreak in hidden agricultural compartments in the Apulia region (Southern Italy). European Journal of Plant Pathology, 1-9.

> We added this new reference.

L67 and 82-84 Authors should better highlight the previous studies that adopted similar approach (species-habitat network) for Xf vectors in Europe (e.g., Cappellari et al. 2022). So far, Cappellari et al. (2022) is only mention to support the statement “Large populations of P. spumarius were also reported in grasslands”, which is reductive. For example, in their study Cappellari et al. found that *Philaenus spumarius* was mostly collected in olive groves but it acted as a super-generalist species, occupying all the habitats (this seems to match some of the authors’ findings). I suggest the authors to better highlight the innovative aspects of their work on the base of past studies.

> We have more clearly described the super-generalist status of *P. spumarius*, both on host plants and habitats, as described in the results of previous studies (Bodino et al., 2019; Capellari et al., 2022; Villa et al., 2020), and we have also more clearly described what our study contributes to the subject.

I would suggest authors to more clearly present the different *Xf* subspecies, their relevance for crop protection (which plant species might be strongly affected?) and the relative main vectors and their characteristics (as shortly done at L97-101). Authors could also briefly present the known distribution for the main Xf vetcors in Europe. This work will be very interesting for Xf specialists but it could also appeal a broader, less knowledgeable audience.

> We added description of the five subspecies of *Xf* and their economic impacts, as well as more details on vectors (species and distribution).

L105 New reference: Cornara, D., Boscia, D., D’Attoma, G., Digiaro, M., Ligorio, A. M., Loconsole, G., ... & Saponari, M. (2024). An integrated strategy for pathogen surveillance unveiled Xylella fastidiosa ST1 outbreak in hidden agricultural compartments in the Apulia region (Southern Italy). European Journal of Plant Pathology, 1-9.

> We added this new reference.

\#####
**Materials and Methods:** This section requires significant improvement in clarity and completeness. Additional details should be provided to enhance transparency, ensuring that the methodology can be fully understood and replicated. Specific aspects, such as sampling procedures, data collection protocols, and analytical methods, should be described more precisely to strengthen the study’s rigor.

> We thank the reviewer for these suggestions to improve the clarity of the methodology. We have implemented the suggested changes.

L130 Authors should indicate which type of habitats were considered and how different patches were selected (one habitat type per buffer? based on relative abundance of each habitat?).

> We provided clarifications about the distribution of land cover types within the buffer and the distribution of habitat types within the sites.

L148-150 I am concerned about this point. Authors sampled adults in two years, only in autumn, indicating that unpublished phenological data in the South and in the West of France show peak densities of adults in the autumn. First, I think authors should more strongly support this statement. Adult abundance peak often occurs in spring (Bodino et al. 2019, Hasbroucq et al. 2020, Cappellari et al. 2022) although high densities have been observed later along the season (Bodino et al. 2021). Can authors present in the supplementary (very aggregated) data on vector phenology in France, in order to support their claims (without losing the possibility of future publications)? Again I think this is important, because, if this is not the case, authors might have missed the real population peak.

> Thank you very much for raising that issue. We analyzed more thoroughfully our phenological data and we now show it in Appendix S2. We show two peaks of abundance in the year, one in mid-May / early June, and a second one in late September / October. As you mention, the highest densities were actually recorded in mid-May rather than in the autumn for aphrophorids as a whole. For *P. spumarius* alone, the peak also occurred in mid-May in OCC region, but two equivalent peaks were recorded in NAQ region, in mid-May and in late September.

We conclude that we sampled adults at a time that is meaningful in their population dynamic because they are very abundant. But we did not sample them at their highest densities, except for *P. spumarius* in NAQ region, as this species seems to display two similar peaks in the year in that place.

We changed the main text correspondingly. We state that sampling periods were pertinent regarding insect phenology, but that missing the peak density of adults (partly for logistic reasons, the financed project started in the summer 2020) is a limitation of our study.

Second, with only one season sampled, authors cannot be sure about the vector habitat preferences. In fact, these vectors are mobile, and they might use habitat differently in different seasons. For example, Cappellari et al. 2022 shows that grasslands are preferred by *P. spumarius* adults disproportionally more in spring than in autumn. In other words, authors can argue about habitat preference in autumn, and we don’t know what happen in spring (generally a very important season for these vectors, when actual dispersion may occur).

> Indeed. We are now more careful when talking about vector preferences. When mentioning adult preferences (for habitats) we reformulated for “autumnal adults preferences”. We also discuss this limitation in details at the beginning of the section "Most xylem feeders are generalists" of the Discussion.

Finally, although the total number of sites is high, each subsite in each site was sampled only once (L160): this type of snapshot sampling does not allow to properly capture local variability, increasing uncertainty. I think authors should better justify their choice and acknowledge the potential limitation of the study.

> Here we disagree about the potential limitations induced by the fact that we chose to change the sampling sites on each session. We agree that we do not capture local variability with our sampling design. Focusing on this would have required a larger number of sites per buffer and very frequent field visits to monitor population transfers between habitats if they occur. This was not an ambition of our sampling design. Our sampling design is meant to produce as many replications as possible in time and space in order to capture most of the variability in insect habitat preferences in three contrasted regions.

We clarified this section about this point.

Note that for the phenological survey we remained on the same sites throughout the survey because the sampling design was meant to build a longitudinal study, with short time steps between each sample occasions. In that particular example, we wanted to remove the extra-variability linked with a change of site. We also clarified this point in S2 Appendix.

Bodino, N., Cavalieri, V., Pegoraro, M., Altamura, G., Canuto, F., Zicca, S., ... & Bosco, D. (2021). Temporal dynamics of the transmission of Xylella fastidiosa subsp. pauca by Philaenus spumarius to olive plants. Entomologia Generalis, 1-17.

Bodino, N., Demichelis, S., Simonetto, A., Volani, S., Saladini, M. A., Gilioli, G., & Bosco, D. (2021). Phenology, seasonal abundance, and host-plant association of spittlebugs (Hemiptera: Aphrophoridae) in vineyards of Northwestern Italy. Insects, 12(11), 1012.

Hasbroucq, S., Casarin, N., Czwienczek, E., Bragard, C., & Grégoire, J. C. (2020). Distribution, adult phenology and life history traits of potential insect vectors of Xylella fastidiosa in Belgium. Belg. J. Entomol, 92, 1-21.

L150-151 I don’t think that changing buffers each year completely solve the problem of pseudoreplication. The site selection as described so far does not exclude spatial/temporal dependence among sites. First, new buffers should have been selected to a sufficient distance to present and past buffers (2km distance between two centroids) in order to be confident that different vector populations were sampled. Also, the effect of “space” and “time” is now confounded (differences among years depend on time or on different characteristics of sites sampled?).

> In response to this we computed the shortest distance between buffer contours per session, and for the whole study. This was added to the text: 90% of buffers were at least 1.1 km away from each other (median shortest distance = 3.3 km). For each sampling session independently, this distance is always >= 1.1 km, with a median shortest distance of 6.1 km. As most studies to date indicate dispersal at several hundreds of meters, exceptionally more than 1 km in a spittlebug’s life, we can be relatively confident that we indeed sampled different populations, both in space and time.

L160 subsite here coincide habitat right?

> Yes, a subsite always belongs to one habitat. This sentence was clarified. We also clarified the paragraph describing the sites and subsites to explain more clearly that a site belongs to a particular “Land Cover Type”, and that a subsite belongs to a particular “Habitat”.

L163-164 not clear, I suggest rephrasing.

> We clarified the sentence.

L167-174 Please provide references to support this approach. Changing quadrat area should theoretically allow to weight vector abundance on vegetation abundance. However it is not clear to me if this was done systematically and consistently across habitat (stratus by site). How authors would interpret the same amount of vectors in e.g., upper strata of vineyards vs. lower strata of meadows? Please elaborate on this (and provide references).

> This was done consistently across habitat: 3 sizes of quadrat were used, 1mx0,25m for all lower strata sites quadrats, and 5mx0,5m for upper strata sites of vineyards, and 2mx0,5m for all other upper strata sites. We used these 3 shapes of quadrats in order to standardize the measure of insect abundance (or insect density) by foliage density, which was approximated by the time needed to examine the foliage when no spittle were sampled. Vine foliage had a specific quadrat dimension among the "upper strata sites" because, during the sampling season, the grapevine was just in the leaf growth stage and it was necessary to increase the sampling area to obtain enough foliage for examination.

The only reference we can use here is the surface sampled for herbaceous vegetation, used as a starting point to adapt our method to different habitats.

L 187 So, in the selected sites, were all *P. spumarius* and *N. campestris* captured also tested?

> For financial reasons we could not test all *P. spumarius* and *N. campestris*. We clarified this in the sentence.

**Network analysis.** I found the description of network analysis quite confusing and difficult to follow. As mentioned below, I suggest the authors to first explain the overall approach (e.g., it could be useful to mention at the beginning that authors want to separately explore insect-habitat, insect-plant families and insect-plant species networks). Then authors should clearly state for each type of analysis which data were used (adults vs nymphs) the resolution/aggregation of the data (how many networks?), the type of metrics extracted. Please, considering the complexity of the study (in terms of structure) and analyses (in terms of different type of aggregation/metrics used), I strongly suggest the authors to help the reader briefly explaining why they selected that particular metric/strategy and how it works (e.g., Lami et al. 2020 did a very good job in breaking down the analytical process and explaining each metric used).

> We have restructured the Ecological Data Analysis section to more clearly explain the overall approach to the 3 types of networks constructed, then the level of data aggregation, and finally the calculation of specialization metrics. We have briefly defined each selected metrics and explained how they work.

Also, I have another doubt regarding the analyses: authors combine two types of network: species habitat and species-plant networks. My doubt is that since constructing insect-plant interaction networks from data collected across different habitats, there is a risk of confounding the effect of insect species with the effect of habitat characteristics, particularly due to the varying plant communities across habitats. This might lead to misinterpretation of dietary specialization, making it difficult to determine whether an insect’s observed feeding behavior is due to intrinsic dietary preferences or simply a reflection of the available plant species in a given habitat. Did authors consider this? In order to disentangle these effects authors might 1) Standardize plant availability across habitats. I think authors attempted to do this, even thought there were still big differences in terms of plant cover across habitats (see my comment above). Also, I don’t think this would solve the problem of different plant diversity across habitat. 2) Compare insect specialization across habitats. Does diet changes with plant availability (suggesting habitat-driven effects)?

> This is right, the observed preference on plant species / families might be simply linked with the abundance of these plants in the habitat in which it thrives. And reciprocally, the observed habitat preference might be due to the contrasted abundance of its preferred host plant/species.

We think that we don’t have the data to disentangle these two types of preferences since both occur simultaneously in the field. We did not conduct an independent botanical survey on the site where we sampled the nymphs of xylem feeder. We agree that this would be a good way to test if links in the networks are simply proportional to plant species/family abundance in the habitat, or skewed in favour of a particular species/family. As for your second idea for statistical analysis, we won’t have the sample size to divide the analysis per habitat.

We acknowledge that this is a limitation of our study and we added this to the Discussion. The best we can do is confront our results to litterature. Our results and the literature suggest that for *A. salicina* the specialization is first at the plant species level, inducing a specialization on the habitat of riparian forests. For *Neophilaenus* spp., it suggests that specialization is first at the plant family level, on Poaceae, inducing a specialization toward habitats that are rich in Poeceae.

L215-218 This is not clear. Were both nymphs and adults considered in this aggregation? Were all the three years aggregated or kept separated? If only 3 networks were generated, it seems like there were not enough replicates to properly analyse specialization. This section should be clearer. Also, are authors talking about species-habitat networks (I guess) or vector-plant network? I see now that this is explained below. I suggest the authors to begin this paragraph clearly presenting the analytical approach adopted here.

> By moving this paragraph at the beginning of the "Ecological data analysis", we believe indeed that the method is clearer. We also made explicit the number of networks calculated. We chose to compute a limited number of big networks (one per region and sampling session), rather than a high number of small networks (e.g. one network per buffer) as small networks are known to be difficult to interpret with usual metrics (see arguments on this in the M&M).

L220 something strange in “including in low connectance networks”.

> We rephrased "low-connectivity networks". We chose to compute the metrics RR and PDI because these metrics remain robust even when network connectance is low, which can happen in small networks or when sampling is incomplete.

L221 what is d’?

> *d’* is a metric defined by Blüthgen et al. (2006). In line with the global clarification of the M&M we now define more precisely *d’* in the maintext.

Blüthgen N, Menzel F, Blüthgen N. Measuring specialization in species interaction networks. BMC Ecol. 2006;6: 9. doi:10.1186/1472-6785-6-9

L22-224. Please, better explain this concept. Also are you sure that ref 53 used the same approach?

> We completed the paragraph to better explain the methodology behind the use of z-transformed metrics.

The z-scores metrics were obtained by comparing observed values to a null distribution generated from 1,000 randomized networks built with preserved marginal totals while randomizing interaction structure. This standardization allows us to assess whether observed specialization deviates from expectations based on species abundances alone, thereby reducing the confounding effects of sampling effort and network size.

Yes, Lami et al. (2020, previously ref 53) used the same standardization approach. We actually found the idea of this standardization procedure precisely in their article. We reformulated to make this clear.

L231-232 I don’t understand what “(resp. plant family or species)” means. Also, why at 233 plants are mentioned when speaking about insect-habitat networks? Please make this part clearer.

> We made this part clearer and removed “(resp. plant family or species)”, that was a shortcut to indicate that the word “habitat”, could be changed for “plant species”, or “plant family”. We now explain the logic for insect-habitat networks, and state that the same logic applies to insect-plant family and insect-plant species networks.

We also mentioned “**insect** species” at l. 233 to avoid confusion with plant species.

L234-235 so 9 networks? Please make number of networks explicit.

> We made explicit the number of networks calculated at the end of the section "Aggregation of the data".

L247 It is not clear to me why “metric” is a predictor here. Shouldn’t each metric be analyzed separately? I might have missed something but it seems to me like analyzing abundance, diversity, evenness in the same model. This would not make sense, so maybe I misunderstood. Please explain and provide appropriate explanation. Also, shouldn’t the model include the year as predictor (even if authors are not interested in the year effect)? I don’t understand what the random factor is…

> All metrics assess “insect specialization”, which is the response variable we want to analyze. Metrics were indeed analyzed separately and detailed results can be found in Appendices 5 and 6 so the reader can dig into different behaviors of the specialization metrics on our dataset. But the aim of our analysis in the main text is to extract the common message from all specialization metrics, hence the fact that we pooled these metrics together. The problem of this is that we artificially inflate the statistical power of the dataset, with multiple assessment of the specialization of the same species, in the same network. This is why we use a random effect on the interaction between insect species, region and sampling session.

Thank you for the comment on the random effect that was poorly described. We now detail the composition of the random effect, which combines the insect species, the region, and the sampling session.

L255-256 I don’t agree with the authors in using this approach. Removing insignificant fixed terms iteratively in GLMMs is a bad practice because it inflates Type I error, increases overfitting, and makes p-values unreliable. Instead of relying solely on statistical significance, models should be built based on ecological reasoning rather than just stepwise selection. A more robust approach is to retain biologically meaningful predictors even if not significant.

> Thank you for mentioning this! We relied on this very common practice (e.g. Calcagno & de Mazancourt , Whittingham et al. 2006), but we see that statisticians indeed advocate against the use of backward variable selection (e.g. Whittingham et al. 2006). We ran the stats again based on this. Please note that the content of the main text did not change, because the backward selection procedure we defined in the former version only applied to one particular model, the one on xylem feeder diversity. In this particular model, keeping the unsignificant effect of “Stage” had no substantial consequence on either p-values of the other effects, or on R^2^. In all other models, all explanatory variables in full models were significant.

Consequently, we updated the description of the statistical method and the result tables in Appendix S5 (backing our method with Whittingham et al. 2006), we reformulated the brief description of the method in the M&M section, we updated Fig 5 (the figure containing the results on xylem feeder diversity) and finally updated the “Xf_continent.Rmd” code uploaded on Zenodo, the shared code used for all statistics and graphics of our article.

Calcagno V, de Mazancourt C. **glmulti** : An *R* Package for Easy Automated Model Selection with (Generalized) Linear Models. J Stat Soft. 2010;34. doi:[10.18637/jss.v034.i12](https://doi.org/10.18637/jss.v034.i12)

Whittingham MJ, Stephens PA, Bradbury RB, Freckleton RP. Why do we still use stepwise modelling in ecology and behaviour? *Journal of Animal Ecology*. 2006;75: 1182–1189. doi:[10.1111/j.1365-2656.2006.01141.x](https://doi.org/10.1111/j.1365-2656.2006.01141.x)

L263 Again, I would include the year as fixed factor. As it is, the model consider your data as collected in 700 independent sites, all collected the same year. This is not the case.

> We agree with this, and this is what was done. Since the buffers and sites were changed on each sampling session, the random effects on buffer ID and on site ID include the sampling session. The precision was added to the text.

L265-266 Including a random factor with only two levels in a GLMM is problematic because it provides insufficient data to estimate random variance accurately, often leading to variance estimates close to zero. This can cause inflated Type I error rates, making fixed effect estimates appear more significant than they actually are. Instead of treating a two-level factor as random, it is better to model it as a fixed effect or ensure more levels are included for proper variance estimation.

> We fully agree, and our random effects have indeed more than two levels. As indicated in Table 1, the number of buffers (i.e. the number of random effect levels for “Buffer”) is 92, and the number of sites (i.e. the number of random effect levels for “Site”) is 700.

Bolker, B. M., Brooks, M. E., Clark, C. J., Geange, S. W., Poulsen, J. R., Stevens, M. H. H., & White, J. S. S. (2009). Generalized linear mixed models: a practical guide for ecology and evolution. Trends in Ecology & Evolution, 24(3), 127-135. <https://doi.org/10.1016/j.tree.2008.10.008>

Gelman, A., & Hill, J. (2007). Data Analysis Using Regression and Multilevel/Hierarchical Models. Cambridge University Press.

Harrison, X. A., Donaldson, L., Correa‐Cano, M. E., Evans, J., Fisher, D. N., Goodwin, C. E., ... & Inger, R. (2018). A brief introduction to mixed effects modelling and multi‐model inference in ecology. PeerJ, 6, e4794. <https://doi.org/10.7717/peerj.4794>

Zuur, A. F., Ieno, E. N., Walker, N. J., Saveliev, A. A., & Smith, G. M. (2009). Mixed Effects Models and Extensions in Ecology with R. Springer Science & Business Media.

L269-270 Why did authors chose this metric? Wouldn’t be more straightforward to present species richness and evenness? Also, does “set to NA for null abundances” means absence of one species is not considered in the model? Please explain and provide support for your decisions.

> The Shannon index is the metric that balances richness and evenness in one “diversity” metric, without favoring abundant or rare species (Jost, 2006). We used the exponential because it is more straightforward to interpret than the raw Shannon index. Indeed, the effective number of species is the virtual number of species if the community sampled was perfectly even. We added the precision to the main text, and backed it with the reference paper by Jost (2006).

“set to NA for null abundances” means that the diversity is NA if no xylem feeder was found at all. We reformulated in the text to clarify.

Jost L. Entropy and diversity. Oikos. 2006;113: 363–375. doi: 10.1111/j.2006.0030-1299.14714.x

\#####
**Results.** Authors often present their results by different region. Is this really the core, interesting aspects of their results? Of course it has important management implications, but it seems to me not as relevant as generally study vector preferences and specialization in general. Also, this approach takes a lot of space in terms of figures (fig. 2,4). I would focus on general patterns and present the regional results in the text and in the supplementary.

> Good suggestion, this is indeed more in line with the hypotheses we want to test in the article. In previous figures 2 and 4, we merged all regions to produce only one network per question (insect-habitat at nymph stage, insect-habitat at adult stage, and insect-plant species at nymph stage). The two figures were subsequently combined saving substantial space in the figures. In line with this, the “region” part of former Fig5 has been removed and is now only in the Supplementary Materials.

General organization of analyses-result presentation. Authors first focus on species specialization and then general habitat preference (abundance by habitat). In my opinion, it would be more reasonable to first explore general effect of habitat on abundance/diversity and then explore specialization.

> Thank you also for this suggestion. We organized the Results as you suggest here (first, analysis of abundance and diversity, then analysis of specialization). We re-ordered accordingly 1/ the hypotheses at the end of the Introduction, 2/ the paragraph on data analysis in M&M and 3/ the paragraphs of the Discussion.

\#####
**Discussion** generally reads well and it is well structured. I suggest the authors to better highlight the study limitations.

> We now highlight study limitations in many places of the Discussion, in response to comments made on the M&M.
