# Supplementary material for: Insect-habitat-plant interaction networks provide guidelines to mitigate the risk of transmission of Xylella fastidiosa to grapevine in Southern France
Source: PLoS One. 2025 Sep 15;20(9):e0332344. doi: 10.1371/journal.pone.0332344 (PMC12435670; doi:10.1371/journal.pone.0332344)
Supplement: Attachment — Submitted filename: Response_to_Reviewers_auresp_2.docx [file pone.0332344.s005.docx]

**PONE-D-24-38713**

**Insect-habitat-plant interaction networks provide guidelines to mitigate the risk of transmission of *Xylella fastidiosa* to grapevine in Southern France**

***Comments to the Author***

*6. Review Comments to the Author*

*Please use the space provided to explain your answers to the questions above. You may also include additional comments for the author, including concerns about dual publication, research ethics, or publication ethics. (Please upload your review as an attachment if it exceeds 20,000 characters)*

*Reviewer #1: I commend the authors for the substantial improvements made in this revised version. The manuscript is now significantly clearer, especially regarding the analytical framework and methodological structure. The authors’ willingness to engage constructively with reviewer feedback is evident and appreciated.*

🡪 Thank you for your appreciation. We believe that the suggested changes improved the manuscript.

*However, the improved clarity has also brought to light a few remaining concerns, particularly regarding the statistical modeling and analytical choices:*

*1. Network Metrics and Replication*

*It is not entirely clear why network metrics were calculated at the regional level rather than at the buffer level (Lines 260–262 / 274–275). This approach reduces the number of replicates and may limit statistical power. Since buffers are spatially independent sampling units, calculating network metrics per buffer would seem more appropriate and in line with the hierarchical sampling design. If there is a rationale for discarding buffer-level replication, it should be clearly justified in the manuscript.*

🡪 Thank you for raising this point. Indeed, we initially analyzed our data using networks computed at the buffer level, but the resulting networks were too small to be properly analyzed. We have added this information to the manuscript.

*2. Random Effects Structure*

*The use of a crossed random effect structure rather than nesting sites within buffers is questionable. Sites are always nested within buffers, so a nested random structure (site within buffer) would more accurately reflect the sampling design. The current approach may misrepresent the true data structure and introduce biases.*

🡪 We agree with your comment, and both the R code (updated on Zenodo) and the text in the M&M section were revised accordingly. Please note that this change did not affect the results presented, because the random effects were already nested by construction: site names were created by concatenating the buffer ID and the site number within each buffer.

*3. Modeling Specialization Metrics*

*Including the network metric itself as a fixed effect within a single model to generalize across metrics is unconventional. Typically, each metric would be modeled separately to avoid inflating statistical power and maintain interpretability, as was done in the supplementary analyses. If the authors decide to retain their approach, they should clearly justify it and provide published references supporting this method.*

🡪 We consider the different network metrics used to assess insect specialization as repeated measures of the same underlying phenomenon (i.e., specialization), applied to the same insects within the same networks. We deliberately selected metrics that vary in their sensitivity to network properties, ensuring that our conclusions are not overly dependent on a single metric.

To account for the potential artificial inflation of statistical power, we included a random effect on the interaction between network identity and insect species. This structure explicitly treats the specialization metrics as repeated measures taken on the same statistical unit.

In summary, while we acknowledge that this modeling approach is somewhat original, we believe it offers two key advantages: (i) presenting seven separate models /results would be confusing for the reader, and (ii) our approach remains statistically valid, as the issue of inflated power is adequately addressed within a repeated measures framework (Faraway, 2016).

In addition, as you noted in your comment, readers interested in metric-specific results can refer to Appendices 5 and 10 for the detailed analyses.

These justifications were added to the Methods section under network analysis.

Reference cited:

Faraway JJ. Extending the linear model with R : generalized linear, mixed effects and non parametric regression models. 2nd edition. Chapman & Hall/CRC Taylor & Francis group; 2016.

*7. PLOS authors have the option to publish the peer review history of their article (*[*what does this mean?*](https://track.editorialmanager.com/CL0/https:%2F%2Fjournals.plos.org%2Fplosone%2Fs%2Feditorial-and-peer-review-process%23loc-peer-review-history/1/010f0197888e57f9-13ffa924-7205-4ee7-8edc-01ed650e16ae-000000/U-0dLgLgk_tTOAMVQH_mToVcayH2ziit654ihYASwxc=216)*). If published, this will include your full peer review and any attached files.
If you choose “no”, your identity will remain anonymous but your review may still be made public.*
***Do you want your identity to be public for this peer review?*** *For information about this choice, including consent withdrawal, please see our* [*Privacy Policy*](https://track.editorialmanager.com/CL0/https:%2F%2Fwww.plos.org%2Fprivacy-policy/1/010f0197888e57f9-13ffa924-7205-4ee7-8edc-01ed650e16ae-000000/Of9Y25LP6Wx3U8lMy3S1Y-4CKG3vHnoQnkRyF83KTF8=216)*.*

*Reviewer #1: No*
